# Tocilizumab, sarilumab and anakinra in critically ill patients with COVID-19: a randomised, controlled, open-label, adaptive platform trial

**DOI:** 10.1136/thorax-2024-222488

**Published:** 2025-05-13

**Authors:** Lennie Derde, Anthony C Gordon, Paul R Mouncey, Farah Al-Beidh, Kathryn M Rowan, Alistair D Nichol, Yaseen M Arabi, Djillali Annane, Abigail Beane, Richard Beasley, Marc J M Bonten, Charlotte A Bradbury, Frank M Brunkhorst, Adrian Buzgau, Meredith Buxton, Allen C Cheng, Nicola Cooper, Matthew Cove, Olaf L Cremer, Michelle A Detry, Eamon J Duffy, Lise J Estcourt, Mark Fitzgerald, James Galea, Herman Goossens, Rashan Haniffa, Thomas E Hills, David T Huang, Nao Ichihara, Andrew King, François Lamontagne, Patrick R Lawler, Helen L Leavis, Roger J Lewis, Edward Litton, John C Marshall, Florian B Mayr, Daniel F McAuley, Anna McGlothlin, Shay P McGuinness, Bryan J McVerry, Susan C Morpeth, Srinivas Murthy, Mihai G Netea, Kayode Ogungbenro, Katrina Orr, Rachael L Parke, Jane C Parker, Asad E Patanwala, Ville Pettila, Luis Felipe Reyes, Hiroki Saito, Marlene S Santos, Christina T Saunders, Christopher W Seymour, Manu Shankar-Hari, Wendy I Sligl, Alexis F Turgeon, Anne M Turner, Steven Y C Tong, Suvi Vaara, Taryn Youngstein, Ryan Zarychanski, Cameron Green, Alisa M Higgins, Colin J McArthur, Lindsay R Berry, Elizabeth Lorenzi, Scott Berry, Steve A Webb, Derek C Angus, Frank L van de Veerdonk

**Affiliations:** 1 Intensive Care Center, University Medical Centre, Utrecht, Netherlands; 2 Imperial College London, London, UK; 3 Intensive Care National Audit and Research Centre, London, UK; 4 University College Dublin, Dublin, Ireland; 5 Critical Care, King Saud Bin Abdulaziz University for Health Sciences, Riyadh, Saudi Arabia; 6 Service de Réanimation médicale et unité de ventilation à domicile, centre de référence neuromusculaire GNHM, CHU Raymond Poincaré, APHP, Université de Versailles Saint Quentin en Yvelines, Garches, France; 7 Mahidol Oxford Tropical Medicine Research Unit, Bangkok, Thailand; 8 Nuffield Department of Clinical Medicine, University of Oxford, Oxford, UK; 9 Medical Research Institute, Wellington, New Zealand; 10 Department of Medical Microbiology, University Medical Center, Utrecht, Netherlands; 11 Julius Center for Health Sciences and Primary Care, University Medical Center, Utrecht, Netherlands; 12 University of Bristol, Bristol, UK; 13 Jena University Hospital, Jena, Germany; 14 Monash University, Melbourne, Victoria, Australia; 15 Global Coalition for Adaptive Research, Larkspur, California, USA; 16 Department of Epidemiology and Preventive Medicine, Monash University, Melbourne, Victoria, Australia; 17 Infectious Diseases Unit, Alfred Hospital, Melbourne, Victoria, Australia; 18 Imperial College London Faculty of Medicine, London, UK; 19 National University of Singapore, Singapore; 20 UMC, Utrecht, Netherlands; 21 Berry Consultants, Austin, Texas, USA; 22 Auckland City Hospital, Auckland, New Zealand; 23 University of Oxford, Oxford, UK; 24 NHS Blood and Transplant, Bristol, UK; 25 Medical Microbiology, Vaccine & Infectious Diseases Institute (VAXINFECTIO), University of Antwerp, Antwerp, Belgium; 26 University of Pittsburgh, Pittsburgh, Pennsylvania, USA; 27 The University of Tokyo, Bunkyo-ku, Tokyo, Japan; 28 Universite de Sherbrooke, Sherbrooke, Quebec, Canada; 29 McGill University Faculty of Medicine, Montreal, Quebec, Canada; 30 The University of Western Australia, Perth, Western Australia, Australia; 31 University of Toronto, Toronto, Ontario, Canada; 32 Critical Care Medicine, University of Pittsburgh Medical Center, Pittsburgh, Pennsylvania, USA; 33 ICU, QUB, Belfast, UK; 34 Royal Prince Alfred Hospital, Camperdown, New South Wales, Australia; 35 Pulmonary Allergy and Critical Care Medicine, University of Pittsburgh Medical Center Health System, Pittsburgh, Pennsylvania, USA; 36 Middlemore Hospital, Auckland, Auckland, New Zealand; 37 The University of British Columbia, Vancouver, British Columbia, Canada; 38 Radboud Universitair Medisch Centrum, Nijmegen, Netherlands; 39 The University of Manchester, Manchester, UK; 40 Fiona Stanley Hospital, Murdoch, Western Australia, Australia; 41 The University of Auckland, Auckland, New Zealand; 42 University of Helsinki, Helsinki, Finland; 43 Universidad de La Sabana, Chia, Colombia; 44 St Marianna University School of Medicine, Kawasaki, Kanagawa, Japan; 45 Unity Health Toronto, Toronto, Ontario, Canada; 46 Centre for Inflammation Research, The University of Edinburgh College of Medicine and Veterinary Medicine, Edinburgh, UK; 47 University of Alberta, Edmonton, Alberta, Canada; 48 Universite Laval, Quebec, Quebec, Canada; 49 The Peter Doherty Institute for Infection and Immunity, Melbourne, Victoria, Australia; 50 University of Manitoba, Winnipeg, Manitoba, Canada; 51 ANZIC-RC, SPHPM, Monash University Faculty of Medicine Nursing and Health Sciences, Melbourne, Victoria, Australia; 52 Monash University, Clayton, Victoria, Australia; 53 Department of Internal Medicine, Radboud University Medical Center, Nijmegen, Netherlands

**Keywords:** COVID-19, Critical Care, Pneumonia

## Abstract

**Introduction:**

Tocilizumab improves outcomes in critically ill patients with COVID-19. Whether other immune-modulator strategies are equally effective or better is unknown.

**Methods:**

We investigated treatment with tocilizumab, sarilumab, anakinra and no immune modulator in these patients. In this ongoing, adaptive platform trial in 133 sites in 9 countries, we randomly assigned patients with allocation ratios dependent on the number of interventions available at each site. The primary outcome was an ordinal scale combining in-hospital mortality (assigned –1) and days free of organ support to day 21 in survivors. The trial used a Bayesian statistical model with predefined triggers for superiority, inferiority, efficacy, equivalence or futility.

**Results:**

Of 2274 critically ill participants enrolled between 25 March 2020 and 10 April 2021, 972 were assigned to tocilizumab, 485 to sarilumab, 378 to anakinra and 418 to control. Median organ support-free days were 7 (IQR –1, 16), 9 (IQR –1, 17), 0 (IQR –1, 15) and 0 (IQR –1, 15) for tocilizumab, sarilumab, anakinra and control, respectively. Median adjusted ORs were 1.46 (95% credible intervals (CrI) 1.13, 1.87), 1.50 (95% CrI 1.13, 2.00) and 0.99 (95% CrI 0.74, 1.35) for tocilizumab, sarilumab and anakinra relative to control, yielding 99.8%, 99.8% and 46.6% posterior probabilities of superiority, respectively, compared with control. All treatments appeared safe.

**Conclusions:**

In critically ill patients with COVID-19, tocilizumab and sarilumab have equivalent effectiveness at reducing duration of organ support and death. Anakinra is not effective in this population.

**Trial registration number:**

NCT02735707.

**WHAT IS ALREADY KNOWN ON THIS TOPIC**

- In critically ill patients, tocilizumab is known to improve outcomes, including mortality. Whether other immunomodulators are more or less effective than tocilizumab is an important question.

**WHAT THIS STUDY ADDS**

- Sarilumab is similarly effective as tocilizumab at improving outcomes for critically ill patients with COVID-19. Anakinra is not effective in that population.

**HOW THIS STUDY MIGHT AFFECT RESEARCH, PRACTICE OR POLICY**

- Both tocilizumab and sarilumab can be considered for use in critically ill patients with COVID-19.

## Introduction

 Both corticosteroids and the interleukin-6 receptor antagonists (IL-6ra) tocilizumab and sarilumab reduce the need for organ support and increase survival for hospitalised patients with COVID-19.^1^,^5^ However, the comparative effectiveness of these two agents is unknown, and alternative approaches to modulation of the host inflammatory response may also be effective, particularly given issues related to drug accessibility.

Interleukin-1 (IL-1) mediates a range of cellular responses involved in acute inflammation^6^ and is, therefore, a potential therapeutic target in COVID-19.^7 8^ A recombinant form of the endogenous IL-1 receptor antagonist, anakinra, is widely used to treat autoinflammatory diseases. Anakinra treatment, using soluble urokinase plasminogen activator receptor (sUPAR) to identify patients at risk for disease progression, was effective in moderately ill patients with COVID-19 in a recent study.^9^ Coronaviruses such as SARS-CoV-2 also dampen the host interferon response to infection.^10^,^14^ Moreover, interferon-β may attenuate lung injury by enhancing endothelial barrier function.^15^,^17^

We now report the final conclusions from the immune modulation therapy domain, testing tocilizumab, sariliumab, anakinra and interferon-β for critically ill patients with COVID-19, in the Randomised, Embedded, Multifactorial Adaptive Platform Trial for Community-Acquired Pneumonia (REMAP-CAP).

## Methods

### Study design

REMAP-CAP is an international, adaptive platform trial designed to iteratively determine best treatment strategies for patients with severe pneumonia in both pandemic and non-pandemic settings and has reported on corticosteroids, antivirals, interleukin-6 receptor antagonists, anticoagulants, convalescent plasma and antiplatelet therapy in patients with COVID-19.^14 18^,^22^ The Immune Modulation Therapy Domain was open-label and was conducted in 133 hospitals in 9 countries (Australia, Canada, Finland, Ireland, Italy, the Netherlands, New Zealand, Saudi Arabia and the UK).

Patients eligible for the platform were assessed for eligibility and potentially randomised to one or more interventions across multiple domains. Domains encompass therapeutic areas and contain two or more mutually exclusive interventions (including control). Details of the trial design have been reported previously^23^ and are in the Protocol (www.remapcap.org) and Statistical Analysis Plan.

### Participants

Patients admitted to hospital, aged >18 years, with suspected or microbiologically confirmed COVID-19 were eligible for enrolment. Patients admitted to an intensive care unit (ICU) and receiving respiratory or cardiovascular organ support were classified as critically ill and all others as non-critically ill. Respiratory organ support was defined as invasive or non-invasive mechanical ventilation including via high flow nasal cannula if the flow rate was >30 L/min and the fraction of inspired oxygen was >0.4. Cardiovascular organ support was defined as receipt of any vasopressors or inotropes.

Exclusion criteria included the presumption that death was imminent with a lack of commitment to full support. Critically ill patients had to be enrolled within 24 hours of commencing organ support in ICU. Additional platform and immune modulation therapy domain exclusion criteria are listed in online supplement 1.

### Randomisation and masking

The immune modulation therapy domain included five interventions: two IL-6 receptor antagonists (tocilizumab and sarilumab), the IL-1 receptor antagonist anakinra, interferon-β1a and control (no immune modulation). Investigators at each site selected a priori at least two of the interventions to which participants could be randomised; initially, one intervention had to be control.

Participants were randomised via a centralised computer program with allocation ratios dependent on the number of interventions available at each site. The domain also permitted variation in allocation ratios based on regular adaptive analyses (response-adaptive randomisation). Patients could be randomised to additional interventions within other domains, depending on domains active at the site, patient eligibility and consent. Although clinical staff were aware of individual participant intervention assignment, neither they nor the trial steering committee were provided any information about randomisation ratios or aggregate patient outcomes.

An a priori negative interaction between interferon-β1a and corticosteroid assignment was prespecified, and clinical use of corticosteroids excluded assignment to interferon-β1a, because of a previously reported potential hazard of the combination treatment.^24^ Adaptations to this domain during the trial are shown in online supplemental figure S1.

### Procedures

Tocilizumab, at a dose of 8 mg/kg of body weight (up to a maximum of 800 mg), was administered as an intravenous infusion over 1 hour; this dose could be repeated 12–24 hours later at the discretion of the treating clinician if clinical improvement was judged insufficient. Sarilumab, 400 mg, was administered as a single intravenous infusion. Anakinra was administered intravenously as a 300 mg loading dose, followed by 100 mg every 6 hours for 14 days or until either free from invasive mechanical ventilation for more than 24 hours, or discharge from ICU. In participants who had a creatinine clearance <30 mL/min or were receiving renal replacement therapy, the anakinra dosing interval was increased to 12 hours.

All investigational drugs were dispensed by local pharmacies and were open-label.

Other aspects of patient management were provided according to each site’s standard of care. Eligibility was assessed and data were collected through an online case report form.

### Outcomes

The primary outcome was a composite ordinal scale with all deaths occurring during the index hospitalisation assigned the worst possible outcome (–1) and among survivors, respiratory and cardiovascular organ support-free days were calculated up to day 21, such that higher numbers represent better outcomes. Secondary and exploratory outcomes were all prespecified and are listed in online supplement 1.

Safety assessment and reporting were conducted as described in the Protocol by local investigators. Adverse events that were part of the primary outcome (ie, mortality and the need for organ support) were not reported as adverse events unless assessed to be at least possibly related to the intervention.

### Statistical analysis

REMAP-CAP uses a Bayesian design with no maximum sample size. Regular adaptive analyses are performed, and randomisation continues until predefined statistical criteria for domain stopping are met.

The primary analysis was a Bayesian cumulative logistic model, which calculated posterior probability distributions of the organ support-free days (primary outcome) based on evidence accumulated in the trial and prior probability distributions. Prior distributions for treatment effects were centred on no effect as prespecified.^23^

The model adjusted for location (site as a random effect nested within country), age (categorised into six groups), sex and time period (2 weeks calendar epochs) to account for rapid changes in clinical care and outcomes over time during the pandemic. The temporal effects adjust for changes in organ support-free days over time^25^ and allow for estimation of each intervention’s average treatment effect across time periods.^26 27^

The primary analysis (online supplement 2) included all participants with suspected or proven COVID-19 randomised to any domain up to 10 April 2021, who had completed at least 21 days follow-up, for whom an outcome was known. Data from participants enrolled outside the immune modulation therapy domain provided more robust estimations of adjustment covariates but did not contribute to estimates of immune modulation treatment effects.^23^

The model was fit using a Markov Chain Monte Carlo algorithm (20 000 draws) that calculated the posterior distribution of the proportional ORs for each intervention, including medians and 95% credible intervals (CrI). An OR >1 reflected an increase in the cumulative odds for the organ support-free days outcome, implying benefit. Missing outcomes were not imputed.

Several sensitivity analyses of the primary analysis model were conducted. The model was run without the site and time adjustments and separately run within the per protocol population and the population restricted to participants enrolling in the immune modulation therapy domain.

The predefined statistical triggers for trial conclusions and disclosure of results are described in the Statistical Appendix to the protocol (www.remapcap.org). Further details of all analyses are provided, and prespecified analyses are listed in online supplemental files 1-5.

Data management and summaries were created with the use of R software, V.3.6.0; the primary analysis was computed with R software, V.4.0.0, with the use of the rstan package, V.2.21.1. Additional data management and analyses were performed with SQL Server 2016; SPSS software, V.26; and Stata software, V.14.2. The trial has a data safety and monitoring board.

## Results

**Figure 1:**
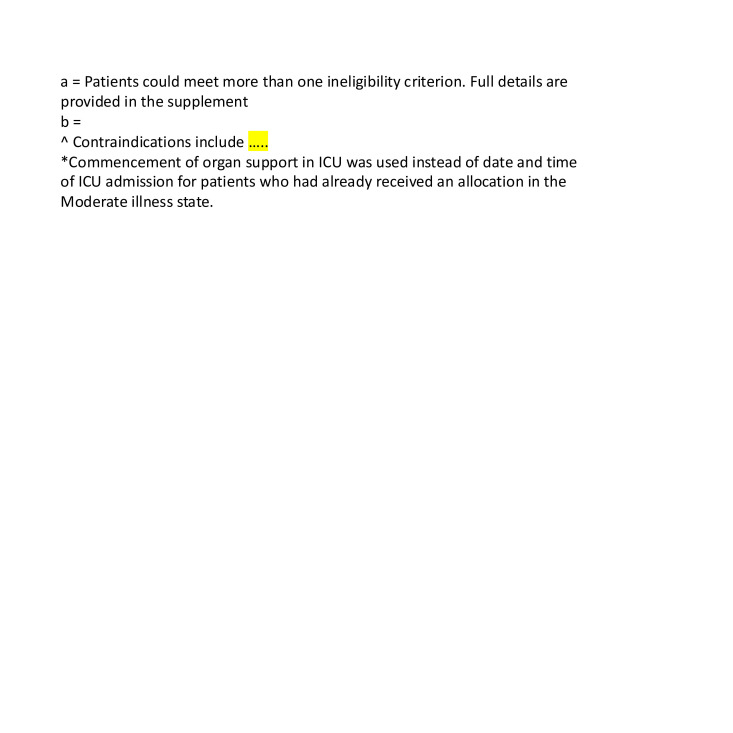
Screening, enrolment, randomisation and inclusion in analysis. ^a^Patients could meet more than one ineligibility criterion. Full details are provided in online supplement 1. ^b^Contraindications include known hypersensitivity to an agent specified as an intervention; known or suspected pregnancy; known hypersensitivity to proteins produced by *Escherichia coli* (exclusion from receiving anakinra); baseline alanine aminotransferase or aspartate aminotransferase that is more than five times the upper limit of normal (exclusion from receiving tocilizumab or sarilumab); baseline platelet count <50×10^9^/L (exclusion from receiving tocilizumab or sarilumab). *Commencement of organ support in ICU was used instead of date and time of ICU admission for patients who had already received an allocation in the Moderate State. ICU, intensive care unit; REMAP-CAP, Randomised, Embedded, Multifactorial Adaptive Platform Trial for Community-Acquired Pneumonia.

The first patient was included in the immune modulation therapy domain on 25 March 2020. The control arm was closed on 19 November 2020 when the statistical trigger for superiority of tocilizumab compared with control was met. Statistical triggers for equivalence between tocilizumab and sarilumab; and for inferiority of anakinra to the other active interventions in critically ill participants were met at a planned adaptive analysis on 9 April 2021 following which all arms of the domain were closed. At that time, 6023 participants had been randomised in at least one domain in REMAP-CAP and 2274 critically ill participants had been randomised in the immune modulation therapy domain (972 tocilizumab, 485 sarilumab, 378 anakinra, 21 interferon-β1a and 418 control) in 133 sites across 9 countries (figure 1). 39 of these participants subsequently withdrew consent, and 19 had missing primary outcomes. Only five non-critically ill participants were randomised, and results for these participants and the 21 patients assigned to interferon-β1a are reported in online supplemental tables S1–S5.

Baseline characteristics for critically ill participants were balanced across intervention groups (table 1 and online supplemental tables S1–2). All but four participants were receiving respiratory support at the time of randomisation, including high flow nasal oxygen (536/2235, 24%), non-invasive (958/2235; 42.9%) and invasive (735/2235; 32.9%) mechanical ventilation. There were a small number of patients with any immunosuppressed condition (2.7%), and 81.5% of patients and 28.7% of patients were on corticosteroids and remdesivir at randomisation.

**Table 1:** Participant characteristics at baseline (severe state)*

	Tocilizumab (n=952)	Sarilumab (n=485)	Anakinra (n=373)	Control (n=406)
Age—mean (SD), years	60.8 (12.2)	59.0 (13.2)	59.8 (11.9)	61.1 (12.9)
Male sex—n (%)	656 (68.9)	326 (67.2)	269 (72.1)	285 (70.2)
Race/ethnicity†—n/N (%)				
White	515/724 (71.1)	368/453 (81.2)	184/254 (72.4)	234/317 (73.8)
Asian	123/724 (17.0)	53/453 (11.7)	39/254 (15.4)	53/317 (16.7)
Black	38/724 (5.2)	9/453 (2.0)	9/254 (3.5)	10/317 (3.2)
Mixed	13/724 (1.8)	1/453 (0.2)	6/254 (2.4)	6/317 (1.9)
Other	35/724 (4.8)	22/453 (4.9)	16/254 (6.3)	14/317 (4.4)
Body-mass index‡—median (IQR), kg/m^2^	30.4 (26.6–34.9) (n=862)	31.2 (27.7–36.3) (n=419)	29.7 (26.3–35.3) (n=332)	30.9 (27.1–34.9) (n=385)
APACHE II score§**—median (IQR)	13.0 (8.0–19.0) (n=934)	12.0 (7.0–20.0) (n=475)	13.0 (8.0–19.0) (n=365)	12.0 (8.0–18.0) (n=394)
Confirmed SARS-CoV-2 infection¶—n/N (%)	802/942 (85.1)	429/484 (88.6)	319/369 (86.4)	348/406 (85.7)
Pre-existing condition—n/N (%)				
Diabetes	281/949 (29.6)	108/484 (22.3)	125/370 (33.8)	152/406 (37.4)
Respiratory disease	218/949 (23.0)	117/484 (24.2)	81/370 (21.9)	100/406 (24.6)
Asthma/COPD	183/949 (19.3)	99/484 (20.5)	67/370 (18.1)	89/406 (21.9)
Other	40/949 (4.2)	26/484 (5.4)	17/370 (4.6)	17/406 (4.2)
Kidney disease	66/866 (3.2)	30/446 (1.5)	22/340 (1.1)	43/377 (2.1)
Severe cardiovascular disease	86/930 (9.2)	33/474 (7.0)	41/367 (11.2)	47/401 (11.7)
Any immunosuppressive condition	25/948 (2.6)	11/484 (2.3)	6/370 (1.6)	18/406 (4.4)
Cancer	7/948 (0.7)	3/484 (0.6)	3/370 (0.8)	10/406 (2.5)
Chronic immunosuppressive therapy	10/949 (1.1)	8/484 (1.7)	4/370 (1.1)	7/406 (1.7)
Other	13/948 (1.4)	2/484 (0.4)	3/370 (0.8)	5/406 (1.2)
Liver cirrhosis/failure	3/930 (0.3)	0/474 (0.0)	1/370 (0.3)	2/401 (0.5)
Time to enrolment—median (IQR)				
From hospital admission—days	1.4 (0.9–3.3)	1.6 (0.9–3.5)	1.6 (0.9–3.8)	1.2 (0.8–2.8)
From ICU admission—hours	13.4 (6.9–19.1)	15.1 (7.8–19.9)	13.6 (7.3–19.7)	14.0 (6.8–19.5)
Acute respiratory support—n (%)				
None/supplemental oxygen only	1 (0.1)	0 (0.0)	1 (0.3)	2 (0.5)
High-flow nasal cannula	226 (23.7)	96 (19.8)	101 (27.1)	110 (27.1)
Non-invasive ventilation only	404 (42.4)	241 (49.7)	133 (35.7)	171 (42.1)
Invasive mechanical ventilation	320 (33.6)	148 (30.5)	138 (37.0)	122 (30.0)
ECMO	1 (0.1)	0 (0.0)	0 (0.0)	1 (0.2)
Vasopressor support—n (%)	179 (18.8)	77 (15.9)	81 (21.7)	79 (19.5)
PaO_2_/FiO_2_—median (IQR)	110 (86–148) (n=872)	116 (89–152) (n=430)	106 (84–148) (n=330)	118 (89–169.5) (n=359)
Median laboratory values (IQR)**				
C reactive protein, µg/mL	132 (69–201) (n=783)	120 (70–199) (n=419)	112 (70–189) (n=324)	129 (71–208) (n=255)
D-dimer, µg/L	946 (483–2475) (n=564)	947 (420–2216) (n=304)	1006 (460–2363) (n=256)	1010 (500–2115) (n=175)
Received therapies at randomisation—n/N (%)				
Corticosteroids	770/938 (82.1)	422/472 (89.4)	317/369 (85.9)	269/402 (66.9)
Remdesivir	272/938 (29.0)	140/472 (29.7)	109/369 (29.5)	105/402 (26.1)

* Percentages may not sum to 100 because of rounding.

† Data collection is not approved in Canada and continental Europe. ‘Other’ includes ‘declined’ and ‘multiple’.

‡ Body mass index is the weight in kilograms divided by the square of the height in metres.

§ Range 0–71, with higher scores indicating greater severity of illness.

¶ SARS-CoV2 infection was confirmed by respiratory tract PCR test.

** Values were from the sample collected closest to randomisation, up to 8 hours prior to randomisation. If no samples were collected up to 8 hours prior to time of randomisation, the sample collected closest to the time of randomisation up to 2 hours after randomisation was used (other than PaO2/FiO2 which was a prerandomisation value only).

APACHE, Acute Physiology and Chronic Health Evaluation; COPD, chronic obstructive pulmonary disease; ECMO, extracorporeal membrane oxygenation; FiO2, fractional inspired oxygen; ICU, intensive care unit; PaO2, arterial oxygen tension.

**Table 2:** Primary and secondary outcomes

Outcome/analysis	Tocilizumab (N=943)	Sarilumab (N=483)	Anakinra (N=365)	Control (N=406)
Primary outcome				
Median (IQR)	7 (–1 to 16)	9 (–1 to 17)	0 (–1 to 15)	0 (–1 to 15)
Adjusted OR—mean (SD)	1.47 (0.19)	1.52 (0.22)	1.00 (0.16)	1
Median (95% CrI)	1.46 (1.13 to 1.87)	1.50 (1.13 to 2.00)	0.99 (0.74 to 1.35)	1
Probability of superiority to control, %	99.8	99.8	46.6	–
Probability of equivalence (tocilizumab/sarilumab), %	84.9			–
Probability of being the optimal intervention, %*	37.6	62.4	0.03	<0.01
Subcomponents of primary outcome				
In-hospital deaths, n (%)	317/943 (33.6)	158/483 (32.7)	145/365 (39.7)	150/406 (36.9)
Days free from organ support in survivors, median (IQR)	15 (7.25 to 18)	15 (9 to 18)	14 (3.75 to 18)	13 (4 to 17)
Primary hospital survival				
Adjusted OR—mean (SD)	1.44 (0.23)	1.54 (0.29)	0.99 (0.19)	1
Median (95% CrI)	1.42 (1.05 to 1.93)	1.51 (1.06 to 2.20)	0.97 (0.66 to 1.40)	1
Probability of superiority to control, %	98.8	98.8	43.6	–
Secondary analysis of primary outcome				
Adjusted OR—mean (SD)	1.50 (0.19)	1.59 (0.22)	1.07 (0.17)	1
Median (95% CrI)	1.49 (1.16 to 1.91)	1.57 (1.20 to 2.06)	1.06 (0.78 to 1.44)	1
Probability of superiority to control, %	99.9	99.9	64.6	–
Secondary analysis of hospital survival				
Adjusted OR—mean (SD)	1.45 (0.23)	1.59 (0.29)	1.03 (0.20)	1
Median (95% CrI)	1.43 (1.05 to 1.95)	1.57 (1.10 to 2.22)	1.01 (0.70 to 1.49)	1
Probability of superiority to control, %	99.0	99.3	52.9	–

The primary analysis of organ support-free days and in-hospital mortality used data from all participants (moderate and severe state) enrolled in the trial who met COVID-19 criteria and were randomised within at least one domain, for whom the outcome was known (n=5852), adjusting for age, sex, time period, site, region, domain and intervention eligibility and intervention assignment.

Secondary analyses were restricted to n=3848 participants enrolled in the immune modulation therapy domain and any domains that have ceased recruitment (corticosteroid; COVID-19 antiviral, anticoagulation and immunoglobulin domains), adjusting for age, sex, time period, site, region, domain and intervention eligibility and intervention assignment. Definitions of outcomes are provided in Methods and the study protocol. All models are structured such that a higher OR or HR is favourable.

* The posterior probability that pooled IL-6ra (calculated from the secondary model) is optimal in the domain was >99.9%. Given the small number of patients randomised to the interferon-β1a intervention, this posterior probability calculation was restricted to the interventions with adequate data support (control, tocilizumab, sarilumab and anakinra).

CrI, credible intervals.

Adherence to allocated intervention was defined as receipt of at least one dose of the allocated drug for the active interventions and receiving no immune modulator for the control arm. Adherence was 96.7% for tocilizumab, 96.0% for sarilumab and 94.6% for anakinra. Of 906 patients receiving tocilizumab, 295 received more than one dose (32.6%). Of participants allocated to the control group, 7/402 (1.7%) received one or more of the drugs available in this domain.

**Figure 2:**
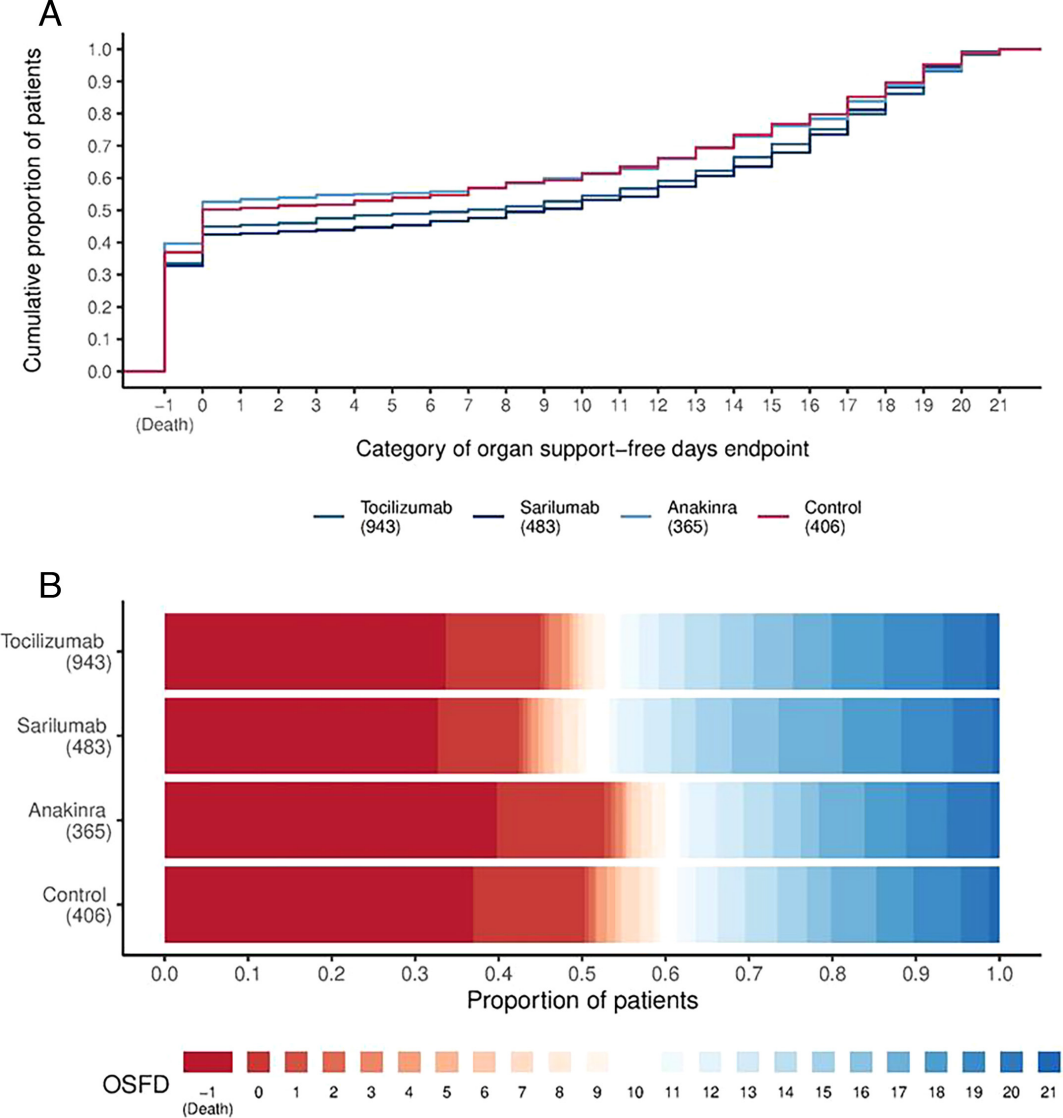
Distributions of organ support-free days. (A) The cumulative proportion (y-axis) for each intervention group by day (x-axis), with death listed first. Curves that rise more slowly indicate a more favourable distribution in the number of days alive and free of organ support. The height of each curve at ‘–1’ indicates the in-hospital mortality rate for each intervention. The height of each curve at any point, for example, at day=10, indicates the proportion of patients with organ support-free days (OSFD) of 10 or lower (ie, 10 or worse). The difference in height of the two curves at any point represents the difference in the percentile in the distribution of OSFDs associated with that number of days alive and free of organ support. (B) Organ support-free days as horizontally stacked proportions by intervention group. Red represents worse outcomes and blue represents better outcomes. The median adjusted ORs from the primary analysis, using a Bayesian cumulative logistic model, were 1.46 (95% CrI 1.13, 1.87) for tocilizumab, 1.50 (95% CrI 1.13, 2.00) for sarilumab and 0.99 (95% CrI 0.74, 1.35) for anakinra, yielding 99.8%, 99.8% and 46.5% posterior probabilities of superiority, respectively, compared with control. CrI, credible intervals.

Median organ support-free days were 7 (IQR –1, 16), 9 (IQR –1, 17), 0 (IQR –1, 15) for tocilizumab, sarilumab and anakinra compared with 0 (IQR –1, 15) for the control group (table 2 and figure 2). Compared with control, median adjusted ORs for organ support-free days were 1.46 (95% CrI 1.13, 1.87) for tocilizumab, 1.50 (95% CrI 1.13, 2.00) for sarilumab and 0.99 (95% CrI 0.74, 1.35) for anakinra, yielding 99.8%, 99.8% and 46.6% posterior probabilities of superiority to control, respectively. The posterior probability of equivalence between tocilizumab and sarilumab, defined as a relative OR between 1/1.2 and 1.2, was 84.9% and the probability that sarilumab was non-inferior to tocilizumab was 98.9% (online supplemental figure S2). The probability that each intervention was optimal (ie, the best among those compared) was 38% for tocilizumab, 62% for sarilumab, 0.3% for anakinra and <0.1% for control.

Hospital survival rates were 66.4% for tocilizumab, 67.3% for sarilumab, 60.3% for anakinra and 63.1% for control. Compared with control, median adjusted ORs for hospital survival were 1.42 (95% CrI 1.05, 1.93) for tocilizumab, 1.51 (95% CrI 1.06, 2.20) for sarilumab and 0.97 (95% CrI 0.66, 1.40) for anakinra, yielding 98.8%, 98.8% and 43.6% respective posterior probabilities of the interventions being superior to control for this outcome (online supplemental figure S3).

**Figure 3:**
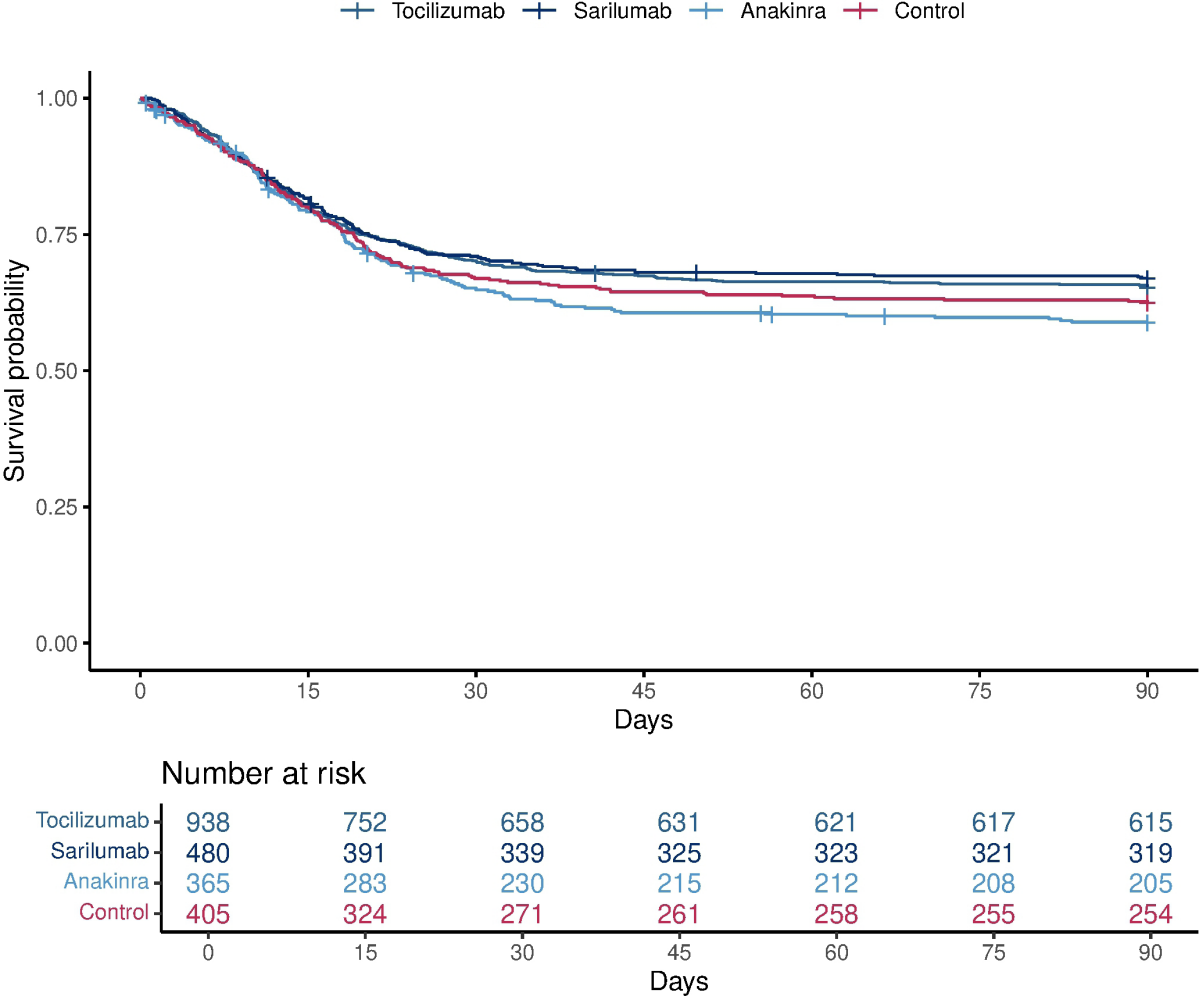
Time-to-event analysis. This plot is restricted to participants in the severe state randomised to the immune modulation therapy domain or another unblinded domain. The Kaplan-Meier curve for survival up to 90 days according to individual interventions is shown. There were 323, 161, 160 and 151 deaths in the tocilizumab, sarilumab, anakinra and control groups, respectively. This resulted in an HR of 1.39 (95%CrI 1.11 to 1.74) for tocilizumab, 1.44 (95% CrI 1.11 to 1.89) for sarilumab and 1.13 (95% CrI 0.87 to 1.49) for anakinra, yielding 99.9%, 99.6% and 82.3% respective posterior probabilities of superiority to control. These ‘survival HRs’ are defined as the reciprocal of the mortality HR to be consistent with the convention that ORs andHRs >1 imply benefit.

Tocilizumab and sarilumab were both effective across all secondary outcomes, including 90-day survival (99.9% and 99.6% probability of superiority to control, respectively), and both led to more rapid ICU and hospital discharge (figure 3 and online supplemental table S4). The ORs for organ support-free days for tocilizumab and sarilumab were consistent across models with nested, independent and pooled treatment effects (online supplemental figure S4). There was no evidence of an effect of anakinra in any of the secondary outcome analyses. The rates of serious adverse events were similar between all interventions (online supplemental table S5).

The effects of both tocilizumab and sarilumab were similar for participants who were and were not invasively mechanically ventilated and across CRP terciles (online supplemental table S6 and 7). Subgroup results for anakinra showed no beneficial effect (online supplemental figure S5 and S6). The sensitivity analyses on the primary outcome were consistent with the primary analysis (online supplemental table S3).

## Discussion

REMAP-CAP is the first international, multicentre, randomised, adaptive platform trial designed to evaluate the potential benefit and safety of tocilizumab, sarilumab and anakinra, compared with standard of care in adult patients with COVID-19 receiving organ support in intensive care. This trial is the first to provide direct comparisons between these drugs.

REMAP-CAP has previously reported the efficacy of tocilizumab and sarilumab compared with standard of care in critically ill patients.^4^ The RECOVERY trial similarly showed the effectiveness of tocilizumab in a broader group of hospitalised patients.^28^ Benefits of IL-6ra are consistent across primary and secondary outcomes, and across subgroups and secondary analyses. There has been less evidence about the efficacy of sarilumab.^5^ Here, we report that these two drugs are equally effective when compared directly.

The comparison met the trial criteria for equivalence with a posterior probability of >90% at the time of a planned adaptive analysis; it fell below that threshold as full data became available because of an increase in the likelihood that sarilumab was superior to tocilizumab. In the face of an urgent global need and potential limitations of drug supply, the two agents can be considered equally effective, and this can help ensure as many patients as possible receive effective treatments.

We did not observe a beneficial effect of anakinra in critically ill patients with COVID-19. Previous studies and a meta-analysis provided a rationale for the use of anakinra in COVID-19.^29^ There are several possible explanations for our findings. First, we could have chosen the wrong dose. However, our choice of administration regimen was informed by pharmacometric modelling data, and it is unlikely that predicted drug concentrations varied significantly. Second, blocking IL-1 may benefit non-critically ill patients, but not critically ill patients. Such potential differential effects based on illness severity could not be assessed in this study as a result of the low recruitment in non-critically ill patients. However, there are suggestions that neutralising autoantibodies against the IL-1 receptor antagonist (IL-1ra) are produced during COVID-19.^14 30^ Since anakinra is recombinant IL-1ra, its effectiveness may depend on the level of these auto-antibodies, which may differ with disease severity. Finally, we did not use a strategy of early targeting of the IL-1 pathway in selected patients, as used in the SAVE-MORE trial,^9^ because sUPAR is not commonly available. We did not investigate the effectiveness of baricitinib, an oral selective Janus kinase 1/2 inhibitor, in our trial. Adding baricitinib to standard of care (including dexamethasone) was associated with reduced mortality in hospitalised adults with COVID-19.^31 32^

REMAP-CAP’s pragmatic, international design means that our results are likely generalisable to the wider critically ill patient population with COVID-19. Importantly, even once we demonstrated individual benefits of tocilizumab and sarilumab, the adaptive design allowed continued randomisation to evaluate the comparative effectiveness of these two interventions, as well as to continue evaluation of other immune modulation therapies. This allowed maximal learning about treatment effects while improving standard care, having removed the less effective control group from further assignment.^33^

However, continuing randomisation to the immune modulation strategies after halting the control group does introduce some complexities into the interpretation of the trial results. It is possible that temporal trends were present in patient outcomes over time due to, for example, changing variants, background care, and availability of vaccinations. In certain circumstances, these temporal trends could result in bias in treatment effect estimates if naïve analysis methods are applied. To address this possibility of temporal trends, the primary analysis model included a temporal adjustment for each 2-week period of calendar time during which randomisation occurred. Recent papers have demonstrated that this type of flexible temporal adjustment has minimal risk of bias or type I error inflation even in the presence of time trends, and can result in greater precision than alternative approaches that discard data from periods in which randomisation to control was not active.^25 34^ Thus, we believe the presented model estimates to be generally robust against temporal trends; however, some caution may be warranted when interpreting unadjusted data summaries such as the median OSFD and mortality rates summarised in table 2.

The trial has limitations. It uses an open-label design, although awareness of intervention assignment is unlikely to affect the mortality component of the primary outcome. The effect of interferon-β1a could not be assessed with only 21 patients randomised to this intervention, as the use of corticosteroids excluded patients from this treatment. Interferon-β1a did not improve clinical outcomes in the SOLIDARITY Trial.^35^

Future research should be aimed at investigating the effect of other immune modulators, like baricitinib, in the critically ill population with COVID-19, and potentially other viral pneumonias. The effectiveness of anakinra treatment in the SAVE-MORE trial compared with the lack of effect in this study warrants further exploration. We should aim to better understand the pathophysiological mechanisms, so we can identify in the subpopulation of hospitalised COVID-19 patients who can benefit from anakinra treatment.

In conclusion, in adult patients with COVID-19 receiving organ support in intensive care, the IL-6 receptor antagonists, tocilizumab and sarilumab, have equivalent effectiveness at improving survival and reducing duration of organ support. Anakinra is not effective in this population.

## Supplementary material

10.1136/thorax-2024-222488 online supplemental file 1

10.1136/thorax-2024-222488 online supplemental file 2

10.1136/thorax-2024-222488 online supplemental file 3

10.1136/thorax-2024-222488 online supplemental file 4

10.1136/thorax-2024-222488 online supplemental file 5

## Data Availability

Data are available on reasonable request.
