# Supplementary material for: Tocilizumab, sarilumab and anakinra in critically ill patients with COVID-19: a randomised, controlled, open-label, adaptive platform trial
Source: Thorax. 2025 May 13;80(8):e222488. doi: 10.1136/thorax-2024-222488 (PMC12322455; doi:10.1136/thorax-2024-222488)
Supplement: online supplemental file 3 [file thorax-80-8-s003.pdf]

REMAP-CAP Unblinded Secondary Analysis Report for the Immune Modulation Domain

Prepared by the ITSC Analysis Committee

June 10, 2021

Contents

1 Introduction **2**

1.1 Immune Modulation domain interventions . . . 2

1.2 Analysis populations . . . 3

1.3 Modeling conventions . . . 5

1.4 Overall summaries of OSFD in the Unblinded ITT population . . . 6

2 Secondary analyses of OSFD **8**

2.1 Empirical distribution of OSFD in Immune Modulation ITT population . . . 8

2.2 Summary of OSFD odds ratios for IL-6ra interventions . . . 11

2.3 Secondary analysis of OSFD for Unblinded ITT population with nesting between IL-6ra intervention effects . . . 11

2.4 Secondary analysis of OSFD for Unblinded ITT population with independent IL-6ra intervention effects . . . 13

2.5 Secondary analysis of OSFD for Unblinded ITT population with pooled IL-6ra effect . . . 15

2.6 Secondary analysis of OSFD for Unblinded ITT population with additional unblinded interaction effects . . . 17

2.7 Secondary analysis of OSFD for Unblinded ITT population restricted to non-negative COVID-19 20

2.8 Secondary analysis of OSFD for Immune Modulation ITT population . . . 22

2.9 Sensitivity analysis of OSFD for Unblinded ITT population with site and time factors removed 24

3 Secondary analyses of in-hospital mortality **26**

3.1 Empirical distribution of in-hospital mortality in Immune Modulation ITT population . . . 27

3.2 Summary of hospital survival odds ratios for IL-6ra interventions . . . 27

3.3 Secondary analysis of hospital survival for Unblinded ITT population with nesting between IL-6ra intervention effects . . . 27

3.4 Secondary analysis of hospital survival for Unblinded ITT population with independent IL-6ra intervention effects . . . 29

3.5 Secondary analysis of hospital survival for Unblinded ITT population with pooled IL-6ra effect 31

3.6 Secondary analysis of hospital survival for Unblinded ITT population with additional unblinded interaction effects . . . 33

3.7 Secondary analysis of hospital survival for Unblinded ITT population restricted to non-negative COVID-19 . . . 35

3.8 Secondary analysis of hospital survival for Immune Modulation ITT population 37
3.9 Sensitivity analysis of hospital survival for Unblinded ITT population with site and time factors removed 39

4 Safety analyses **41**
4.1 Empirical distribution of serious adverse events in Immune Modulation ITT population . . . 42
4.2 Primary safety analysis of serious adverse events in Immune Modulation ITT population . . 42

5 Per protocol analyses **43**
5.1 Data summaries for per protocol analyses 43
5.2 Sensitivity analysis of OSFD for Immune Modulation per protocol population 45
5.3 Sensitivity analysis of hospital survival for Immune Modulation per protocol population . . 46

6 Analyses of secondary endpoints **48**
6.1 Secondary analysis of 90-day mortality 48
6.2 Secondary analysis of progression to intubation, ECMO, or death 51
6.3 Secondary analysis of cardiovascular support-free days 54
6.4 Secondary analysis of respiratory support-free days 57
6.5 Secondary analysis of length of ICU stay 60
6.6 Secondary analysis of length of hospital stay 63
6.7 Secondary analysis of WHO scale at 14 days 66
6.8 Secondary analysis of major thrombotic events or death 70
6.9 Secondary analysis of major thrombotic events 72
6.10 Secondary analysis of major bleeding events 74

7 Subgroup analyses **75**
7.1 Subgroup analyses by baseline C-reactive protein subgroup 75
7.2 Subgroup analyses by baseline mechanical ventilation status 86

8 Report production and data sources **95**

1 Introduction

This report summarizes the data and the results for the Immune Modulation Therapy domain analyses run by the ITSC analysis committee. The ITSC analysis committee is blinded to all ongoing domains and interventions in REMAP-CAP. The data and analyses in this report are limited to domains/interventions that have been unblinded. All analyses involving blinded randomized assignments, like the primary analysis, are in the primary analysis report created by the unblinded Statistical Analysis Committee (SAC).

1.1 Immune Modulation domain interventions

There are five interventions in the Immune Modulation Therapy Domain. These are:

1. **Control** (No immune modulation for COVID-19)

2. Interferon-beta-1a (IFN-β1a)
3. Anakinra (interleukin-1 receptor antagonist; IL-1ra)
4. Tocilizumab (IL-6 receptor antagonist; IL-6ra)
5. Sarilumab (IL-6 receptor antagonist; IL-6ra)

These five interventions are mutually exclusive; patients randomized in this domain are assigned to one of the five interventions. As a result of meeting the statistical trigger for equivalence, the two IL-6ra interventions are pooled into a single intervention labeled **Pooled IL-6ra** in some models/tables/figures.

1.2 Analysis populations

The SAP for the immune modulation domain analysis defines five analysis populations. This report includes results for four of the five analysis populations. The analysis results for the **REMAP-CAP COVID-19 severe and moderate intent-to-treat (ITT)** population are not included in this report since the authors remain blinded to assingments in ongoing domains and interventions. In this report, tables and figures summarize data in the **Immune Modulation specific ITT population**. This report includes data and analyses from the following four analysis populations:

1. The **Unblinded ITT population** is defined as all severe patients randomized in one or more unblinded domains within the pandemic stratum without a prior randomization in the moderate state. The unblinded ITT population consists of 3848 patients randomized in the Immune Modulation Therapy, Corticosteroid, Antiviral, Anticoagulation, or Immunoglobulin domains. There are 31 patients within this population that are missing values of OSFD and in-hospital mortality.

- 2226 patients randomized to the Immune Modulation domain
  - 405 patients randomized to **control** of which 405 have known OSFD outcome
  - 19 patients randomized to **interferon-beta-1a** of which 19 have known OSFD outcomes
  - 373 patients randomized to **anakinra** of which 365 have known OSFD outcomes
  - 947 patients randomized to **tocilizumab** of which 938 have known OSFD outcomes
  - 482 patients randomized to **sarilumab** of which 480 have known OSFD outcomes
- 386 patients randomized to the Corticosteroid domain of which 379 have known OSFD outcomes
  - 3339 patients randomized after the closure of the Corticosteroid domain
- 693 patients randomized to the Antiviral domain of which 691 have known OSFD outcomes.
- 1010 patients randomized to the Anticoagulation domain of which 1007 have known OSFD outcomes.
- 1980 patients randomized to the Immunoglobulin domain of which 1973 have known OSFD out-

comes.

2. The **Unblinded non-negative COVID-19 population** is defined as all patients in the Unblinded ITT population after removing those with >1 negative test for COVID-19 and no positive tests. The unblinded ITT population restricted to non-negative COVID-19 consists of 3613 patients. There are 29 patients within this population that are missing values of OSFD and in-hospital mortality.

- 2096 patients randomized to the Immune Modulation domain
  - 364 patients randomized to **control** of which 364 have known OSFD outcome
  - 15 patients randomized to **interferon-beta-1a** of which 15 have known OSFD outcomes
  - 363 patients randomized to **anakinra** of which 355 have known OSFD outcomes
  - 887 patients randomized to **tocilizumab** of which 879 have known OSFD outcomes
  - 467 patients randomized to **sarilumab** of which 465 have known OSFD outcomes
- 316 patients randomized to the Corticosteroid domain of which 310 have known OSFD outcomes
  - 3203 patients randomized after the closure of the Corticosteroid domain
- 614 patients randomized to the Antiviral domain of which 612 have known OSFD outcomes.
- 955 patients randomized to the Anticoagulation domain of which 952 have known OSFD outcomes.
- 1955 patients randomized to the Immunoglobulin domain of which 1948 have known OSFD outcomes.

3. The **Immune Modulation ITT population** consists of patients in the Unblinded ITT population that were randomized to the Immune Modulation domain within the pandemic stratum. The Immune Modulation ITT population consists of 2226 patients. There are 19 patients within this population that are missing values of OSFD and in-hospital mortality.

- 405 patients randomized to **control** of which 405 have known OSFD outcomes
- 19 patients randomized to **interferon-beta-1a** of which 19 have known OSFD outcomes
- 373 patients randomized to **anakinra** of which 365 have known OSFD outcomes
- 947 patients randomized to **tocilizumab** of which 938 have known OSFD outcomes
- 482 patients randomized to **sarilumab** of which 480 have known OSFD outcomes

4. The **Immune Modulation Per Protocol (PP) population** consists of the patients in the Immune Modulation ITT population who have been treated as per protocol. The Immune Modulation per protocol population consists of 2107 patients. There are 13 patients within this population that are missing values of OSFD and in-hospital mortality.

- 394 patients randomized to **control** of which 394 have known OSFD outcomes
- 14 patients randomized to **interferon-beta-1a** of which 14 have known OSFD outcomes
- 349 patients randomized to **anakinra** of which 343 have known OSFD outcomes
- 901 patients randomized to **tocilizumab** of which 896 have known OSFD outcomes
- 449 patients randomized to **sarilumab** of which 447 have known OSFD outcomes

1.3 Modeling conventions

- All reported credible intervals (CrIs) are 95% equal-tailed intervals.
- Results from models of ordinal and dichotomous endpoints are reported as odds ratios (ORs). Results from models of time to event endpoints are reported as hazard ratios (HRs). **For consistency of interpretation, all models are parameterized so that an OR/HR greater than 1 indicates patient benefit relative to the reference group and an OR/HR less than 1 indicates patient harm relative to the reference group.**
- The reference group for the age category OR/HRs is the age category from 60-69 years old.
- The reference group for the time epochs OR/HRs is the most recent time epoch consisting of the four-week period preceding Apr 9, 2021. Time epoch 0 is the most recent epoch and the epochs move backwards in time in two-week periods from epoch 1 to 27.
- The reference group for the sex at birth OR/HRs is the male category.
- **Interferon-beta-1a**, **Anakinra**, **Tocilizumab**, and **Sarilumab** are compared to the **Control** intervention. A posterior probability of at least 99% that the odds ratio is greater than 1 is used as a statistical trigger for efficacy (or superiority to control). Similarly, a probability of harm is reported as the probability that the odds ratio is less than 1 relative to **Control**, or 1 minus the probability of efficacy.
- **Interferon-beta-1a**, **Anakinra**, **Tocilizumab**, and **Sarilumab** are compared to **Control** for futility. A 95% probability of a smaller than 1.2 odds ratio for **Interferon-beta-1a**, **Anakinra**, **Tocilizumab**, and **Sarilumab** relative to **Control** is used as a statistical trigger for futility.
- Pairwise comparisons of **Interferon-beta-1a**, **Anakinra**, **Tocilizumab**, and **Sarilumab** are completed to assess equivalence. A 90% probability of equivalence (defined as an odds ratio of two active interventions between 1/1.2 and 1.2) is used as a statistical trigger for equivalence.
- For each intervention in the Immune Modulation domain, the probability of being in the optimal regimen is calculated and used for statistical triggers of domain superiority and inferiority. A greater than 99% probability of being in the optimal regimen is used as a statistical trigger for superiority for each active Immune Modulation intervention. A less than 0.33% probability of being in the optimal

regimen is used as a statistical trigger of inferiority for all Immune Modulation interventions.

- OR/HR effects for pre-specified **combinations** of interventions from the Immune Modulation domain with the Corticosteroid and Antiviral domain are reported relative to receiving control in each domain. These OR/HRs incorporate the effect of each individual intervention and the interaction term for the combination of interventions.
- OR/HR effects for pre-specified **interaction effects** are reported relative to an additive effect (on the log odds ratio scale). For example, if an interaction effect OR/HR is equal to 1, the combination of interventions is additive (equal to the sum of the log OR effects of the two interventions taken separately). If an interaction OR/HR is greater than 1, the effect of the combination of interventions is synergistic (greater than the sum of the log OR effects of the two interventions taken separately). If the interaction OR/HR is less than 1, the effect of the combination is sub-additive (less than the sum of the log OR effects of the two interventions taken separately).

1.4 Overall summaries of OSFD in the Unblinded ITT population

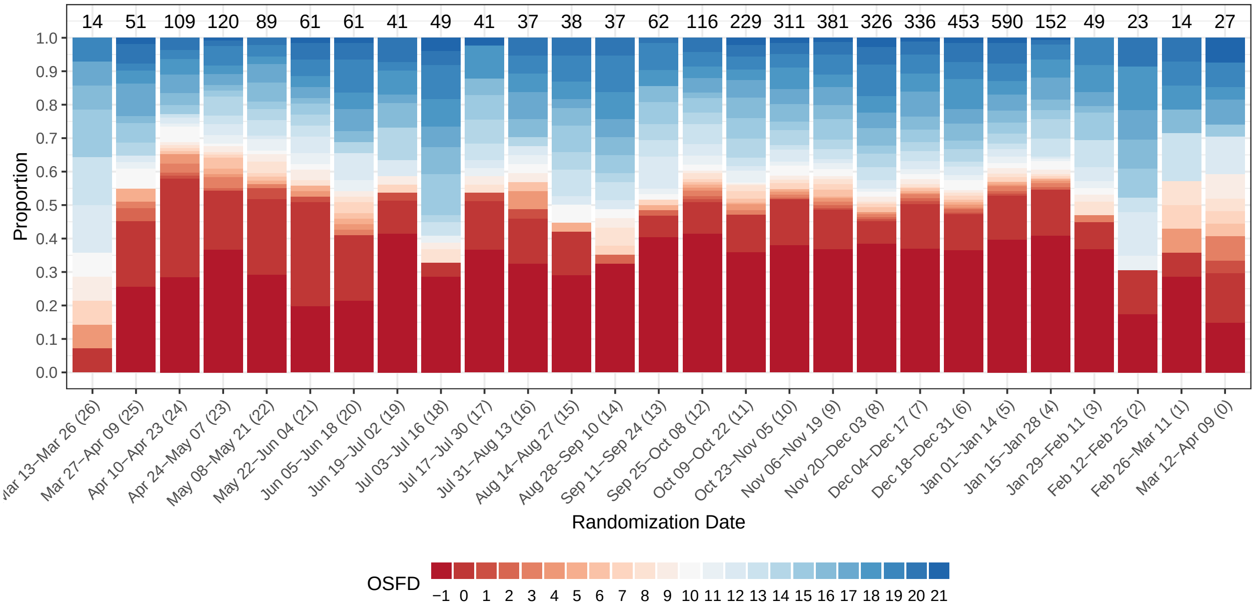

Figure 1: Empirical distribution of organ support free days (OSFD) by time epoch. The Mar 12-Apr 9 category (epoch 0) is the reference time epoch in all models. This plot shows the time epochs defined for the Unblinded ITT population, with the labeled epoch number shown in parentheses.

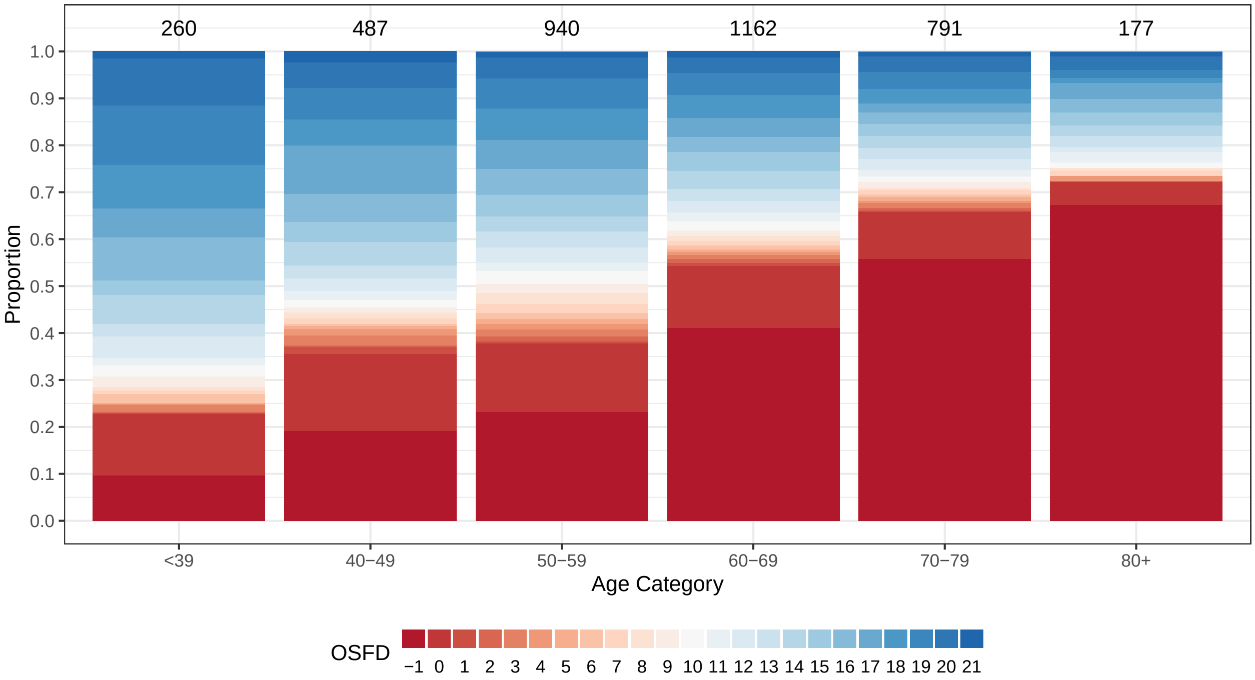

Figure 2: Empirical distribution of organ support free days (OSFD) by age category for the Unblinded ITT population. The 60—69 year age category is the reference group in all models.

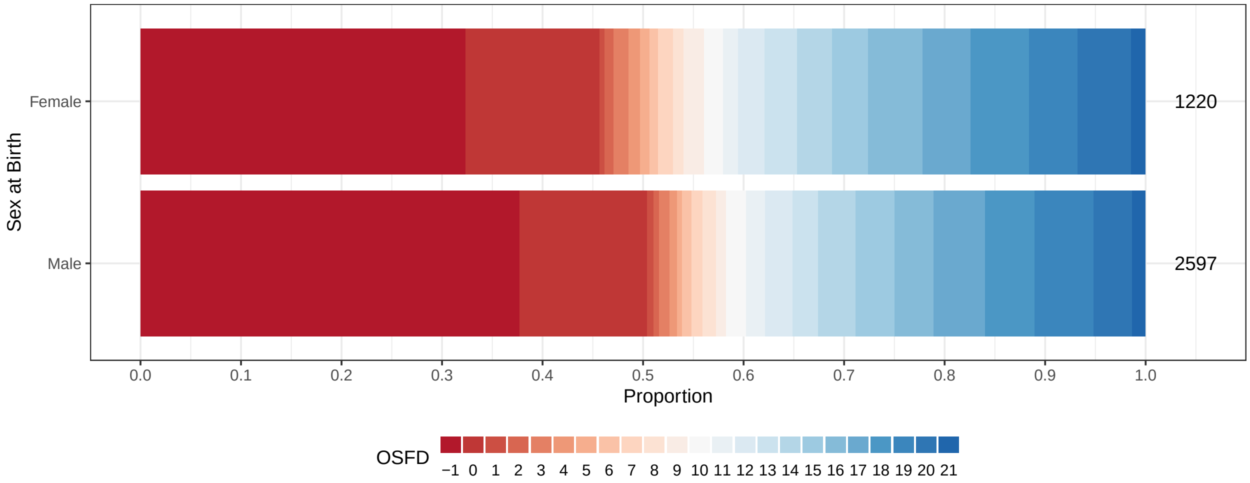

Figure 3: Empirical distribution of organ support free days (OSFD) by sex at birth for the Unblinded ITT population. The male category is the reference group in all models.

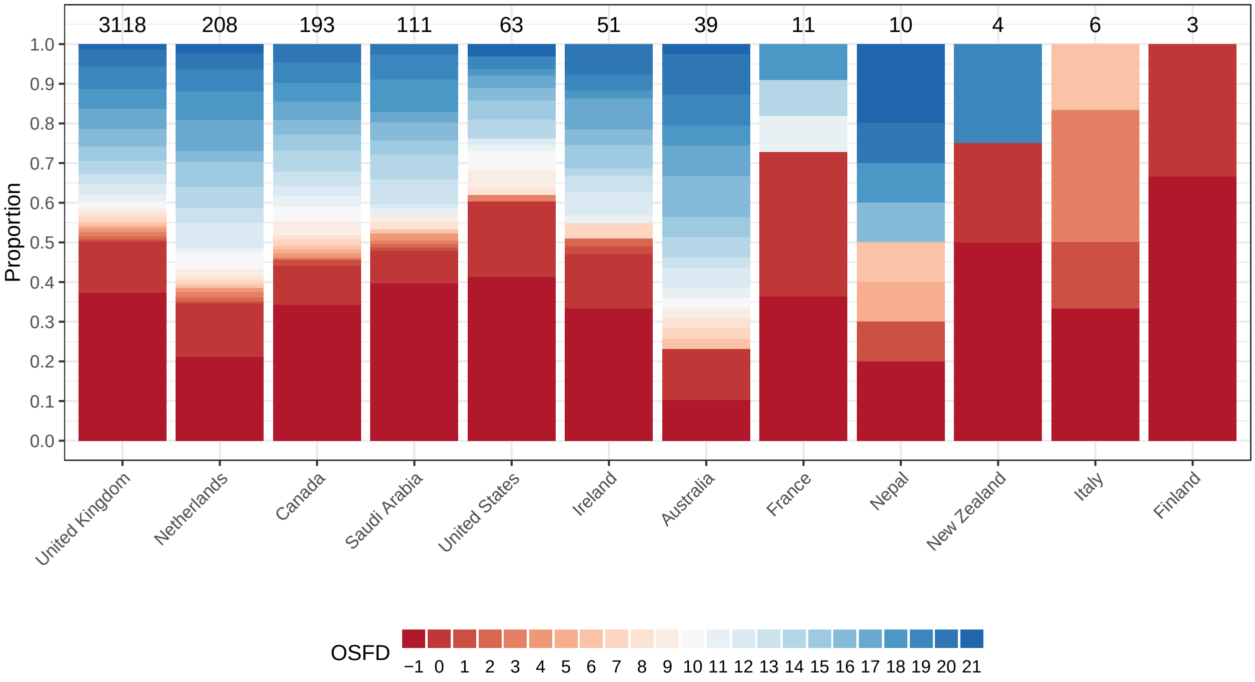

Figure 4: Empirical distribution of organ support free days (OSFD) by country for the Unblinded ITT population.

2 Secondary analyses of OSFD

2.1 Empirical distribution of OSFD in Immune Modulation ITT population

Table 1: Summary of OSFD for the Immune Modulation ITT population

Intervention	# Patients	# Known	In-hospital deaths	OSFD median (IQR)	OSFD in survivors* median (IQR)
Interferon-beta-1a	19	19	7 (36.8%)	0 (-1, 13.5)	11 (0, 17.25)
Anakinra	373	365	145 (39.7%)	0 (-1, 15)	14 (3.75, 18)
Tocilizumab	947	938	317 (33.8%)	7 (-1, 16)	15 (7, 18)
Sarilumab	482	480	157 (32.7%)	9 (-1, 17)	15 (9, 18)
Control	405	405	150 (37%)	0 (-1, 15)	13 (4, 17)
Pooled IL-6ra	1429	1418	474 (33.4%)	8 (-1, 17)	15 (8, 18)

* Days free of organ support in survivors within 21 days

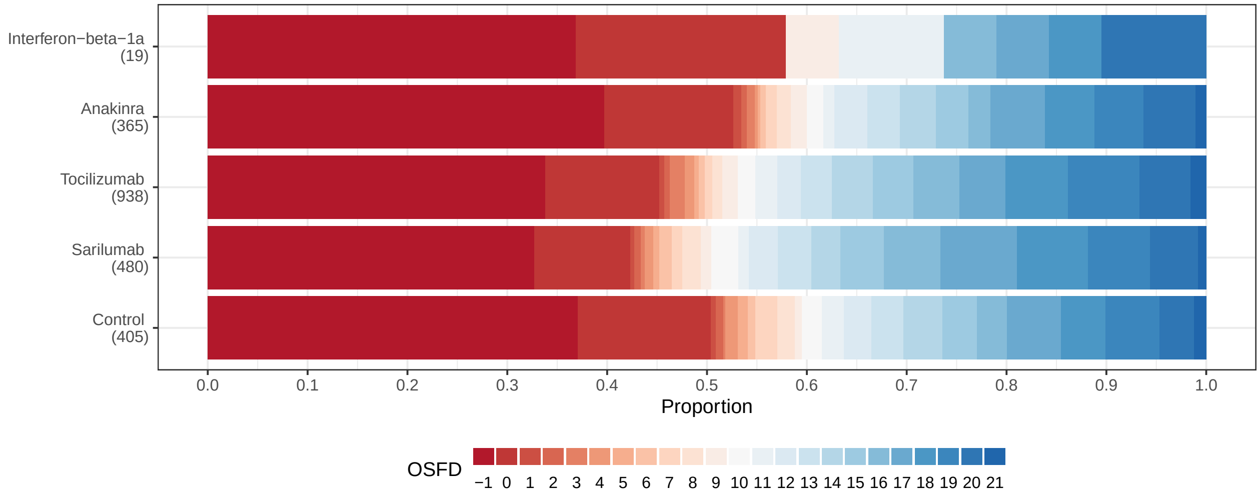

Figure 5: Empirical distribution of organ support free days (OSFD) for interferon-beta-1a, anakinra, tocilizumab, sarilumab, and control. This plot is restricted to the Immune Modulation ITT population.

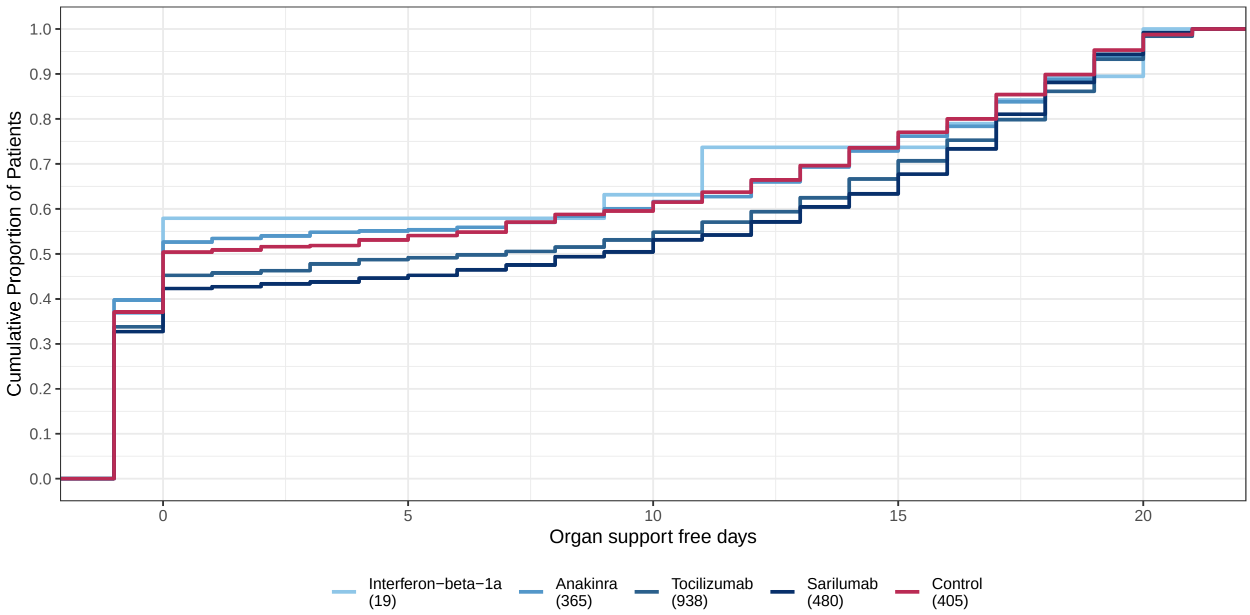

Figure 6: Empirical cumulative distribution of organ support free days (OSFD) for interferon-beta-1a, anakinra, tocilizumab, sarilumab, and control. This plot is restricted to the Immune Modulation ITT population.

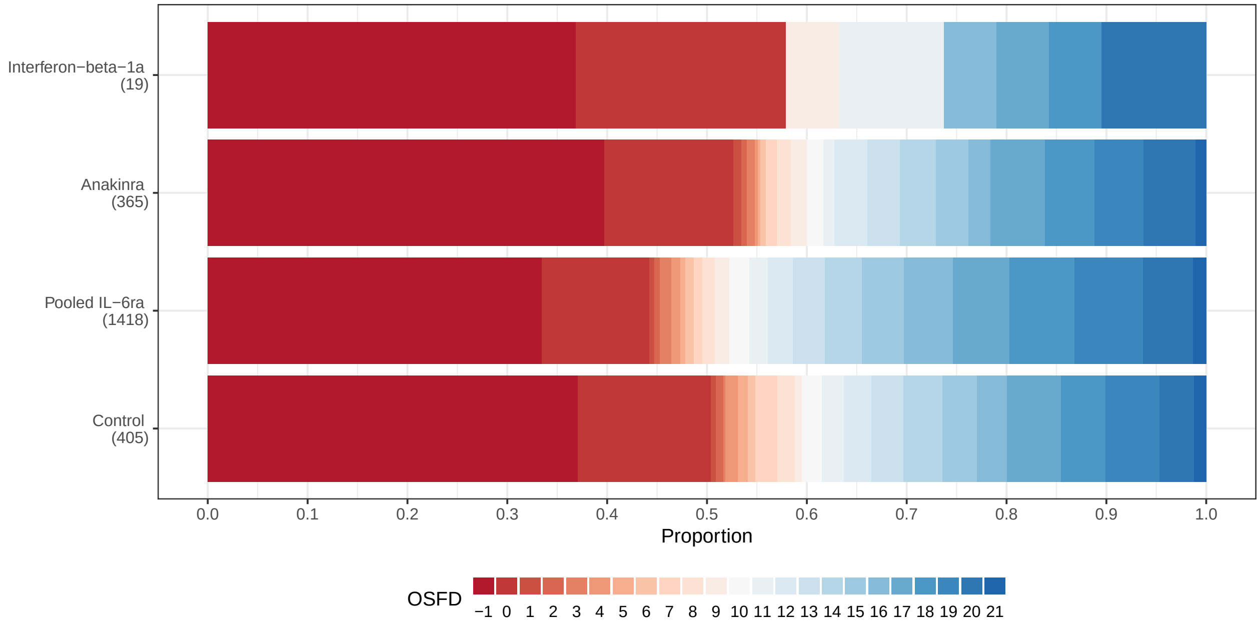

Figure 7: Empirical cumulative distribution of organ support free days (OSFD) for Immune Modulation interventions and control. This plot is restricted to the Immune Modulation ITT population.

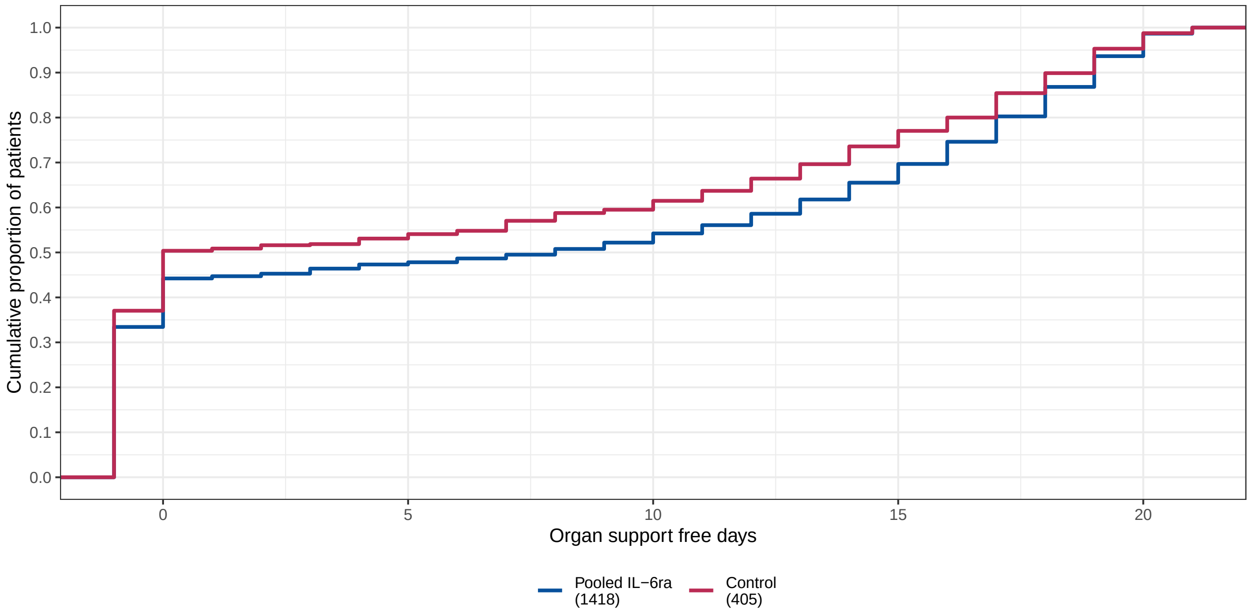

Figure 8: Empirical cumulative distribution of organ support free days (OSFD) for Immune Modulation and control interventions. This plot is restricted to the Immune Modulation ITT population.

2.2 Summary of OSFD odds ratios for IL-6ra interventions

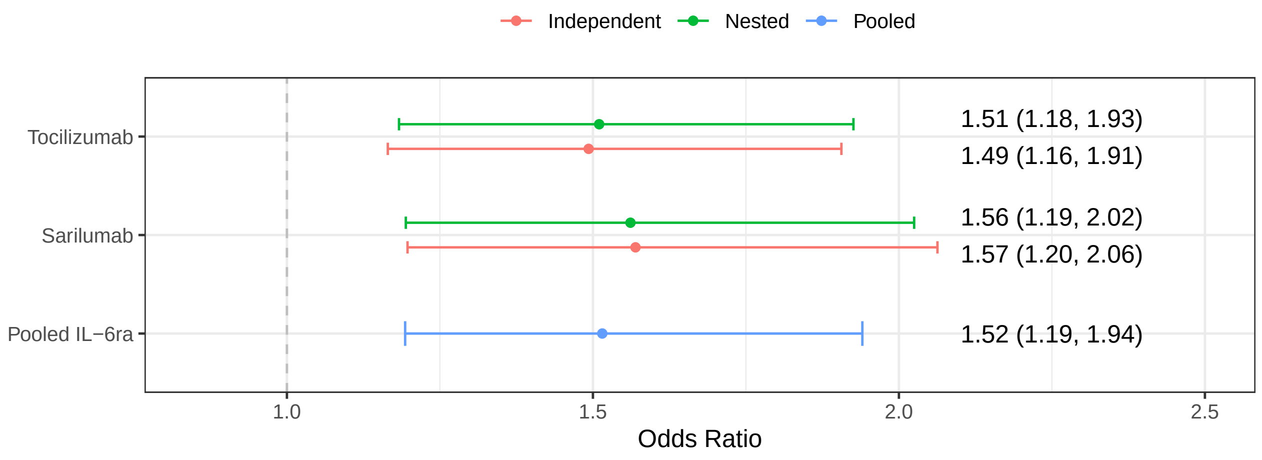

Figure 9: OSFD odds ratios for tocilizumab, sarilumab, and pooled IL-6ra in models with nested, independent, and pooled treatment effects.

2.3 Secondary analysis of OSFD for Unblinded ITT population with nesting between IL-6ra intervention effects

- Model: Primary analysis ordinal model
- Factors: Age, sex, site, time, Immune Modulation Therapy Domain interventions (control, interferon-beta-1a, anakinra, tocilizumab, and sarilumab), COVID-19 Antiviral domain interventions (no antiviral, lopinavir−ritonavir, hydroxychloroquine, combination therapy), Corticosteroid domain interventions (no corticosteroid, fixed-duration corticosteroids, shock-dependent corticosteroids), Immunoglobulin domain interventions (no convalescent plasma, convalescent plasma, delayed convalescent plasma), and Anticoagulation interventions (usual care thromboprophylaxis, therapeutic anticoagulation)
- Treatment effects for tocilizumab and sarilumab are nested with a hierarchical prior

Table 2: Odds ratio parameters for secondary analysis of OSFD for Unblinded ITT population with nested tocilizumab/sarilumab effects

	Mean	SD	Median	CrI
Interferon-beta-1a	1.37	0.56	1.27	(0.58, 2.75)
Anakinra	1.07	0.17	1.06	(0.77, 1.44)
Tocilizumab	1.52	0.19	1.51	(1.18, 1.93)
Sarilumab	1.57	0.21	1.56	(1.19, 2.02)
Lopinavir−ritonavir	0.80	0.12	0.79	(0.60, 1.05)
Hydroxychloroquine	0.61	0.13	0.61	(0.37, 0.88)
Fixed-dose corticosteroids	1.46	0.32	1.42	(0.93, 2.19)
Shock-dependent corticosteroids	1.16	0.26	1.13	(0.74, 1.76)
Therapeutic anticogulation	0.86	0.10	0.86	(0.68, 1.08)
Convalescent plasma	0.95	0.08	0.95	(0.80, 1.11)
Delayed convalescent plasma	0.54	0.30	0.47	(0.16, 1.30)
Lopinavir−ritonavir*Tocilizumab combination	1.23	0.24	1.21	(0.83, 1.76)
Hydroxychloroquine*Tocilizumab combination	0.93	0.24	0.91	(0.54, 1.45)
Tocilizumab*Fixed-dose corticosteroids combination	2.21	0.56	2.14	(1.31, 3.49)
Lopinavir−ritonavir*Tocilizumab interaction	1.01	0.05	1.01	(0.92, 1.11)
Tocilizumab*Fixed-dose corticosteroids interaction	1.00	0.05	1.00	(0.91, 1.10)
Hydroxychloroquine*Tocilizumab interaction	1.00	0.05	1.00	(0.91, 1.09)
Age<39	4.30	0.53	4.27	(3.36, 5.46)
Age 40-49	2.38	0.23	2.37	(1.96, 2.85)
Age 50-59	1.85	0.15	1.84	(1.58, 2.15)
Age 70-79	0.52	0.05	0.52	(0.43, 0.62)
Age 80+	0.31	0.05	0.30	(0.21, 0.42)
Female	1.15	0.07	1.15	(1.01, 1.30)

Table 3: Posterior probabilities for secondary analysis of OSFD for Unblinded ITT population with nested tocilizumab/sarilumab effects

	Posterior Probability
Interferon-beta-1a is optimal	0.283
Interferon-beta-1a is superior to control	0.728
Interferon-beta-1a is futile (OR < 1.2)	0.443
Interferon-beta-1a is harmful (OR < 1)	0.273
Anakinra is optimal	0.000
Anakinra is superior to control	0.647
Anakinra is futile (OR < 1.2)	0.786
Anakinra is harmful (OR < 1)	0.353
Tocilizumab is optimal	0.248
Tocilizumab is superior to control	1.000
Tocilizumab is futile (OR < 1.2)	0.032
Tocilizumab is harmful (OR < 1)	0.001
Sarilumab is optimal	0.469
Sarilumab is superior to control	0.999
Sarilumab is futile (OR < 1.2)	0.028
Sarilumab is harmful (OR < 1)	0.001
Lopinavir−ritonavir*tocilizumab combination OR > 1	0.837
Hydroxychloroquine*tocilizumab combination OR > 1	0.358
Fixed-dose steroids*tocilizumab combination OR > 1	0.998
Interferon-beta-1a equivalent to anakinra	0.314
Interferon-beta-1a equivalent to tocilizumab	0.324
Interferon-beta-1a equivalent to sarilumab	0.307
Anakinra equivalent to tocilizumab	0.094
Anakinra equivalent to sarilumab	0.062
Tocilizumab equivalent to sarilumab	0.929
Anakinra superior to tocilizumab	0.004
Anakinra superior to sarilumab	0.002
Tocilizumab superior to sarilumab	0.361

2.4 Secondary analysis of OSFD for Unblinded ITT population with independent IL-6ra intervention effects

- Model: Primary analysis ordinal model
- Factors: Age, sex, site, time, Immune Modulation Therapy Domain interventions (control, interferon-beta-1a, anakinra, tocilizumab, and sarilumab), COVID-19 Antiviral domain interventions (no antiviral, lopinavir−ritonavir, hydroxychloroquine, combination therapy), Corticosteroid domain interventions (no corticosteroid, fixed-duration corticosteroids, shock-dependent corticosteroids), Immunoglobulin

domain interventions (no convalescent plasma, convalescent plasma, delayed convalescent plasma), and Anticoagulation interventions (usual care thromboprophylaxis, therapeutic anticoagulation)

- Treatment effects for tocilizumab and sarilumab are modeled with independent priors.

Table 4: Odds ratio parameters for secondary analysis of OSFD for Unblinded ITT population with independent tocilizumab/sarilumab effects

	Mean	SD	Median	CrI
Interferon-beta-1a	1.36	0.55	1.26	(0.58, 2.65)
Anakinra	1.07	0.17	1.06	(0.78, 1.44)
Tocilizumab	1.50	0.19	1.49	(1.16, 1.91)
Sarilumab	1.59	0.22	1.57	(1.20, 2.06)
Lopinavir−ritonavir	0.80	0.12	0.79	(0.59, 1.06)
Hydroxychloroquine	0.61	0.14	0.61	(0.37, 0.89)
Fixed-dose corticosteroids	1.46	0.32	1.42	(0.93, 2.18)
Shock-dependent corticosteroids	1.16	0.26	1.13	(0.73, 1.76)
Therapeutic anticogulation	0.86	0.10	0.85	(0.68, 1.07)
Convalescent plasma	0.95	0.08	0.95	(0.80, 1.11)
Delayed convalescent plasma	0.54	0.32	0.47	(0.16, 1.35)
Lopinavir−ritonavir*Tocilizumab combination	1.21	0.24	1.19	(0.80, 1.74)
Hydroxychloroquine*Tocilizumab combination	0.92	0.24	0.90	(0.52, 1.45)
Tocilizumab*Fixed-dose corticosteroids combination	2.19	0.56	2.12	(1.29, 3.44)
Lopinavir−ritonavir*Tocilizumab interaction	1.01	0.05	1.01	(0.92, 1.11)
Tocilizumab*Fixed-dose corticosteroids interaction	1.00	0.05	1.00	(0.91, 1.10)
Hydroxychloroquine*Tocilizumab interaction	1.00	0.05	1.00	(0.91, 1.10)
Age<39	4.30	0.53	4.27	(3.34, 5.43)
Age 40-49	2.38	0.23	2.37	(1.96, 2.86)
Age 50-59	1.85	0.15	1.84	(1.57, 2.15)
Age 70-79	0.52	0.05	0.52	(0.43, 0.62)
Age 80+	0.31	0.05	0.30	(0.21, 0.42)
Female	1.15	0.07	1.15	(1.01, 1.30)

Table 5: Posterior probabilities for secondary analysis of OSFD for Unblinded ITT population with independent tocilizumab/sarilumab effects

	Posterior Probability
Interferon-beta-1a is optimal	0.272
Interferon-beta-1a is superior to control	0.719
Interferon-beta-1a is futile (OR < 1.2)	0.453
Interferon-beta-1a is harmful (OR < 1)	0.281
Anakinra is optimal	0.001
Anakinra is superior to control	0.646
Anakinra is futile (OR < 1.2)	0.787
Anakinra is harmful (OR < 1)	0.354
Tocilizumab is optimal	0.222
Tocilizumab is superior to control	0.999
Tocilizumab is futile (OR < 1.2)	0.043
Tocilizumab is harmful (OR < 1)	0.001
Sarilumab is optimal	0.506
Sarilumab is superior to control	0.999
Sarilumab is futile (OR < 1.2)	0.026
Sarilumab is harmful (OR < 1)	0.001
Lopinavir−ritonavir*tocilizumab combination OR > 1	0.810
Hydroxychloroquine*tocilizumab combination OR > 1	0.342
Fixed-dose steroids*tocilizumab combination OR > 1	0.999
Interferon-beta-1a equivalent to anakinra	0.311
Interferon-beta-1a equivalent to tocilizumab	0.325
Interferon-beta-1a equivalent to sarilumab	0.301
Anakinra equivalent to tocilizumab	0.114
Anakinra equivalent to sarilumab	0.063
Tocilizumab equivalent to sarilumab	0.837
Anakinra superior to tocilizumab	0.005
Anakinra superior to sarilumab	0.002
Tocilizumab superior to sarilumab	0.333

2.5 Secondary analysis of OSFD for Unblinded ITT population with pooled IL-6ra effect

- Model: Primary analysis ordinal model
- Factors: Age, sex, site, time, Immune Modulation Therapy Domain interventions (control, interferon-beta-1a, anakinra, tocilizumab, and sarilumab), COVID-19 Antiviral domain interventions (no antiviral, lopinavir−ritonavir, hydroxychloroquine, combination therapy), Corticosteroid domain interventions (no corticosteroid, fixed-duration corticosteroids, shock-dependent corticosteroids), Immunoglobulin domain interventions (no convalescent plasma, convalescent plasma, delayed convalescent plasma),

and Anticoagulation interventions (usual care thromboprophylaxis, therapeutic anticoagulation)

- Treatment effects for tocilizumab and sarilumab are pooled into a single IL-6ra effect.

Table 6: Odds ratio parameters for secondary analysis of OSFD for Unblinded ITT population with pooled tocilizumab/sarilumab effects

	Mean	SD	Median	CrI
Interferon-beta-1a	1.36	0.56	1.26	(0.58, 2.69)
Anakinra	1.07	0.17	1.05	(0.78, 1.45)
Pooled IL-6ra	1.53	0.19	1.52	(1.19, 1.94)
Lopinavir−ritonavir	0.80	0.12	0.79	(0.60, 1.05)
Hydroxychloroquine	0.61	0.13	0.61	(0.37, 0.89)
Fixed-dose corticosteroids	1.46	0.32	1.42	(0.94, 2.16)
Shock-dependent corticosteroids	1.16	0.26	1.13	(0.74, 1.74)
Therapeutic anticogulation	0.86	0.10	0.86	(0.68, 1.07)
Convalescent plasma	0.95	0.08	0.94	(0.80, 1.11)
Delayed convalescent plasma	0.54	0.31	0.47	(0.16, 1.34)
Lopinavir−ritonavir*Pooled IL-6ra combination	1.23	0.24	1.21	(0.83, 1.77)
Hydroxychloroquine*Pooled IL-6ra combination	0.94	0.24	0.92	(0.53, 1.47)
Pooled IL-6ra*Fixed-dose corticosteroids combination	2.22	0.56	2.16	(1.32, 3.50)
Lopinavir−ritonavir*Pooled IL-6ra interaction	1.01	0.05	1.01	(0.92, 1.11)
Pooled IL-6ra*Fixed-dose corticosteroids interaction	1.00	0.05	1.00	(0.91, 1.11)
Hydroxychloroquine*Pooled IL-6ra interaction	1.00	0.05	1.00	(0.91, 1.11)
Age<39	4.31	0.52	4.29	(3.38, 5.40)
Age 40-49	2.38	0.23	2.37	(1.97, 2.86)
Age 50-59	1.85	0.15	1.84	(1.58, 2.15)
Age 70-79	0.52	0.05	0.52	(0.43, 0.62)
Age 80+	0.31	0.05	0.30	(0.21, 0.42)
Female	1.15	0.07	1.15	(1.02, 1.30)

Table 7: Posterior probabilities for secondary analysis of OSFD for Unblinded ITT population with pooled tocilizumab/sarilumab effects

	Posterior Probability
Interferon-beta-1a is optimal	0.317
Interferon-beta-1a is superior to control	0.724
Interferon-beta-1a is futile (OR < 1.2)	0.452
Interferon-beta-1a is harmful (OR < 1)	0.277
Anakinra is optimal	0.001
Anakinra is superior to control	0.639
Anakinra is futile (OR < 1.2)	0.794
Anakinra is harmful (OR < 1)	0.361
Pooled IL-6ra is optimal	0.682
Pooled IL-6ra is superior to control	1.000
Pooled IL-6ra is futile (OR < 1.2)	0.028
Pooled IL-6ra is harmful (OR < 1)	0.000
Lopinavir−ritonavir*Pooled IL-6ra combination OR > 1	0.837
Hydroxychloroquine*Pooled IL-6ra combination OR > 1	0.366
Fixed-dose steroids*Pooled IL-6ra combination OR > 1	0.999
Interferon-beta-1a equivalent to anakinra	0.320
Interferon-beta-1a equivalent to pooled IL-6ra	0.327
Anakinra equivalent to pooled IL-6ra	0.083
Anakinra superior to pooled IL-6ra	0.003

2.6 Secondary analysis of OSFD for Unblinded ITT population with additional unblinded interaction effects

- Model: Primary analysis ordinal model
- Factors: Age, sex, site, time, Immune Modulation Therapy Domain interventions (control, interferon-beta-1a, anakinra, tocilizumab, and sarilumab), COVID-19 Antiviral domain interventions (no antiviral, lopinavir−ritonavir, hydroxychloroquine, combination therapy), Corticosteroid domain interventions (no corticosteroid, fixed-duration corticosteroids, shock-dependent corticosteroids), Immunoglobulin domain interventions (no convalescent plasma, convalescent plasma, delayed convalescent plasma), and Anticoagulation interventions (usual care thromboprophylaxis, therapeutic anticoagulation)
- Treatment effects for tocilizumab and sarilumab are pooled into a single IL-6ra effect.

Table 8: Number of patients randomized in the Immune Modulation and Immunoglobulin domains

	No convalescent plasma	Convalescent plasma
Interferon-beta-1a	7	4
Anakinra	39	52
Tocilizumab	208	252
Sarilumab	119	143
Control	95	108

Table 9: Number of patients randomized in the Immune Modulation and Antiviral domains

	No antiviral	Lopinavir-ritonavir	HCQ	Combination therapy
Interferon-beta-1a	2	5	0	0
Anakinra	6	11	1	0
Tocilizumab	91	63	10	5
Sarilumab	16	10	0	0
Control	115	86	17	3

Table 10: Number of patients randomized in the Immune Modulation and Anticoagulation domains

	Venous thromboprophylaxis	Therapeutic dose anticoagulation
Interferon-beta-1a	3	7
Anakinra	18	22
Tocilizumab	80	90
Sarilumab	54	40
Control	71	77

Table 11: Number of patients randomized in the Immune Modulation and Corticosteroid domains. Note that this includes patients randomized after the closure of the steroid domain in the fixed-dose corticosteroids intervention

	No corticosteroid	Fixed-dose corticosteroids	Shock-dependent corticosteroids
Interferon-beta-1a	1	15	1
Anakinra	0	372	1
Tocilizumab	16	891	19
Sarilumab	0	482	0
Control	9	350	18

Table 12: Odds ratio parameters for secondary analysis of OSFD with additional unblinded interaction effects

	Mean	SD	Median	CrI
Interferon-beta-1a	1.35	0.55	1.25	(0.56, 2.68)
Anakinra	1.08	0.19	1.07	(0.76, 1.49)
Pooled IL-6ra	1.45	0.40	1.39	(0.82, 2.38)
Lopinavir−ritonavir	0.75	0.12	0.74	(0.55, 1.01)
Hydroxychloroquine	0.59	0.14	0.58	(0.35, 0.87)
Fixed-dose corticosteroids	1.45	0.32	1.41	(0.91, 2.18)
Shock-dependent corticosteroids	1.15	0.26	1.13	(0.73, 1.75)
Therapeutic anticogulation	0.88	0.11	0.87	(0.68, 1.12)
Convalescent plasma	0.92	0.10	0.92	(0.75, 1.12)
Delayed convalescent plasma	0.54	0.32	0.47	(0.16, 1.36)
Lopinavir−ritonavir*Pooled IL-6ra combination	1.46	0.50	1.37	(0.71, 2.63)
Hydroxychloroquine*Pooled IL-6ra combination	1.03	0.50	0.93	(0.38, 2.26)
Pooled IL-6ra*Fixed-dose corticosteroids combination	2.08	0.55	2.02	(1.21, 3.31)
Pooled IL-6ra*Therapeutic anticoagulation combination	1.20	0.39	1.14	(0.61, 2.16)
Pooled IL-6ra*Convalescent plasma combination	1.50	0.45	1.44	(0.81, 2.57)
Anakinra*Convalescent plasma combination	0.78	0.24	0.75	(0.42, 1.33)
Lopinavir−ritonavir*Pooled IL-6ra interaction	1.38	0.36	1.33	(0.80, 2.20)
Pooled IL-6ra*Fixed-dose corticosteroids interaction	1.31	0.66	1.17	(0.46, 2.96)
Hydroxychloroquine*Pooled IL-6ra interaction	1.06	0.28	1.02	(0.61, 1.70)
Pooled IL-6ra*Therapeutic anticoagulation interaction	0.96	0.19	0.94	(0.64, 1.40)
Pooled IL-6ra*Convalescent plasma interaction	1.14	0.17	1.12	(0.84, 1.50)
Anakinra*Convalescent plasma interaction	0.80	0.25	0.76	(0.42, 1.38)
Age<39	4.29	0.52	4.26	(3.37, 5.40)
Age 40-49	2.39	0.23	2.38	(1.97, 2.88)
Age 50-59	1.85	0.15	1.84	(1.57, 2.16)
Age 70-79	0.52	0.05	0.52	(0.43, 0.62)
Age 80+	0.30	0.05	0.30	(0.21, 0.42)
Female	1.15	0.07	1.15	(1.02, 1.31)

Table 13: Posterior probabilities for secondary analysis of OSFD for Unblinded ITT population with additional unblinded interaction effects

	Posterior Probability
Interferon-beta-1a is optimal	0.212
Interferon-beta-1a is superior to control	0.715
Interferon-beta-1a is futile (OR < 1.2)	0.458
Interferon-beta-1a is harmful (OR < 1)	0.285
Anakinra is optimal	0.006
Anakinra is superior to control	0.649
Anakinra is futile (OR < 1.2)	0.749
Anakinra is harmful (OR < 1)	0.351
Pooled IL-6ra is optimal	0.782
Pooled IL-6ra is superior to control	0.889
Pooled IL-6ra is futile (OR < 1.2)	0.290
Pooled IL-6ra is harmful (OR < 1)	0.111
Lopinavir−ritonavir*Pooled IL-6ra combination OR > 1	0.829
Hydroxychloroquine*Pooled IL-6ra combination OR > 1	0.438
Fixed-dose steroids*Pooled IL-6ra combination OR > 1	0.996
Therapeutic anticoagulation*Pooled IL-6ra combination OR > 1	0.664
Convalescent plasma*Pooled IL-6ra combination OR > 1	0.894
Convalescent plasma*Anakinra OR > 1	0.161
Interferon-beta-1a equivalent to anakinra	0.315
Interferon-beta-1a equivalent to pooled IL-6ra	0.301
Anakinra equivalent to pooled IL-6ra	0.326
Anakinra superior to pooled IL-6ra	0.176

2.7 Secondary analysis of OSFD for Unblinded ITT population restricted to non-negative COVID-19

- Model: Primary analysis ordinal model
- Factors: Age, sex, site, time, Immune Modulation Therapy Domain interventions (control, interferon-beta-1a, anakinra, tocilizumab, and sarilumab), COVID-19 Antiviral domain interventions (no antiviral, lopinavir−ritonavir, hydroxychloroquine, combination therapy), Corticosteroid domain interventions (no corticosteroid, fixed-duration corticosteroids, shock-dependent corticosteroids), Immunoglobulin domain interventions (no convalescent plasma, convalescent plasma, delayed convalescent plasma), and Anticoagulation interventions (usual care thromboprophylaxis, therapeutic anticoagulation)
- Treatment effects for tocilizumab and sarilumab are modeled with independent priors.

Table 14: Odds ratio parameters for secondary analysis of OSFD for the non-negative population

	Mean	SD	Median	CrI
Interferon-beta-1a	1.08	0.50	0.99	(0.41, 2.31)
Anakinra	1.11	0.18	1.10	(0.80, 1.51)
Tocilizumab	1.55	0.20	1.53	(1.18, 1.99)
Sarilumab	1.69	0.25	1.68	(1.26, 2.23)
Lopinavir−ritonavir	0.87	0.14	0.86	(0.63, 1.18)
Hydroxychloroquine	0.58	0.15	0.57	(0.33, 0.89)
Fixed-dose corticosteroids	1.35	0.32	1.32	(0.83, 2.10)
Shock-dependent corticosteroids	1.02	0.25	0.99	(0.62, 1.61)
Therapeutic anticogulation	0.83	0.10	0.82	(0.65, 1.05)
Convalescent plasma	0.95	0.08	0.95	(0.81, 1.12)
Delayed convalescent plasma	0.59	0.34	0.51	(0.17, 1.45)
Lopinavir−ritonavir*Tocilizumab combination	1.36	0.29	1.33	(0.88, 2.02)
Hydroxychloroquine*Tocilizumab combination	0.89	0.26	0.87	(0.48, 1.46)
Tocilizumab*Fixed-dose corticosteroids combination	2.09	0.57	2.01	(1.21, 3.42)
Lopinavir−ritonavir*Tocilizumab interaction	1.01	0.05	1.01	(0.92, 1.11)
Tocilizumab*Fixed-dose corticosteroids interaction	1.00	0.05	1.00	(0.91, 1.10)
Hydroxychloroquine*Tocilizumab interaction	1.00	0.05	1.00	(0.91, 1.10)
Age<39	4.61	0.59	4.57	(3.56, 5.87)
Age 40-49	2.47	0.24	2.46	(2.02, 2.98)
Age 50-59	1.88	0.15	1.87	(1.60, 2.19)
Age 70-79	0.52	0.05	0.52	(0.43, 0.63)
Age 80+	0.28	0.05	0.27	(0.19, 0.39)
Female	1.16	0.08	1.16	(1.02, 1.32)

Table 15: Posterior probabilities for secondary analysis of OSFD for the non-negative population

	Posterior Probability
Interferon-beta-1a is optimal	0.104
Interferon-beta-1a is superior to control	0.493
Interferon-beta-1a is futile (OR < 1.2)	0.670
Interferon-beta-1a is harmful (OR < 1)	0.507
Anakinra is optimal	0.000
Anakinra is superior to control	0.721
Anakinra is futile (OR < 1.2)	0.712
Anakinra is harmful (OR < 1)	0.279
Tocilizumab is optimal	0.202
Tocilizumab is superior to control	0.999
Tocilizumab is futile (OR < 1.2)	0.031
Tocilizumab is harmful (OR < 1)	0.001
Sarilumab is optimal	0.693
Sarilumab is superior to control	1.000
Sarilumab is futile (OR < 1.2)	0.010
Sarilumab is harmful (OR < 1)	0.000
Lopinavir−ritonavir*tocilizumab combination OR > 1	0.913
Hydroxychloroquine*tocilizumab combination OR > 1	0.312
Fixed-dose steroids*tocilizumab combination OR > 1	0.996
Interferon-beta-1a equivalent to anakinra	0.310
Interferon-beta-1a equivalent to tocilizumab	0.203
Interferon-beta-1a equivalent to sarilumab	0.160
Anakinra equivalent to tocilizumab	0.128
Anakinra equivalent to sarilumab	0.042
Tocilizumab equivalent to sarilumab	0.767
Anakinra superior to tocilizumab	0.006
Anakinra superior to sarilumab	0.001
Tocilizumab superior to sarilumab	0.234

2.8 Secondary analysis of OSFD for Immune Modulation ITT population

- Model: Primary analysis ordinal model
- Factors: Age, sex, site, time, Immune Modulation Therapy Domain interventions (control, interferon-beta-1a, anakinra, tocilizumab, and sarilumab)
- Treatment effects for tocilizumab and sarilumab are modeled with independent priors.

Table 16: Odds ratio parameters for secondary analysis of OSFD for Immune Modulation ITT population with independent tocilizumab/sarilumab effects

	Mean	SD	Median	CrI
Interferon-beta-1a	1.29	0.53	1.20	(0.55, 2.59)
Anakinra	1.16	0.19	1.14	(0.83, 1.57)
Tocilizumab	1.55	0.19	1.54	(1.22, 1.95)
Sarilumab	1.67	0.25	1.65	(1.23, 2.22)
Age<39	4.59	0.75	4.53	(3.30, 6.20)
Age 40-49	2.53	0.33	2.50	(1.95, 3.24)
Age 50-59	2.19	0.23	2.17	(1.78, 2.66)
Age 70-79	0.56	0.07	0.56	(0.44, 0.71)
Age 80+	0.31	0.07	0.30	(0.19, 0.47)
Female	1.27	0.11	1.26	(1.07, 1.49)

Table 17: Posterior probabilities for secondary analysis of OSFD for Immune Modulation ITT population with independent tocilizumab/sarilumab effects

	Posterior Probability
Interferon-beta-1a is optimal	0.195
Interferon-beta-1a is superior to control	0.683
Interferon-beta-1a is futile (OR < 1.2)	0.497
Interferon-beta-1a is harmful (OR < 1)	0.317
Anakinra is optimal	0.001
Anakinra is superior to control	0.796
Anakinra is futile (OR < 1.2)	0.622
Anakinra is harmful (OR < 1)	0.204
Tocilizumab is optimal	0.204
Tocilizumab is superior to control	1.000
Tocilizumab is futile (OR < 1.2)	0.018
Tocilizumab is harmful (OR < 1)	0.000
Sarilumab is optimal	0.600
Sarilumab is superior to control	1.000
Sarilumab is futile (OR < 1.2)	0.017
Sarilumab is harmful (OR < 1)	0.000
Interferon-beta-1a equivalent to anakinra	0.357
Interferon-beta-1a equivalent to tocilizumab	0.300
Interferon-beta-1a equivalent to sarilumab	0.263
Anakinra equivalent to tocilizumab	0.177
Anakinra equivalent to sarilumab	0.091
Tocilizumab equivalent to sarilumab	0.839
Anakinra superior to tocilizumab	0.008
Anakinra superior to sarilumab	0.004
Tocilizumab superior to sarilumab	0.268

2.9 Sensitivity analysis of OSFD for Unblinded ITT population with site and time factors removed

- Model: Primary analysis ordinal model
- Factors: Age, sex, Immune Modulation Therapy Domain interventions (control, interferon-beta-1a, anakinra, tocilizumab, and sarilumab), COVID-19 Antiviral domain interventions (no antiviral, lopinavir–ritonavir, hydroxychloroquine, combination therapy), Corticosteroid domain interventions (no corticosteroid, fixed-duration corticosteroids, shock-dependent corticosteroids), Immunoglobulin domain interventions (no convalescent plasma, convalescent plasma, delayed convalescent plasma), and Anticoagulation interventions (usual care thromboprophylaxis, therapeutic anticoag-

ulation) corticosteroids, and shock-dependent steroids and reported interventions of the Immune Modulation Therapy Domain: tocilizumab, sarilumab (combined to a single IL-6 arm) and no immune modulation

- Treatment effects for tocilizumab and sarilumab are modeled with independent priors.

Table 18: Odds ratio parameters for the sensitivity analysis of OSFD with site and time removed

	Mean	SD	Median	CrI
Interferon-beta-1a	1.12	0.46	1.03	(0.48, 2.23)
Anakinra	0.99	0.14	0.98	(0.74, 1.30)
Tocilizumab	1.42	0.17	1.41	(1.12, 1.79)
Sarilumab	1.39	0.18	1.37	(1.07, 1.77)
Lopinavir−ritonavir	0.82	0.12	0.81	(0.61, 1.08)
Hydroxychloroquine	0.53	0.12	0.52	(0.32, 0.79)
Fixed-dose corticosteroids	1.32	0.23	1.30	(0.92, 1.83)
Shock-dependent corticosteroids	1.20	0.26	1.17	(0.78, 1.79)
Therapeutic anticogulation	0.89	0.10	0.88	(0.70, 1.10)
Convalescent plasma	0.96	0.08	0.96	(0.82, 1.12)
Delayed convalescent plasma	0.55	0.29	0.49	(0.18, 1.29)
Lopinavir−ritonavir*Tocilizumab combination	1.17	0.23	1.14	(0.79, 1.67)
Hydroxychloroquine*Tocilizumab combination	0.75	0.20	0.73	(0.43, 1.20)
Tocilizumab*Fixed-dose corticosteroids combination	1.87	0.39	1.84	(1.24, 2.77)
Lopinavir−ritonavir*Tocilizumab interaction	1.01	0.05	1.01	(0.92, 1.11)
Tocilizumab*Fixed-dose corticosteroids interaction	1.00	0.05	1.00	(0.91, 1.11)
Hydroxychloroquine*Tocilizumab interaction	1.00	0.05	1.00	(0.91, 1.10)
Age<39	3.79	0.46	3.76	(2.97, 4.76)
Age 40-49	2.24	0.21	2.22	(1.85, 2.68)
Age 50-59	1.80	0.14	1.80	(1.54, 2.09)
Age 70-79	0.57	0.05	0.57	(0.48, 0.68)
Age 80+	0.37	0.06	0.36	(0.26, 0.50)
Female	1.16	0.07	1.16	(1.03, 1.31)

Table 19: Posterior probabilities for the sensitivity analysis of OSFD with site and time removed

	Posterior Probability
Interferon-beta-1a is optimal	0.198
Interferon-beta-1a is superior to control	0.536
Interferon-beta-1a is futile (OR < 1.2)	0.642
Interferon-beta-1a is harmful (OR < 1)	0.464
Anakinra is optimal	0.000
Anakinra is superior to control	0.436
Anakinra is futile (OR < 1.2)	0.926
Anakinra is harmful (OR < 1)	0.564
Tocilizumab is optimal	0.485
Tocilizumab is superior to control	0.998
Tocilizumab is futile (OR < 1.2)	0.087
Tocilizumab is harmful (OR < 1)	0.002
Sarilumab is optimal	0.317
Sarilumab is superior to control	0.992
Sarilumab is futile (OR < 1.2)	0.143
Sarilumab is harmful (OR < 1)	0.008
Lopinavir−ritonavir*tocilizumab combination OR > 1	0.758
Hydroxychloroquine*tocilizumab combination OR > 1	0.116
Fixed-dose steroids*tocilizumab combination OR > 1	1.000
Interferon-beta-1a equivalent to anakinra	0.338
Interferon-beta-1a equivalent to tocilizumab	0.266
Interferon-beta-1a equivalent to sarilumab	0.283
Anakinra equivalent to tocilizumab	0.074
Anakinra equivalent to sarilumab	0.120
Tocilizumab equivalent to sarilumab	0.892
Anakinra superior to tocilizumab	0.002
Anakinra superior to sarilumab	0.004
Tocilizumab superior to sarilumab	0.593

3 Secondary analyses of in-hospital mortality

All models in this report are parameterized so that an OR/HR greater than 1 indicates patient benefit relative to the reference group and an OR/HR less than 1 indicates patient harm relative to the reference group. In this section, the ORs are summarized for the outcome of “hospital survival” which is defined as the complement of in-hospital survival.

3.1 Empirical distribution of in-hospital mortality in Immune Modulation ITT population

Table 20: Summary of in-hospital mortality for the Immune Modulation ITT population

Intervention	# Patients (N)	# Known (n)	Deaths (y)	Mortality Rate (y/n)
Interferon-beta-1a	19	19	7	0.368
Anakinra	373	365	145	0.397
Tocilizumab	947	938	317	0.338
Sarilumab	482	480	157	0.327
Control	405	405	150	0.370
Pooled IL-6ra	1429	1418	474	0.334

3.2 Summary of hospital survival odds ratios for IL-6ra interventions

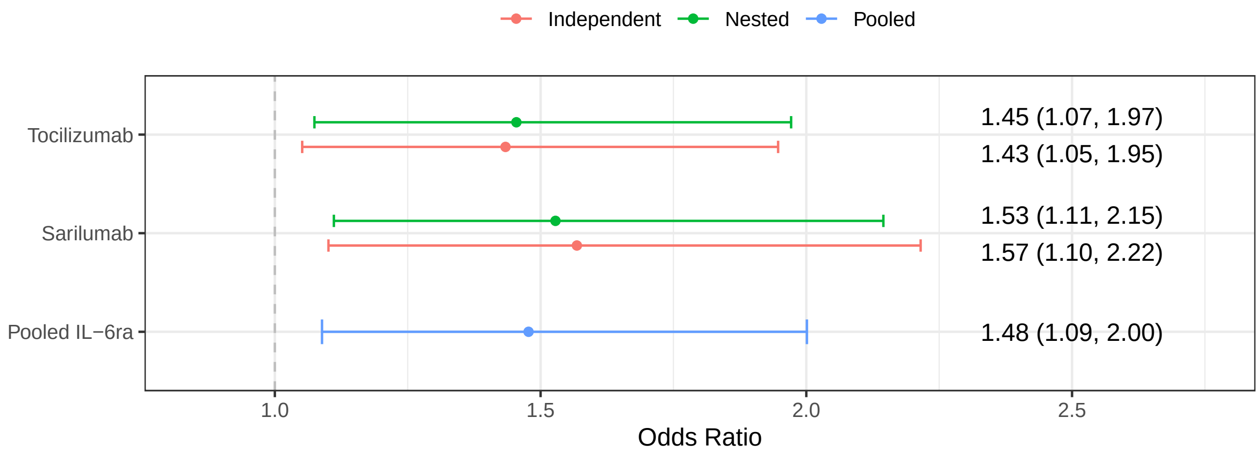

Figure 10: Hospital survival odds ratios for tocilizumab, sarilumab, and pooled IL-6ra in models with nested, independent, and pooled treatment effects.

3.3 Secondary analysis of hospital survival for Unblinded ITT population with nesting between IL-6ra intervention effects

- Model: Primary dichotomous model
- Factors: Age, sex, site, time, Immune Modulation Therapy Domain interventions (control, interferon-beta-1a, anakinra, tocilizumab, and sarilumab), COVID-19 Antiviral domain interventions (no antiviral, lopinavir–ritonavir, hydroxychloroquine, combination therapy), Corticosteroid domain interventions (no corticosteroid, fixed-duration corticosteroids, shock-dependent corticosteroids), Immunoglobulin

domain interventions (no convalescent plasma, convalescent plasma, delayed convalescent plasma), and Anticoagulation interventions (usual care thromboprophylaxis, therapeutic anticoagulation)

- Treatment effects for tocilizumab and sarilumab are nested with a hierarchical prior

Table 21: Odds ratio parameters for secondary analysis of hospital survival for Unblinded ITT population with nested tocilizumab/sarilumab effects

	Mean	SD	Median	CrI
Interferon-beta-1a	1.42	0.72	1.26	(0.52, 3.22)
Anakinra	1.03	0.20	1.01	(0.70, 1.49)
Tocilizumab	1.47	0.23	1.45	(1.07, 1.97)
Sarilumab	1.55	0.27	1.53	(1.11, 2.15)
Lopinavir−ritonavir	0.72	0.13	0.70	(0.50, 1.02)
Hydroxychloroquine	0.57	0.15	0.56	(0.30, 0.88)
Fixed-dose corticosteroids	1.02	0.29	0.98	(0.58, 1.68)
Shock-dependent corticosteroids	1.19	0.36	1.14	(0.65, 2.04)
Therapeutic anticogulation	0.83	0.12	0.82	(0.62, 1.09)
Convalescent plasma	1.02	0.11	1.01	(0.82, 1.25)
Delayed convalescent plasma	0.84	0.58	0.69	(0.20, 2.35)
Lopinavir−ritonavir*Tocilizumab combination	1.06	0.26	1.03	(0.65, 1.65)
Hydroxychloroquine*Tocilizumab combination	0.84	0.27	0.81	(0.40, 1.43)
Tocilizumab*Fixed-dose corticosteroids combination	1.48	0.48	1.40	(0.77, 2.62)
Lopinavir−ritonavir*Tocilizumab interaction	1.01	0.05	1.00	(0.91, 1.10)
Tocilizumab*Fixed-dose corticosteroids interaction	1.01	0.05	1.00	(0.91, 1.11)
Hydroxychloroquine*Tocilizumab interaction	0.99	0.05	0.99	(0.90, 1.09)
Age<39	8.54	1.95	8.31	(5.44, 13.07)
Age 40-49	3.48	0.47	3.45	(2.65, 4.52)
Age 50-59	2.45	0.26	2.44	(1.99, 2.99)
Age 70-79	0.47	0.05	0.47	(0.38, 0.57)
Age 80+	0.26	0.05	0.26	(0.18, 0.37)
Female	1.25	0.10	1.25	(1.06, 1.47)

Table 22: Posterior probabilities for secondary analysis of hospital survival for Unblinded ITT population with nested tocilizumab/sarilumab effects

	Posterior Probability
Interferon-beta-1a is optimal	0.317
Interferon-beta-1a is superior to control	0.689
Interferon-beta-1a is futile (OR < 1.2)	0.460
Interferon-beta-1a is harmful (OR < 1)	0.311
Anakinra is optimal	0.001
Anakinra is superior to control	0.524
Anakinra is futile (OR < 1.2)	0.813
Anakinra is harmful (OR < 1)	0.476
Tocilizumab is optimal	0.212
Tocilizumab is superior to control	0.993
Tocilizumab is futile (OR < 1.2)	0.103
Tocilizumab is harmful (OR < 1)	0.007
Sarilumab is optimal	0.469
Sarilumab is superior to control	0.996
Sarilumab is futile (OR < 1.2)	0.072
Sarilumab is harmful (OR < 1)	0.004
Lopinavir−ritonavir*tocilizumab combination OR > 1	0.549
Hydroxychloroquine*tocilizumab combination OR > 1	0.250
Fixed-dose steroids*tocilizumab combination OR > 1	0.862
Interferon-beta-1a equivalent to anakinra	0.272
Interferon-beta-1a equivalent to tocilizumab	0.290
Interferon-beta-1a equivalent to sarilumab	0.279
Anakinra equivalent to tocilizumab	0.126
Anakinra equivalent to sarilumab	0.076
Tocilizumab equivalent to sarilumab	0.858
Anakinra superior to tocilizumab	0.011
Anakinra superior to sarilumab	0.006
Tocilizumab superior to sarilumab	0.326

3.4 Secondary analysis of hospital survival for Unblinded ITT population with independent IL-6ra intervention effects

- Model: Primary dichotomous model
- Factors: Age, sex, site, time, Immune Modulation Therapy Domain interventions (control, interferon-beta-1a, anakinra, tocilizumab, and sarilumab), COVID-19 Antiviral domain interventions (no antiviral, lopinavir−ritonavir, hydroxychloroquine, combination therapy), Corticosteroid domain interventions (no corticosteroid, fixed-duration corticosteroids, shock-dependent corticosteroids), Immunoglobulin

domain interventions (no convalescent plasma, convalescent plasma, delayed convalescent plasma), and Anticoagulation interventions (usual care thromboprophylaxis, therapeutic anticoagulation)

- Treatment effects for tocilizumab and sarilumab are modeled with independent priors.

Table 23: Odds ratio parameters for secondary analysis of hospital survival for Unblinded ITT population with independent tocilizumab/sarilumab effects

	Mean	SD	Median	CrI
Interferon-beta-1a	1.42	0.72	1.26	(0.51, 3.27)
Anakinra	1.03	0.20	1.01	(0.70, 1.49)
Tocilizumab	1.45	0.23	1.43	(1.05, 1.95)
Sarilumab	1.59	0.29	1.57	(1.10, 2.22)
Lopinavir−ritonavir	0.72	0.13	0.70	(0.50, 1.02)
Hydroxychloroquine	0.57	0.15	0.56	(0.30, 0.89)
Fixed-dose corticosteroids	1.02	0.30	0.98	(0.55, 1.69)
Shock-dependent corticosteroids	1.20	0.38	1.14	(0.62, 2.08)
Therapeutic anticogulation	0.83	0.12	0.82	(0.62, 1.08)
Convalescent plasma	1.02	0.11	1.01	(0.83, 1.24)
Delayed convalescent plasma	0.84	0.58	0.69	(0.21, 2.41)
Lopinavir−ritonavir*Tocilizumab combination	1.05	0.26	1.02	(0.63, 1.64)
Hydroxychloroquine*Tocilizumab combination	0.83	0.26	0.80	(0.40, 1.41)
Tocilizumab*Fixed-dose corticosteroids combination	1.47	0.49	1.40	(0.73, 2.62)
Lopinavir−ritonavir*Tocilizumab interaction	1.01	0.05	1.00	(0.91, 1.10)
Tocilizumab*Fixed-dose corticosteroids interaction	1.01	0.05	1.00	(0.91, 1.11)
Hydroxychloroquine*Tocilizumab interaction	0.99	0.05	0.99	(0.90, 1.09)
Age<39	8.56	1.94	8.33	(5.49, 13.02)
Age 40-49	3.49	0.48	3.46	(2.67, 4.53)
Age 50-59	2.46	0.25	2.45	(1.99, 2.98)
Age 70-79	0.47	0.05	0.47	(0.38, 0.57)
Age 80+	0.26	0.05	0.26	(0.18, 0.37)
Female	1.25	0.11	1.25	(1.06, 1.47)

Table 24: Posterior probabilities for secondary analysis of hospital survival for Unblinded ITT population with independent tocilizumab/sarilumab effects

	Posterior Probability
Interferon-beta-1a is optimal	0.306
Interferon-beta-1a is superior to control	0.691
Interferon-beta-1a is futile (OR < 1.2)	0.456
Interferon-beta-1a is harmful (OR < 1)	0.309
Anakinra is optimal	0.001
Anakinra is superior to control	0.529
Anakinra is futile (OR < 1.2)	0.809
Anakinra is harmful (OR < 1)	0.470
Tocilizumab is optimal	0.176
Tocilizumab is superior to control	0.990
Tocilizumab is futile (OR < 1.2)	0.131
Tocilizumab is harmful (OR < 1)	0.011
Sarilumab is optimal	0.516
Sarilumab is superior to control	0.993
Sarilumab is futile (OR < 1.2)	0.069
Sarilumab is harmful (OR < 1)	0.007
Lopinavir−ritonavir*tocilizumab combination OR > 1	0.523
Hydroxychloroquine*tocilizumab combination OR > 1	0.232
Fixed-dose steroids*tocilizumab combination OR > 1	0.850
Interferon-beta-1a equivalent to anakinra	0.263
Interferon-beta-1a equivalent to tocilizumab	0.286
Interferon-beta-1a equivalent to sarilumab	0.271
Anakinra equivalent to tocilizumab	0.148
Anakinra equivalent to sarilumab	0.069
Tocilizumab equivalent to sarilumab	0.701
Anakinra superior to tocilizumab	0.015
Anakinra superior to sarilumab	0.005
Tocilizumab superior to sarilumab	0.272

3.5 Secondary analysis of hospital survival for Unblinded ITT population with pooled IL-6ra effect

- Model: Primary dichotomous model
- Factors: Age, sex, site, time, Immune Modulation Therapy Domain interventions (control, interferon-beta-1a, anakinra, tocilizumab, and sarilumab), COVID-19 Antiviral domain interventions (no antiviral, lopinavir−ritonavir, hydroxychloroquine, combination therapy), Corticosteroid domain interventions (no corticosteroid, fixed-duration corticosteroids, shock-dependent corticosteroids), Immunoglobulin

domain interventions (no convalescent plasma, convalescent plasma, delayed convalescent plasma), and Anticoagulation interventions (usual care thromboprophylaxis, therapeutic anticoagulation)

- Treatment effects for tocilizumab and sarilumab are pooled into a single IL-6ra effect.

Table 25: Odds ratio parameters for secondary analysis of hospital survival for Unblinded ITT population with pooled tocilizumab/sarilumab effects

	Mean	SD	Median	CrI
Interferon-beta-1a	1.42	0.72	1.27	(0.51, 3.25)
Anakinra	1.03	0.20	1.01	(0.69, 1.47)
Pooled IL-6ra	1.49	0.23	1.48	(1.09, 2.00)
Lopinavir−ritonavir	0.72	0.13	0.70	(0.50, 1.01)
Hydroxychloroquine	0.56	0.15	0.55	(0.30, 0.89)
Fixed-dose corticosteroids	1.03	0.30	0.98	(0.57, 1.72)
Shock-dependent corticosteroids	1.20	0.38	1.15	(0.64, 2.10)
Therapeutic anticogulation	0.83	0.12	0.82	(0.61, 1.09)
Convalescent plasma	1.02	0.11	1.01	(0.83, 1.24)
Delayed convalescent plasma	0.84	0.58	0.69	(0.20, 2.32)
Lopinavir−ritonavir*Pooled IL-6ra combination	1.08	0.27	1.04	(0.66, 1.68)
Hydroxychloroquine*Pooled IL-6ra combination	0.85	0.27	0.82	(0.42, 1.44)
Pooled IL-6ra*Fixed-dose corticosteroids combination	1.52	0.51	1.44	(0.76, 2.74)
Lopinavir−ritonavir*Pooled IL-6ra interaction	1.01	0.05	1.00	(0.91, 1.11)
Pooled IL-6ra*Fixed-dose corticosteroids interaction	1.01	0.05	1.00	(0.91, 1.11)
Hydroxychloroquine*Pooled IL-6ra interaction	0.99	0.05	0.99	(0.90, 1.09)
Age<39	8.53	1.95	8.29	(5.47, 12.98)
Age 40-49	3.49	0.48	3.46	(2.64, 4.53)
Age 50-59	2.45	0.25	2.44	(2.00, 2.97)
Age 70-79	0.47	0.05	0.47	(0.38, 0.57)
Age 80+	0.27	0.05	0.26	(0.18, 0.37)
Female	1.25	0.10	1.25	(1.07, 1.47)

Table 26: Posterior probabilities for secondary analysis of hospital survival for Unblinded ITT population with pooled tocilizumab/sarilumab effects

	Posterior Probability
Interferon-beta-1a is optimal	0.377
Interferon-beta-1a is superior to control	0.692
Interferon-beta-1a is futile (OR < 1.2)	0.452
Interferon-beta-1a is harmful (OR < 1)	0.308
Anakinra is optimal	0.003
Anakinra is superior to control	0.516
Anakinra is futile (OR < 1.2)	0.821
Anakinra is harmful (OR < 1)	0.484
Pooled IL-6ra is optimal	0.618
Pooled IL-6ra is superior to control	0.994
Pooled IL-6ra is futile (OR < 1.2)	0.089
Pooled IL-6ra is harmful (OR < 1)	0.006
Lopinavir−ritonavir*Pooled IL-6ra combination OR > 1	0.571
Hydroxychloroquine*Pooled IL-6ra combination OR > 1	0.257
Fixed-dose steroids*Pooled IL-6ra combination OR > 1	0.874
Interferon-beta-1a equivalent to anakinra	0.267
Interferon-beta-1a equivalent to pooled IL-6ra	0.277
Anakinra equivalent to Pooled IL-6ra	0.092
Anakinra superior to Pooled IL-6ra	0.006

3.6 Secondary analysis of hospital survival for Unblinded ITT population with additional unblinded interaction effects

- Model: Primary dichotomous model
- Factors: Age, sex, site, time, Immune Modulation Therapy Domain interventions (control, interferon-beta-1a, anakinra, tocilizumab, and sarilumab), COVID-19 Antiviral domain interventions (no antiviral, lopinavir−ritonavir, hydroxychloroquine, combination therapy), Corticosteroid domain interventions (no corticosteroid, fixed-duration corticosteroids, shock-dependent corticosteroids), Immunoglobulin domain interventions (no convalescent plasma, convalescent plasma, delayed convalescent plasma), and Anticoagulation interventions (usual care thromboprophylaxis, therapeutic anticoagulation)
- Treatment effects for tocilizumab and sarilumab are pooled into a single IL-6ra effect.

Table 27: Odds ratio parameters for secondary analysis of hospital survival with additional unblinded interaction effects

	Mean	SD	Median	CrI
Interferon-beta-1a	1.41	0.72	1.26	(0.50, 3.26)
Anakinra	1.05	0.23	1.02	(0.68, 1.56)
Pooled IL-6ra	2.11	0.90	1.93	(0.90, 4.35)
Lopinavir−ritonavir	0.70	0.14	0.68	(0.47, 1.03)
Hydroxychloroquine	0.53	0.15	0.52	(0.26, 0.85)
Fixed-dose corticosteroids	1.09	0.32	1.04	(0.60, 1.83)
Shock-dependent corticosteroids	1.21	0.37	1.15	(0.64, 2.09)
Therapeutic anticogulation	0.84	0.13	0.83	(0.61, 1.13)
Convalescent plasma	0.97	0.12	0.96	(0.75, 1.23)
Delayed convalescent plasma	0.83	0.55	0.70	(0.21, 2.26)
Lopinavir−ritonavir*Pooled IL-6ra combination	1.71	0.87	1.53	(0.61, 3.83)
Hydroxychloroquine*Pooled IL-6ra combination	2.19	1.82	1.68	(0.45, 6.99)
Pooled IL-6ra*Fixed-dose corticosteroids combination	1.45	0.49	1.37	(0.73, 2.64)
Pooled IL-6ra*Therapeutic anticoagulation combination	1.72	0.84	1.55	(0.64, 3.81)
Pooled IL-6ra*Convalescent plasma combination	2.49	1.12	2.28	(0.99, 5.31)
Anakinra*Convalescent plasma combination	0.73	0.24	0.69	(0.36, 1.30)
Lopinavir−ritonavir*Pooled IL-6ra interaction	1.21	0.39	1.16	(0.62, 2.12)
Pooled IL-6ra*Fixed-dose corticosteroids interaction	2.19	1.68	1.73	(0.49, 6.61)
Hydroxychloroquine*Pooled IL-6ra interaction	0.73	0.30	0.68	(0.31, 1.45)
Pooled IL-6ra*Therapeutic anticoagulation interaction	1.00	0.26	0.96	(0.59, 1.60)
Pooled IL-6ra*Convalescent plasma interaction	1.23	0.23	1.21	(0.85, 1.74)
Anakinra*Convalescent plasma interaction	0.74	0.26	0.70	(0.36, 1.39)
Age<39	8.57	1.96	8.32	(5.47, 13.09)
Age 40-49	3.52	0.48	3.49	(2.68, 4.55)
Age 50-59	2.47	0.26	2.45	(2.01, 3.01)
Age 70-79	0.47	0.05	0.47	(0.38, 0.57)
Age 80+	0.27	0.05	0.26	(0.18, 0.37)
Female	1.25	0.10	1.25	(1.06, 1.47)

Table 28: Posterior probabilities for secondary analysis of hospital survival for Unblinded ITT population with additional unblinded interaction effects

	Posterior Probability
Interferon-beta-1a is optimal	0.109
Interferon-beta-1a is superior to control	0.686
Interferon-beta-1a is futile (OR < 1.2)	0.460
Interferon-beta-1a is harmful (OR < 1)	0.314
Anakinra is optimal	0.003
Anakinra is superior to control	0.539
Anakinra is futile (OR < 1.2)	0.772
Anakinra is harmful (OR < 1)	0.461
Pooled IL-6ra is optimal	0.886
Pooled IL-6ra is superior to control	0.954
Pooled IL-6ra is futile (OR < 1.2)	0.113
Pooled IL-6ra is harmful (OR < 1)	0.046
Lopinavir−ritonavir*Pooled IL-6ra combination OR > 1	0.820
Hydroxychloroquine*Pooled IL-6ra combination OR > 1	0.780
Fixed-dose steroids*Pooled IL-6ra combination OR > 1	0.842
Therapeutic anticoagulation*Pooled IL-6ra combination OR > 1	0.830
Convalescent plasma*Pooled IL-6ra combination OR > 1	0.974
Convalescent plasma*Anakinra OR > 1	0.127
Interferon-beta-1a equivalent to anakinra	0.266
Interferon-beta-1a equivalent to pooled IL-6ra	0.185
Anakinra equivalent to pooled IL-6ra	0.114
Anakinra superior to pooled IL-6ra	0.061

3.7 Secondary analysis of hospital survival for Unblinded ITT population restricted to non-negative COVID-19

- Model: Primary dichotomous model
- Factors: Age, sex, site, time, Immune Modulation Therapy Domain interventions (control, interferon-beta-1a, anakinra, tocilizumab, and sarilumab), COVID-19 Antiviral domain interventions (no antiviral, lopinavir−ritonavir, hydroxychloroquine, combination therapy), Corticosteroid domain interventions (no corticosteroid, fixed-duration corticosteroids, shock-dependent corticosteroids), Immunoglobulin domain interventions (no convalescent plasma, convalescent plasma, delayed convalescent plasma), and Anticoagulation interventions (usual care thromboprophylaxis, therapeutic anticoagulation)
- Treatment effects for tocilizumab and sarilumab are modeled with independent priors.

Table 29: Odds ratio parameters for secondary analysis of hospital survival for the non-negative population

	Mean	SD	Median	CrI
Interferon-beta-1a	1.04	0.58	0.90	(0.33, 2.51)
Anakinra	1.06	0.21	1.04	(0.70, 1.52)
Tocilizumab	1.48	0.24	1.46	(1.06, 2.00)
Sarilumab	1.68	0.31	1.65	(1.15, 2.37)
Lopinavir−ritonavir	0.75	0.15	0.73	(0.50, 1.10)
Hydroxychloroquine	0.49	0.16	0.48	(0.23, 0.82)
Fixed-dose corticosteroids	0.94	0.29	0.90	(0.48, 1.62)
Shock-dependent corticosteroids	1.07	0.36	1.01	(0.53, 1.92)
Therapeutic anticogulation	0.81	0.12	0.80	(0.60, 1.07)
Convalescent plasma	1.02	0.11	1.01	(0.83, 1.24)
Delayed convalescent plasma	0.92	0.65	0.76	(0.22, 2.57)
Lopinavir−ritonavir*Tocilizumab combination	1.12	0.30	1.08	(0.65, 1.82)
Hydroxychloroquine*Tocilizumab combination	0.73	0.27	0.69	(0.32, 1.34)
Tocilizumab*Fixed-dose corticosteroids combination	1.37	0.49	1.29	(0.65, 2.54)
Lopinavir−ritonavir*Tocilizumab interaction	1.01	0.05	1.00	(0.91, 1.11)
Tocilizumab*Fixed-dose corticosteroids interaction	1.01	0.05	1.00	(0.91, 1.11)
Hydroxychloroquine*Tocilizumab interaction	0.99	0.05	0.99	(0.90, 1.09)
Age<39	8.80	2.05	8.51	(5.60, 13.47)
Age 40-49	3.54	0.50	3.50	(2.67, 4.61)
Age 50-59	2.44	0.26	2.42	(1.97, 2.98)
Age 70-79	0.48	0.05	0.48	(0.39, 0.59)
Age 80+	0.26	0.05	0.26	(0.17, 0.37)
Female	1.26	0.11	1.25	(1.06, 1.48)

Table 30: Posterior probabilities for secondary analysis of hospital survival for the non-negative population

	Posterior Probability
Interferon-beta-1a is optimal	0.116
Interferon-beta-1a is superior to control	0.421
Interferon-beta-1a is futile (OR < 1.2)	0.712
Interferon-beta-1a is harmful (OR < 1)	0.579
Anakinra is optimal	0.001
Anakinra is superior to control	0.576
Anakinra is futile (OR < 1.2)	0.780
Anakinra is harmful (OR < 1)	0.424
Tocilizumab is optimal	0.169
Tocilizumab is superior to control	0.990
Tocilizumab is futile (OR < 1.2)	0.110
Tocilizumab is harmful (OR < 1)	0.010
Sarilumab is optimal	0.713
Sarilumab is superior to control	0.998
Sarilumab is futile (OR < 1.2)	0.040
Sarilumab is harmful (OR < 1)	0.002
Lopinavir−ritonavir*tocilizumab combination OR > 1	0.616
Hydroxychloroquine*tocilizumab combination OR > 1	0.150
Fixed-dose steroids*tocilizumab combination OR > 1	0.767
Interferon-beta-1a equivalent to anakinra	0.268
Interferon-beta-1a equivalent to tocilizumab	0.183
Interferon-beta-1a equivalent to sarilumab	0.141
Anakinra equivalent to tocilizumab	0.156
Anakinra equivalent to sarilumab	0.045
Tocilizumab equivalent to sarilumab	0.630
Anakinra superior to tocilizumab	0.018
Anakinra superior to sarilumab	0.003
Tocilizumab superior to sarilumab	0.201

3.8 Secondary analysis of hospital survival for Immune Modulation ITT population

- Model: Primary dichotomous model
- Factors: Age, sex, site, time, Immune Modulation Therapy Domain interventions (control, interferon-beta-1a, anakinra, tocilizumab, and sarilumab)
- Treatment effects for tocilizumab and sarilumab are modeled with independent priors.

Table 31: Odds ratio parameters for secondary analysis of hospital survival for Immune Modulation ITT population with independent tocilizumab/sarilumab effects

	Mean	SD	Median	CrI
Interferon-beta-1a	1.42	0.71	1.27	(0.51, 3.19)
Anakinra	1.14	0.23	1.12	(0.76, 1.66)
Tocilizumab	1.55	0.25	1.53	(1.13, 2.09)
Sarilumab	1.78	0.34	1.74	(1.21, 2.54)
Age<39	9.07	2.83	8.61	(4.96, 15.83)
Age 40-49	3.50	0.64	3.43	(2.43, 4.94)
Age 50-59	2.86	0.38	2.83	(2.17, 3.68)
Age 70-79	0.54	0.07	0.53	(0.41, 0.69)
Age 80+	0.26	0.06	0.25	(0.16, 0.41)
Female	1.44	0.16	1.43	(1.16, 1.78)

Table 32: Posterior probabilities for secondary analysis of hospital survival for Immune Modulation ITT population with independent tocilizumab/sarilumab effects

	Posterior Probability
Interferon-beta-1a is optimal	0.244
Interferon-beta-1a is superior to control	0.694
Interferon-beta-1a is futile (OR < 1.2)	0.452
Interferon-beta-1a is harmful (OR < 1)	0.306
Anakinra is optimal	0.001
Anakinra is superior to control	0.709
Anakinra is futile (OR < 1.2)	0.635
Anakinra is harmful (OR < 1)	0.291
Tocilizumab is optimal	0.127
Tocilizumab is superior to control	0.998
Tocilizumab is futile (OR < 1.2)	0.061
Tocilizumab is harmful (OR < 1)	0.003
Sarilumab is optimal	0.627
Sarilumab is superior to control	0.999
Sarilumab is futile (OR < 1.2)	0.023
Sarilumab is harmful (OR < 1)	0.001
Interferon-beta-1a equivalent to anakinra	0.280
Interferon-beta-1a equivalent to tocilizumab	0.279
Interferon-beta-1a equivalent to sarilumab	0.236
Anakinra equivalent to tocilizumab	0.193
Anakinra equivalent to sarilumab	0.059
Tocilizumab equivalent to sarilumab	0.639
Anakinra superior to tocilizumab	0.020
Anakinra superior to sarilumab	0.005
Tocilizumab superior to sarilumab	0.183

3.9 Sensitivity analysis of hospital survival for Unblinded ITT population with site and time factors removed

- Model: Primary dichotomous model
- Factors: Age, sex, Immune Modulation Therapy Domain interventions (control, interferon-beta-1a, anakinra, tocilizumab, and sarilumab), COVID-19 Antiviral domain interventions (no antiviral, lopinavir–ritonavir, hydroxychloroquine, combination therapy), Corticosteroid domain interventions (no corticosteroid, fixed-duration corticosteroids, shock-dependent corticosteroids), Immunoglobulin domain interventions (no convalescent plasma, convalescent plasma, delayed convalescent plasma), and Anticoagulation interventions (usual care thromboprophylaxis, therapeutic anticoagu-

lation)

- Treatment effects for tocilizumab and sarilumab are modeled with independent priors.

Table 33: Odds ratio parameters for the sensitivity analysis of hospital survival with site and time removed

	Mean	SD	Median	CrI
Interferon-beta-1a	1.26	0.62	1.13	(0.47, 2.87)
Anakinra	0.97	0.17	0.95	(0.68, 1.33)
Tocilizumab	1.37	0.20	1.35	(1.02, 1.80)
Sarilumab	1.37	0.22	1.35	(1.00, 1.88)
Lopinavir−ritonavir	0.73	0.13	0.72	(0.51, 1.03)
Hydroxychloroquine	0.52	0.14	0.51	(0.27, 0.82)
Fixed-dose corticosteroids	0.83	0.20	0.81	(0.51, 1.28)
Shock-dependent corticosteroids	1.35	0.40	1.30	(0.74, 2.30)
Therapeutic anticogulation	0.85	0.12	0.84	(0.64, 1.12)
Convalescent plasma	1.04	0.10	1.03	(0.85, 1.26)
Delayed convalescent plasma	0.97	0.60	0.82	(0.26, 2.54)
Lopinavir−ritonavir*Tocilizumab combination	1.00	0.24	0.97	(0.62, 1.55)
Hydroxychloroquine*Tocilizumab combination	0.72	0.22	0.69	(0.35, 1.20)
Tocilizumab*Fixed-dose corticosteroids combination	1.13	0.30	1.09	(0.65, 1.82)
Lopinavir−ritonavir*Tocilizumab interaction	1.00	0.05	1.00	(0.91, 1.10)
Tocilizumab*Fixed-dose corticosteroids interaction	1.01	0.05	1.00	(0.91, 1.11)
Hydroxychloroquine*Tocilizumab interaction	0.99	0.05	0.99	(0.90, 1.09)
Age<39	6.88	1.53	6.69	(4.45, 10.43)
Age 40-49	3.10	0.41	3.07	(2.38, 4.00)
Age 50-59	2.32	0.23	2.31	(1.91, 2.80)
Age 70-79	0.54	0.05	0.54	(0.45, 0.65)
Age 80+	0.33	0.06	0.32	(0.23, 0.45)
Female	1.27	0.10	1.26	(1.08, 1.47)

Table 34: Posterior probabilities for the sensitivity analysis of hospital survival with site and time removed

	Posterior Probability
Interferon-beta-1a is optimal	0.299
Interferon-beta-1a is superior to control	0.603
Interferon-beta-1a is futile (OR < 1.2)	0.552
Interferon-beta-1a is harmful (OR < 1)	0.397
Anakinra is optimal	0.001
Anakinra is superior to control	0.387
Anakinra is futile (OR < 1.2)	0.912
Anakinra is harmful (OR < 1)	0.613
Tocilizumab is optimal	0.342
Tocilizumab is superior to control	0.981
Tocilizumab is futile (OR < 1.2)	0.205
Tocilizumab is harmful (OR < 1)	0.019
Sarilumab is optimal	0.355
Sarilumab is superior to control	0.975
Sarilumab is futile (OR < 1.2)	0.224
Sarilumab is harmful (OR < 1)	0.025
Lopinavir−ritonavir*tocilizumab combination OR > 1	0.455
Hydroxychloroquine*tocilizumab combination OR > 1	0.111
Fixed-dose steroids*tocilizumab combination OR > 1	0.629
Interferon-beta-1a equivalent to anakinra	0.289
Interferon-beta-1a equivalent to tocilizumab	0.292
Interferon-beta-1a equivalent to sarilumab	0.288
Anakinra equivalent to tocilizumab	0.123
Anakinra equivalent to sarilumab	0.136
Tocilizumab equivalent to sarilumab	0.818
Anakinra superior to tocilizumab	0.008
Anakinra superior to sarilumab	0.011
Tocilizumab superior to sarilumab	0.495

4 Safety analyses

4.1 Empirical distribution of serious adverse events in Immune Modulation ITT population

Table 35: Summary of any serious adverse event (SAE) for the Immune Modulation ITT population. Note that this table shows the number of patients with any serious adverse event rather than the total number of serious adverse events observed.

Intervention	# Patients (N)	# Known (n)	Any serious adverse event (y)	Rate of any serious adverse event (y/n)
Interferon-beta-1a	19	19	1	0.053
Anakinra	373	373	10	0.027
Tocilizumab	947	947	24	0.025
Sarilumab	482	482	8	0.017
Control	405	405	11	0.027

4.2 Primary safety analysis of serious adverse events in Immune Modulation ITT population

- Model: Primary dichotomous model
- Factors: Age, sex, site, Immune Modulation Therapy Domain interventions (control, interferon-beta-1a, anakinra, tocilizumab, and sarilumab)
- Treatment effects for tocilizumab and sarilumab are modeled with independent priors.

Table 36: Odds ratio parameters for the safety analysis of freedom from serious adverse events. ORs above 1 indicate fewer SAEs, and ORs below 1 indicate more SAEs.

	Mean	SD	Median	CrI
Interferon-beta-1a	1.26	1.16	0.92	(0.22, 4.33)
Anakinra	1.32	0.60	1.20	(0.51, 2.77)
Tocilizumab	1.15	0.41	1.08	(0.53, 2.15)
Sarilumab	1.68	0.79	1.52	(0.65, 3.66)
Age<39	2.67	2.01	2.13	(0.72, 7.70)
Age 40-49	2.22	1.24	1.91	(0.80, 5.40)
Age 50-59	2.27	0.94	2.07	(1.02, 4.64)
Age 70-79	0.99	0.34	0.93	(0.49, 1.84)
Age 80+	1.89	1.50	1.47	(0.45, 5.70)
Female	1.00	0.31	0.96	(0.54, 1.74)

Table 37: Posterior probabilities for the primary safety analysis of freedom from serious adverse events

	Posterior Probability
Interferon-beta-1a is superior to control	0.456
Anakinra is superior to control	0.669
Tocilizumab is superior to control	0.592
Sarilumab is superior to control	0.840

5 Per protocol analyses

5.1 Data summaries for per protocol analyses

Table 38: Summary of patients treated per protocol by treatment arm

Intervention	Patients	Known	Treated per protocol
Interferon-beta-1a	19	19	14 (73.7%)
Anakinra	373	369	349 (94.6%)
Tocilizumab	947	932	901 (96.7%)
Sarilumab	482	468	449 (95.9%)
Control	405	401	394 (98.3%)
Pooled IL-6ra	1429	1400	1350 (96.4%)

Table 39: Summary of OSFD for the Immune Modulation Per Protocol population

Intervention	# Patients	# Known	In-hospital deaths	OSFD median (IQR)	OSFD in survivors* median (IQR)
Interferon-beta-1a	14	14	5 (35.7%)	0 (-1, 10.5)	9 (0, 17)
Anakinra	349	343	140 (40.8%)	0 (-1, 15)	14 (6.5, 18)
Tocilizumab	901	896	294 (32.8%)	8 (-1, 16.25)	14.5 (7.25, 18)
Sarilumab	449	447	145 (32.4%)	9 (-1, 17)	15 (8.25, 18)
Control	394	394	143 (36.3%)	0.5 (-1, 15)	13 (4, 17)
Pooled IL-6ra	1350	1343	439 (32.7%)	8 (-1, 17)	15 (8, 18)

[*] Days free of organ support in survivors within 21 days

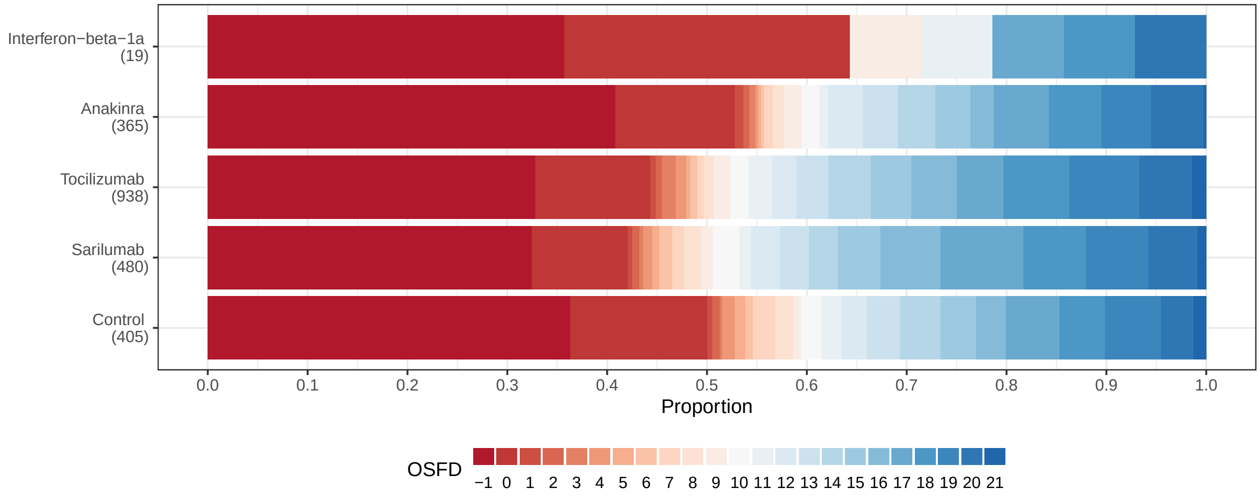

Figure 11: Empirical distribution of organ support free days (OSFD) for interferon-beta-1a, anakinra, tocilizumab, sarilumab, and control. This plot is restricted to the Immune Modulation Per Protocol population.

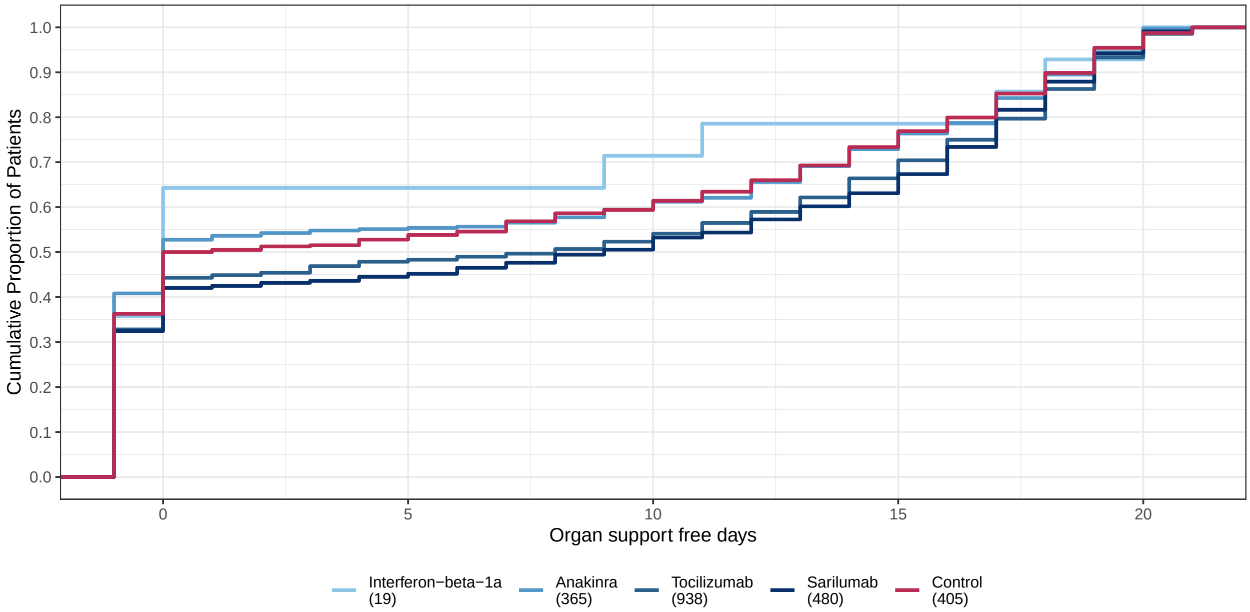

Figure 12: Empirical cumulative distribution of organ support free days (OSFD) for interferon-beta-1a, anakinra, tocilizumab, sarilumab, and control. This plot is restricted to the Immune Modulation Per Protocol population.

5.2 Sensitivity analysis of OSFD for Immune Modulation per protocol population

- Model: Primary analysis ordinal model
- Factors: Age, sex, site, time, Immune Modulation Therapy Domain interventions (control, interferon-beta-1a, anakinra, tocilizumab, and sarilumab)
- Population: Immune modulation per protocol
- Treatment effects for tocilizumab and sarilumab are modeled with independent priors.

Table 40: Odds ratio parameters for secondary analysis of OSFD in the per protocol population

	Mean	SD	Median	CrI
Interferon-beta-1a	1.20	0.55	1.09	(0.46, 2.55)
Anakinra	1.14	0.19	1.13	(0.81, 1.57)
Tocilizumab	1.58	0.20	1.56	(1.23, 1.99)
Sarilumab	1.67	0.26	1.65	(1.23, 2.24)
Age<39	4.57	0.77	4.52	(3.25, 6.24)
Age 40-49	2.51	0.33	2.49	(1.92, 3.22)
Age 50-59	2.19	0.23	2.18	(1.77, 2.68)
Age 70-79	0.54	0.07	0.54	(0.43, 0.68)
Age 80+	0.30	0.07	0.30	(0.18, 0.47)
Female	1.28	0.11	1.28	(1.08, 1.51)

Table 41: Posterior probabilities for secondary analysis of OSFD in the per protocol population

	Posterior Probability
Interferon-beta-1a is optimal	0.162
Interferon-beta-1a is superior to control	0.579
Interferon-beta-1a is futile (OR < 1.2)	0.587
Interferon-beta-1a is harmful (OR < 1)	0.421
Anakinra is optimal	0.000
Anakinra is superior to control	0.758
Anakinra is futile (OR < 1.2)	0.650
Anakinra is harmful (OR < 1)	0.242
Tocilizumab is optimal	0.254
Tocilizumab is superior to control	1.000
Tocilizumab is futile (OR < 1.2)	0.017
Tocilizumab is harmful (OR < 1)	0.000
Sarilumab is optimal	0.584
Sarilumab is superior to control	1.000
Sarilumab is futile (OR < 1.2)	0.018
Sarilumab is harmful (OR < 1)	0.001
Interferon-beta-1a equivalent to anakinra	0.309
Interferon-beta-1a equivalent to tocilizumab	0.227
Interferon-beta-1a equivalent to sarilumab	0.208
Anakinra equivalent to tocilizumab	0.124
Anakinra equivalent to sarilumab	0.075
Tocilizumab equivalent to sarilumab	0.841
Anakinra superior to tocilizumab	0.006
Anakinra superior to sarilumab	0.002
Tocilizumab superior to sarilumab	0.318

5.3 Sensitivity analysis of hospital survival for Immune Modulation per protocol population

- Model: Primary dichotomous model
- Factors: Age, sex, site, time, Immune Modulation Therapy Domain interventions (control, interferon-beta-1a, anakinra, tocilizumab, and sarilumab)
- Population: Immune modulation per protocol
- Treatment effects for tocilizumab and sarilumab are modeled with independent priors.

Table 42: Odds ratio parameters for secondary analysis of hospital survival in the per protocol population

	Mean	SD	Median	CrI
Interferon-beta-1a	1.50	0.89	1.28	(0.46, 3.78)
Anakinra	1.10	0.23	1.07	(0.71, 1.60)
Tocilizumab	1.60	0.26	1.58	(1.14, 2.16)
Sarilumab	1.80	0.36	1.76	(1.19, 2.61)
Age<39	9.21	2.95	8.71	(4.93, 16.24)
Age 40-49	3.50	0.67	3.43	(2.39, 5.03)
Age 50-59	2.84	0.40	2.81	(2.13, 3.71)
Age 70-79	0.52	0.07	0.52	(0.40, 0.68)
Age 80+	0.26	0.06	0.25	(0.15, 0.40)
Female	1.49	0.17	1.48	(1.19, 1.83)

Table 43: Posterior probabilities for secondary analysis of hospital survival in the per protocol population

	Posterior Probability
Interferon-beta-1a is optimal	0.279
Interferon-beta-1a is superior to control	0.685
Interferon-beta-1a is futile (OR < 1.2)	0.450
Interferon-beta-1a is harmful (OR < 1)	0.315
Anakinra is optimal	0.000
Anakinra is superior to control	0.639
Anakinra is futile (OR < 1.2)	0.701
Anakinra is harmful (OR < 1)	0.361
Tocilizumab is optimal	0.152
Tocilizumab is superior to control	0.998
Tocilizumab is futile (OR < 1.2)	0.046
Tocilizumab is harmful (OR < 1)	0.003
Sarilumab is optimal	0.568
Sarilumab is superior to control	0.998
Sarilumab is futile (OR < 1.2)	0.026
Sarilumab is harmful (OR < 1)	0.002
Interferon-beta-1a equivalent to anakinra	0.247
Interferon-beta-1a equivalent to tocilizumab	0.242
Interferon-beta-1a equivalent to sarilumab	0.220
Anakinra equivalent to tocilizumab	0.097
Anakinra equivalent to sarilumab	0.037
Tocilizumab equivalent to sarilumab	0.662
Anakinra superior to tocilizumab	0.007
Anakinra superior to sarilumab	0.003
Tocilizumab superior to sarilumab	0.229

6 Analyses of secondary endpoints

6.1 Secondary analysis of 90-day mortality

- Model: Primary TTE model
- Factors: Age, sex, time, Immune Modulation Therapy Domain interventions (control, interferon-beta-1a, anakinra, tocilizumab, and sarilumab)
- Treatment effects for tocilizumab and sarilumab are modeled with independent priors.
- Note: The pre-specified time-to-event model did not converge. As a result, the statisticians running the model made the following modifications:
  - The analysis population was changed from the Unblinded ITT population to the Immune Modulation ITT population, and intervention/interactions for domains other than Immune Modulation were removed from the model
  - Site factors were removed from the model (set to 0)

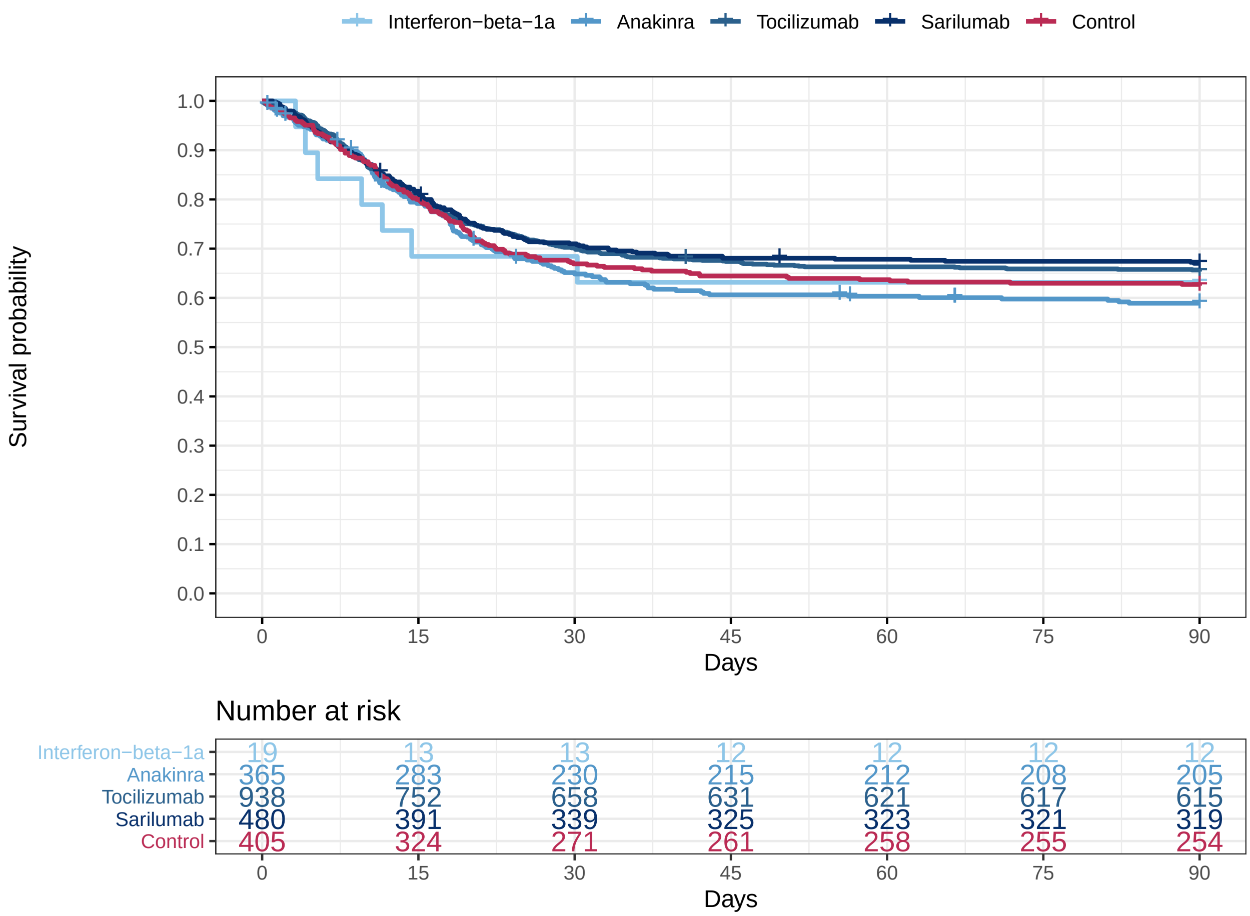

Figure 13: Empirical distribution of 90-day mortality for interferon-beta-1a, anakinra, tocilizumab, sarilumab, and control. This plot is restricted to the Immune Modulation ITT population.

Table 44: Summary of 2.5th, 10th, 25th, 50th, 75th, 90th, and 97.5th percentiles from the Kaplan-Meier estimates for mortality (in days). Displaying the observed percentiles for this outcome.

	2.5	10.0	25.0	50.0	75.0	90.0	97.5
Interferon-beta-1a	3.2	4.15	11.54	-	-	-	-
Anakinra	1.99	8.62	18.04	-	-	-	-
Tocilizumab	3.27	8.15	20.03	-	-	-	-
Sarilumab	3.08	8.26	20.38	-	-	-	-
Control	2.21	7.89	19.09	-	-	-	-

Table 45: Hazard ratio parameters for secondary analysis of 90-day survival. Hazard ratios above 1 indicate benefit, and hazard ratios below 1 indicate harm.

	Mean	SD	Median	CrI
Interferon-beta-1a	1.26	0.51	1.16	(0.60, 2.53)
Anakinra	1.15	0.16	1.13	(0.87, 1.49)
Tocilizumab	1.40	0.16	1.39	(1.11, 1.74)
Sarilumab	1.46	0.20	1.44	(1.11, 1.89)
Age<39	4.45	1.15	4.27	(2.71, 7.17)
Age 40-49	2.25	0.33	2.22	(1.69, 2.97)
Age 50-59	2.10	0.23	2.09	(1.70, 2.59)
Age 70-79	0.64	0.06	0.64	(0.54, 0.76)
Age 80+	0.36	0.05	0.36	(0.28, 0.48)
Female	1.31	0.11	1.30	(1.11, 1.53)

Table 46: Posterior probabilities for secondary analysis of 90-day survival

	Posterior Probability
Interferon-beta-1a is optimal	0.262
Interferon-beta-1a is superior to control	0.658
Interferon-beta-1a is futile (OR < 1.2)	0.539
Interferon-beta-1a is harmful (OR < 1)	0.342
Anakinra is optimal	0.005
Anakinra is superior to control	0.823
Anakinra is futile (OR < 1.2)	0.661
Anakinra is harmful (OR < 1)	0.177
Tocilizumab is optimal	0.254
Tocilizumab is superior to control	0.999
Tocilizumab is futile (OR < 1.2)	0.092
Tocilizumab is harmful (OR < 1)	0.002
Sarilumab is optimal	0.479
Sarilumab is superior to control	0.996
Sarilumab is futile (OR < 1.2)	0.085
Sarilumab is harmful (OR < 1)	0.004
Interferon-beta-1a equivalent to anakinra	0.374
Interferon-beta-1a equivalent to tocilizumab	0.327
Interferon-beta-1a equivalent to sarilumab	0.305
Anakinra equivalent to tocilizumab	0.411
Anakinra equivalent to sarilumab	0.306
Tocilizumab equivalent to sarilumab	0.917
Anakinra superior to tocilizumab	0.027
Anakinra superior to sarilumab	0.020
Tocilizumab superior to sarilumab	0.364

6.2 Secondary analysis of progression to intubation, ECMO, or death

- Model: Primary dichotomous model
- Factors: Age, sex, site, time, Immune Modulation Therapy Domain interventions (control, interferon-beta-1a, anakinra, tocilizumab, and sarilumab), COVID-19 Antiviral domain interventions (no antiviral, lopinavir–ritonavir, hydroxychloroquine, combination therapy), Corticosteroid domain interventions (no corticosteroid, fixed-duration corticosteroids, shock-dependent corticosteroids), Immunoglobulin domain interventions (no convalescent plasma, convalescent plasma, delayed convalescent plasma), and Anticoagulation interventions (usual care thromboprophylaxis, therapeutic anticoagulation)
- Treatment effects for tocilizumab and sarilumab are modeled with independent priors.

Table 47: Summary of progression to intubation, ECMO, or death for the Unblinded ITT population restricted to patients not on mechanical ventilation or ECMO at baseline

Intervention	# Patients (N)	# Progressors (y)	Progression Rate (y/N)
Interferon-beta-1a	12	6	0.500
Anakinra	228	122	0.535
Tocilizumab	613	266	0.434
Sarilumab	331	149	0.450
Control	276	147	0.533

Table 48: Summary of progression to intubation, ECMO, or death by component for the Unblinded ITT population restricted to patients not on mechanical ventilation or ECMO at baseline. Note that a patient may progress on more than one component of the composite endpoint, so the sum of events in this table may not match the total number of progressors.

Intervention	# Patients (N)	Death, n (%)	Intubation, n (%)	ECMO, n (%)
Interferon-beta-1a	12	3 (25)	6 (50)	0 (0)
Anakinra	228	75 (32.9)	103 (45.2)	2 (0.9)
Tocilizumab	613	173 (28.2)	214 (34.9)	6 (1)
Sarilumab	331	102 (30.8)	110 (33.2)	3 (0.9)
Control	276	88 (31.9)	121 (43.8)	3 (1.1)

Table 49: Odds ratio parameters for secondary analysis of freedom from progression to intubation, ECMO, or death

	Mean	SD	Median	CrI
Interferon-beta-1a	1.33	0.79	1.15	(0.39, 3.35)
Anakinra	1.19	0.26	1.16	(0.76, 1.78)
Tocilizumab	1.70	0.29	1.67	(1.20, 2.36)
Sarilumab	1.46	0.29	1.43	(0.98, 2.10)
Lopinavir−ritonavir	0.75	0.16	0.73	(0.49, 1.10)
Hydroxychloroquine	0.48	0.19	0.47	(0.16, 0.88)
Fixed-dose corticosteroids	2.98	1.17	2.78	(1.32, 5.87)
Shock-dependent corticosteroids	1.13	0.46	1.04	(0.50, 2.26)
Therapeutic anticogulation	0.89	0.15	0.88	(0.64, 1.21)
Convalescent plasma	0.84	0.10	0.83	(0.66, 1.05)
Delayed convalescent plasma	0.38	0.35	0.29	(0.06, 1.23)
Lopinavir−ritonavir*Tocilizumab combination	1.28	0.36	1.23	(0.73, 2.12)
Hydroxychloroquine*Tocilizumab combination	0.81	0.36	0.77	(0.25, 1.62)
Tocilizumab*Fixed-dose corticosteroids combination	5.10	2.24	4.68	(2.07, 10.75)
Lopinavir−ritonavir*Tocilizumab interaction	1.01	0.05	1.01	(0.91, 1.11)
Tocilizumab*Fixed-dose corticosteroids interaction	1.00	0.05	1.00	(0.91, 1.10)
Hydroxychloroquine*Tocilizumab interaction	1.01	0.05	1.01	(0.91, 1.11)
Age<39	4.75	1.05	4.62	(3.09, 7.17)
Age 40-49	2.29	0.33	2.27	(1.70, 3.00)
Age 50-59	1.74	0.21	1.73	(1.37, 2.18)
Age 70-79	0.64	0.08	0.63	(0.49, 0.82)
Age 80+	0.44	0.09	0.43	(0.28, 0.64)
Female	1.13	0.11	1.13	(0.94, 1.36)

Table 50: Posterior probabilities for secondary analysis of freedom from progression to intubation, ECMO, or death

	Posterior Probability
Interferon-beta-1a is optimal	0.239
Interferon-beta-1a is superior to control	0.596
Interferon-beta-1a is futile (OR < 1.2)	0.533
Interferon-beta-1a is harmful (OR < 1)	0.405
Anakinra is optimal	0.011
Anakinra is superior to control	0.750
Anakinra is futile (OR < 1.2)	0.564
Anakinra is harmful (OR < 1)	0.251
Tocilizumab is optimal	0.639
Tocilizumab is superior to control	0.999
Tocilizumab is futile (OR < 1.2)	0.025
Tocilizumab is harmful (OR < 1)	0.001
Sarilumab is optimal	0.111
Sarilumab is superior to control	0.969
Sarilumab is futile (OR < 1.2)	0.180
Sarilumab is harmful (OR < 1)	0.031
Lopinavir−ritonavir*tocilizumab combination OR > 1	0.774
Hydroxychloroquine*tocilizumab combination OR > 1	0.269
Fixed-dose steroids*tocilizumab combination OR > 1	1.000
Interferon-beta-1a equivalent to anakinra	0.244
Interferon-beta-1a equivalent to tocilizumab	0.212
Interferon-beta-1a equivalent to sarilumab	0.231
Anakinra equivalent to tocilizumab	0.158
Anakinra equivalent to sarilumab	0.421
Tocilizumab equivalent to sarilumab	0.548
Anakinra superior to tocilizumab	0.025
Anakinra superior to sarilumab	0.140
Tocilizumab superior to sarilumab	0.831

6.3 Secondary analysis of cardiovascular support-free days

- Model: Primary analysis ordinal model
- Factors: Age, sex, site, time, Immune Modulation Therapy Domain interventions (control, interferon-beta-1a, anakinra, tocilizumab, and sarilumab), COVID-19 Antiviral domain interventions (no antiviral, lopinavir−ritonavir, hydroxychloroquine, combination therapy), Corticosteroid domain interventions (no corticosteroid, fixed-duration corticosteroids, shock-dependent corticosteroids), Immunoglobulin domain interventions (no convalescent plasma, convalescent plasma, delayed convalescent plasma),

and Anticoagulation interventions (usual care thromboprophylaxis, therapeutic anticoagulation)

- Treatment effects for tocilizumab and sarilumab are modeled with independent priors.
- There are 26 missing values of days free of vasopressors/inotropes in the Unblinded ITT population due to missing daily data. These patients are removed from this analysis.

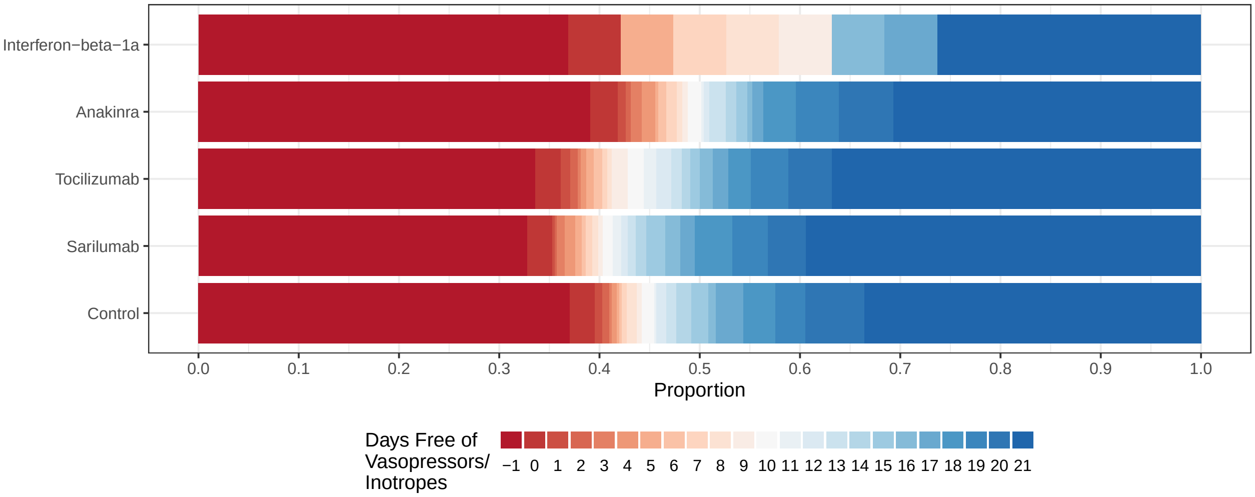

Figure 14: Empirical distribution of cardiovascular support-free days for interferon-beta-1a, anakinra, tocilizumab, sarilumab, and control. This plot is restricted to the Immune Modulation ITT population.

Table 51: Summary of cardiovascular support-free days in the Immune Modulation ITT population

Intervention	# Patients	# Known	Cardiovascular support-free days median (IQR)	Days free of cardiovascular support in survivors* median (IQR)
Interferon-beta-1a	19	19	7 (-1, 19)	16.5 (7.75, 21)
Anakinra	373	371	10 (-1, 21)	21 (15, 21)
Tocilizumab	947	944	15.5 (-1, 21)	21 (16, 21)
Sarilumab	482	479	18 (-1, 21)	21 (18, 21)
Control	405	405	15 (-1, 21)	21 (17, 21)

* Days free of cardiovascular support in survivors within 21 days

Table 52: Odds ratio parameters for secondary analysis of cardiovascular support-free days

	Mean	SD	Median	CrI
Interferon-beta-1a	1.08	0.47	0.99	(0.44, 2.20)
Anakinra	1.09	0.18	1.07	(0.78, 1.47)
Tocilizumab	1.46	0.19	1.44	(1.12, 1.87)
Sarilumab	1.60	0.24	1.58	(1.19, 2.12)
Lopinavir−ritonavir	0.73	0.11	0.72	(0.54, 0.96)
Hydroxychloroquine	0.64	0.13	0.63	(0.41, 0.90)
Fixed-dose corticosteroids	1.46	0.32	1.43	(0.93, 2.18)
Shock-dependent corticosteroids	1.19	0.27	1.16	(0.75, 1.78)
Therapeutic anticogulation	0.91	0.11	0.90	(0.71, 1.15)
Convalescent plasma	0.95	0.08	0.95	(0.80, 1.13)
Delayed convalescent plasma	0.89	0.54	0.76	(0.25, 2.33)
Lopinavir−ritonavir*Tocilizumab combination	1.07	0.21	1.05	(0.71, 1.56)
Hydroxychloroquine*Tocilizumab combination	0.93	0.23	0.91	(0.55, 1.44)
Tocilizumab*Fixed-dose corticosteroids combination	2.13	0.55	2.06	(1.24, 3.40)
Lopinavir−ritonavir*Tocilizumab interaction	1.01	0.05	1.01	(0.92, 1.11)
Tocilizumab*Fixed-dose corticosteroids interaction	1.01	0.05	1.01	(0.91, 1.11)
Hydroxychloroquine*Tocilizumab interaction	1.00	0.05	1.00	(0.91, 1.10)
Age<39	4.40	0.59	4.37	(3.38, 5.66)
Age 40-49	2.72	0.28	2.70	(2.22, 3.31)
Age 50-59	2.01	0.17	2.00	(1.70, 2.35)
Age 70-79	0.51	0.05	0.51	(0.43, 0.62)
Age 80+	0.28	0.05	0.28	(0.19, 0.39)
Female	1.12	0.07	1.12	(0.98, 1.27)

Table 53: Posterior probabilities for secondary analysis of cardiovascular support-free days

	Posterior Probability
Interferon-beta-1a is optimal	0.119
Interferon-beta-1a is superior to control	0.493
Interferon-beta-1a is futile (OR < 1.2)	0.674
Interferon-beta-1a is harmful (OR < 1)	0.507
Anakinra is optimal	0.001
Anakinra is superior to control	0.666
Anakinra is futile (OR < 1.2)	0.755
Anakinra is harmful (OR < 1)	0.334
Tocilizumab is optimal	0.183
Tocilizumab is superior to control	0.998
Tocilizumab is futile (OR < 1.2)	0.083
Tocilizumab is harmful (OR < 1)	0.003
Sarilumab is optimal	0.697
Sarilumab is superior to control	0.999
Sarilumab is futile (OR < 1.2)	0.028
Sarilumab is harmful (OR < 1)	0.001
Lopinavir−ritonavir*tocilizumab combination OR > 1	0.589
Hydroxychloroquine*tocilizumab combination OR > 1	0.346
Fixed-dose steroids*tocilizumab combination OR > 1	0.998
Interferon-beta-1a equivalent to anakinra	0.324
Interferon-beta-1a equivalent to tocilizumab	0.235
Interferon-beta-1a equivalent to sarilumab	0.191
Anakinra equivalent to tocilizumab	0.203
Anakinra equivalent to sarilumab	0.075
Tocilizumab equivalent to sarilumab	0.745
Anakinra superior to tocilizumab	0.014
Anakinra superior to sarilumab	0.003
Tocilizumab superior to sarilumab	0.224

6.4 Secondary analysis of respiratory support-free days

- Model: Primary analysis ordinal model
- Factors: Age, sex, site, time, Immune Modulation Therapy Domain interventions (control, interferon-beta-1a, anakinra, tocilizumab, and sarilumab), COVID-19 Antiviral domain interventions (no antiviral, lopinavir−ritonavir, hydroxychloroquine, combination therapy), Corticosteroid domain interventions (no corticosteroid, fixed-duration corticosteroids, shock-dependent corticosteroids), Immunoglobulin domain interventions (no convalescent plasma, convalescent plasma, delayed convalescent plasma), and Anticoagulation interventions (usual care thromboprophylaxis, therapeutic anticoagulation)

- Treatment effects for tocilizumab and sarilumab are modeled with independent priors.
- There are 26 missing values of days free of ventilation in the Unblinded ITT population due to missing daily data. These patients are removed from this analysis.

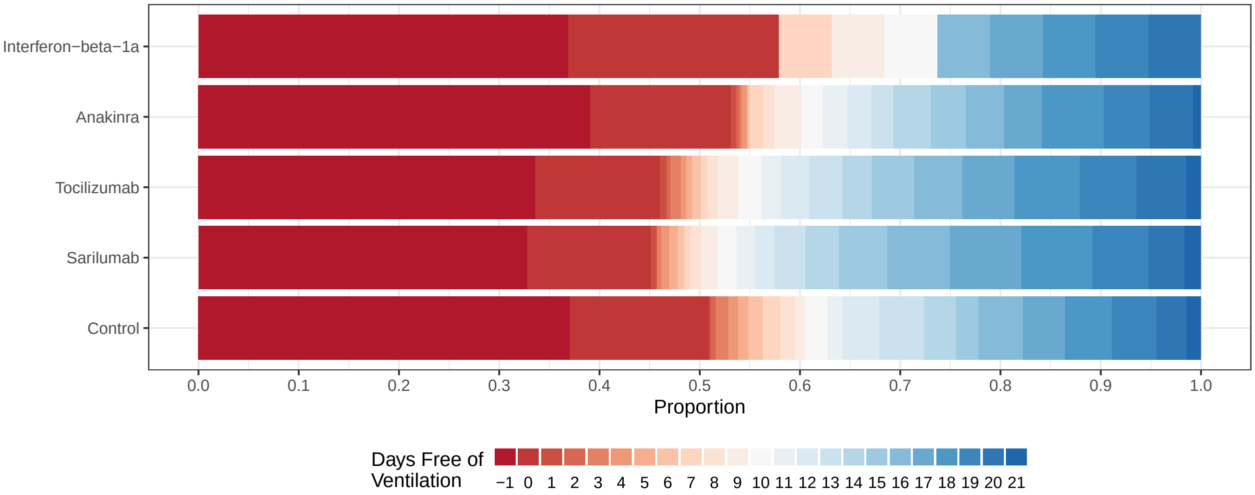

Figure 15: Empirical distribution of respiratory support-free days for interferon-beta-1a, anakinra, tocilizumab, sarilumab, and control. This plot is restricted to the Immune Modulation ITT population.

Table 54: Summary of respiratory support-free days for the Immune Modulation ITT population

Intervention	# Patients	# Known	Respiratory support-free days median (IQR)	Respiratory support-free days in survivors* median (IQR)
Interferon-beta-1a	19	19	0 (-1, 13)	9.5 (0, 17.25)
Anakinra	373	371	0 (-1, 15)	13.5 (3.75, 18)
Tocilizumab	947	944	6 (-1, 16)	14 (6, 18)
Sarilumab	482	479	8 (-1, 16.5)	15 (8, 18)
Control	405	405	0 (-1, 14)	13 (3.5, 17)

* Days free of respiratory support in survivors within 21 days

Table 55: Odds ratio parameters for secondary analysis of respiratory support-free days

	Mean	SD	Median	CrI
Interferon-beta-1a	1.36	0.55	1.27	(0.58, 2.72)
Anakinra	1.07	0.17	1.06	(0.78, 1.43)
Tocilizumab	1.52	0.19	1.51	(1.18, 1.92)
Sarilumab	1.61	0.23	1.60	(1.21, 2.09)
Lopinavir−ritonavir	0.81	0.12	0.80	(0.61, 1.07)
Hydroxychloroquine	0.65	0.14	0.65	(0.39, 0.93)
Fixed-dose corticosteroids	1.47	0.32	1.44	(0.94, 2.18)
Shock-dependent corticosteroids	1.25	0.28	1.22	(0.79, 1.87)
Therapeutic anticogulation	0.82	0.10	0.82	(0.65, 1.02)
Convalescent plasma	0.94	0.08	0.94	(0.80, 1.11)
Delayed convalescent plasma	0.61	0.35	0.53	(0.18, 1.49)
Lopinavir−ritonavir*Tocilizumab combination	1.24	0.24	1.22	(0.84, 1.79)
Hydroxychloroquine*Tocilizumab combination	0.99	0.25	0.97	(0.56, 1.55)
Tocilizumab*Fixed-dose corticosteroids combination	2.23	0.56	2.16	(1.32, 3.50)
Lopinavir−ritonavir*Tocilizumab interaction	1.01	0.05	1.01	(0.92, 1.11)
Tocilizumab*Fixed-dose corticosteroids interaction	1.01	0.05	1.00	(0.91, 1.11)
Hydroxychloroquine*Tocilizumab interaction	1.00	0.05	1.00	(0.91, 1.10)
Age<39	4.22	0.51	4.19	(3.32, 5.32)
Age 40-49	2.48	0.24	2.47	(2.03, 2.99)
Age 50-59	1.84	0.15	1.83	(1.56, 2.15)
Age 70-79	0.52	0.05	0.52	(0.43, 0.62)
Age 80+	0.30	0.05	0.30	(0.21, 0.42)
Female	1.14	0.07	1.14	(1.00, 1.28)

Table 56: Posterior probabilities for secondary analysis of respiratory support-free days

	Posterior Probability
Interferon-beta-1a is optimal	0.259
Interferon-beta-1a is superior to control	0.723
Interferon-beta-1a is futile (OR < 1.2)	0.445
Interferon-beta-1a is harmful (OR < 1)	0.277
Anakinra is optimal	0.000
Anakinra is superior to control	0.633
Anakinra is futile (OR < 1.2)	0.802
Anakinra is harmful (OR < 1)	0.367
Tocilizumab is optimal	0.220
Tocilizumab is superior to control	1.000
Tocilizumab is futile (OR < 1.2)	0.032
Tocilizumab is harmful (OR < 1)	0.000
Sarilumab is optimal	0.522
Sarilumab is superior to control	1.000
Sarilumab is futile (OR < 1.2)	0.021
Sarilumab is harmful (OR < 1)	0.000
Lopinavir−ritonavir*tocilizumab combination OR > 1	0.845
Hydroxychloroquine*tocilizumab combination OR > 1	0.455
Fixed-dose steroids*tocilizumab combination OR > 1	1.000
Interferon-beta-1a equivalent to anakinra	0.319
Interferon-beta-1a equivalent to tocilizumab	0.325
Interferon-beta-1a equivalent to sarilumab	0.297
Anakinra equivalent to tocilizumab	0.092
Anakinra equivalent to sarilumab	0.046
Tocilizumab equivalent to sarilumab	0.823
Anakinra superior to tocilizumab	0.004
Anakinra superior to sarilumab	0.002
Tocilizumab superior to sarilumab	0.310

6.5 Secondary analysis of length of ICU stay

- Model: Primary TTE model
- Factors: Age, sex, time, Immune Modulation Therapy Domain interventions (control, interferon-beta-1a, anakinra, tocilizumab, and sarilumab)
- Treatment effects for tocilizumab and sarilumab are modeled with independent priors.
- Note: The pre-specified time-to-event model did not converge. As a result, the statisticians running the model made the following modifications:
  - The analysis population was changed from the Unblinded ITT population to the Immune Modula-

tion ITT population, and intervention/interactions for domains other than Immune Modulation were removed from the model

– Site factors were removed from the model (set to 0)

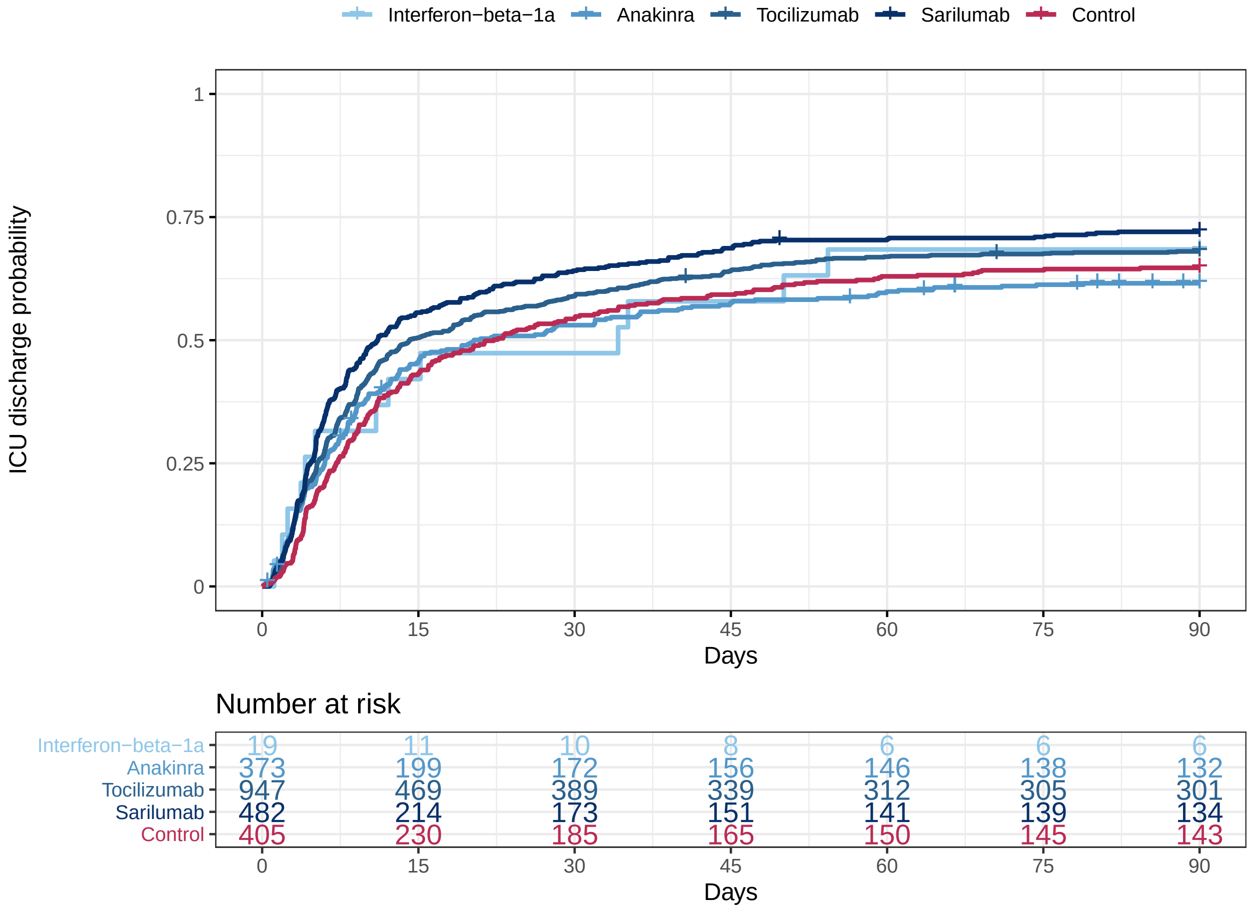

Figure 16: Empirical distribution of length of ICU stay for interferon-beta-1a, anakinra, tocilizumab, sarilumab, and control. This plot is restricted to the Immune Modulation ITT population.

Table 57: Summary of 2.5th, 10th, 25th, 50th, 75th, 90th, and 97.5th percentiles from the Kaplan-Meier estimates for length of ICU stay (in days). Displaying the observed percentiles for this outcome.

	2.5	10.0	25.0	50.0	75.0	90.0	97.5
Interferon-beta-1a	1.15	1.92	4.12	34.17	-	-	-
Anakinra	1.03	2.68	6.01	20.33	-	-	-
Tocilizumab	1.17	2.75	5.29	14.17	-	-	-
Sarilumab	1.21	2.78	4.6	11.09	-	-	-
Control	1.82	3.73	7.13	22.23	-	-	-

Table 58: Hazard ratio parameters for secondary analysis of length of ICU stay

	Mean	SD	Median	CrI
Interferon-beta-1a	1.21	0.33	1.17	(0.65, 1.95)
Anakinra	1.10	0.12	1.10	(0.89, 1.36)
Tocilizumab	1.30	0.11	1.29	(1.10, 1.52)
Sarilumab	1.42	0.14	1.41	(1.16, 1.72)
Age<39	2.61	0.26	2.60	(2.12, 3.16)
Age 40-49	1.70	0.14	1.69	(1.44, 1.99)
Age 50-59	1.62	0.11	1.62	(1.42, 1.85)
Age 70-79	0.73	0.06	0.73	(0.62, 0.86)
Age 80+	0.63	0.10	0.62	(0.45, 0.84)
Female	1.18	0.06	1.18	(1.06, 1.32)

Table 59: Posterior probabilities for secondary analysis of length of ICU stay

	Posterior Probability
Interferon-beta-1a is optimal	0.246
Interferon-beta-1a is superior to control	0.710
Interferon-beta-1a is futile (OR < 1.2)	0.534
Interferon-beta-1a is harmful (OR < 1)	0.290
Anakinra is optimal	0.001
Anakinra is superior to control	0.802
Anakinra is futile (OR < 1.2)	0.802
Anakinra is harmful (OR < 1)	0.198
Tocilizumab is optimal	0.066
Tocilizumab is superior to control	0.999
Tocilizumab is futile (OR < 1.2)	0.184
Tocilizumab is harmful (OR < 1)	0.001
Sarilumab is optimal	0.687
Sarilumab is superior to control	1.000
Sarilumab is futile (OR < 1.2)	0.050
Sarilumab is harmful (OR < 1)	0.000
Interferon-beta-1a equivalent to anakinra	0.455
Interferon-beta-1a equivalent to tocilizumab	0.467
Interferon-beta-1a equivalent to sarilumab	0.408
Anakinra equivalent to tocilizumab	0.591
Anakinra equivalent to sarilumab	0.209
Tocilizumab equivalent to sarilumab	0.906
Anakinra superior to tocilizumab	0.026
Anakinra superior to sarilumab	0.002
Tocilizumab superior to sarilumab	0.099

6.6 Secondary analysis of length of hospital stay

- Model: Primary TTE model
- Factors: Age, sex, time, Immune Modulation Therapy Domain interventions (control, interferon-beta-1a, anakinra, tocilizumab, and sarilumab)
- Treatment effects for tocilizumab and sarilumab are modeled with independent priors.
- Note: The pre-specified time-to-event model did not converge. As a result, the statisticians running the model made the following modifications:
  - The analysis population was changed from the Unblinded ITT population to the Immune Modulation ITT population, and intervention/interactions for domains other than Immune Modulation were removed from the model

– Site factors were removed from the model (set to 0)

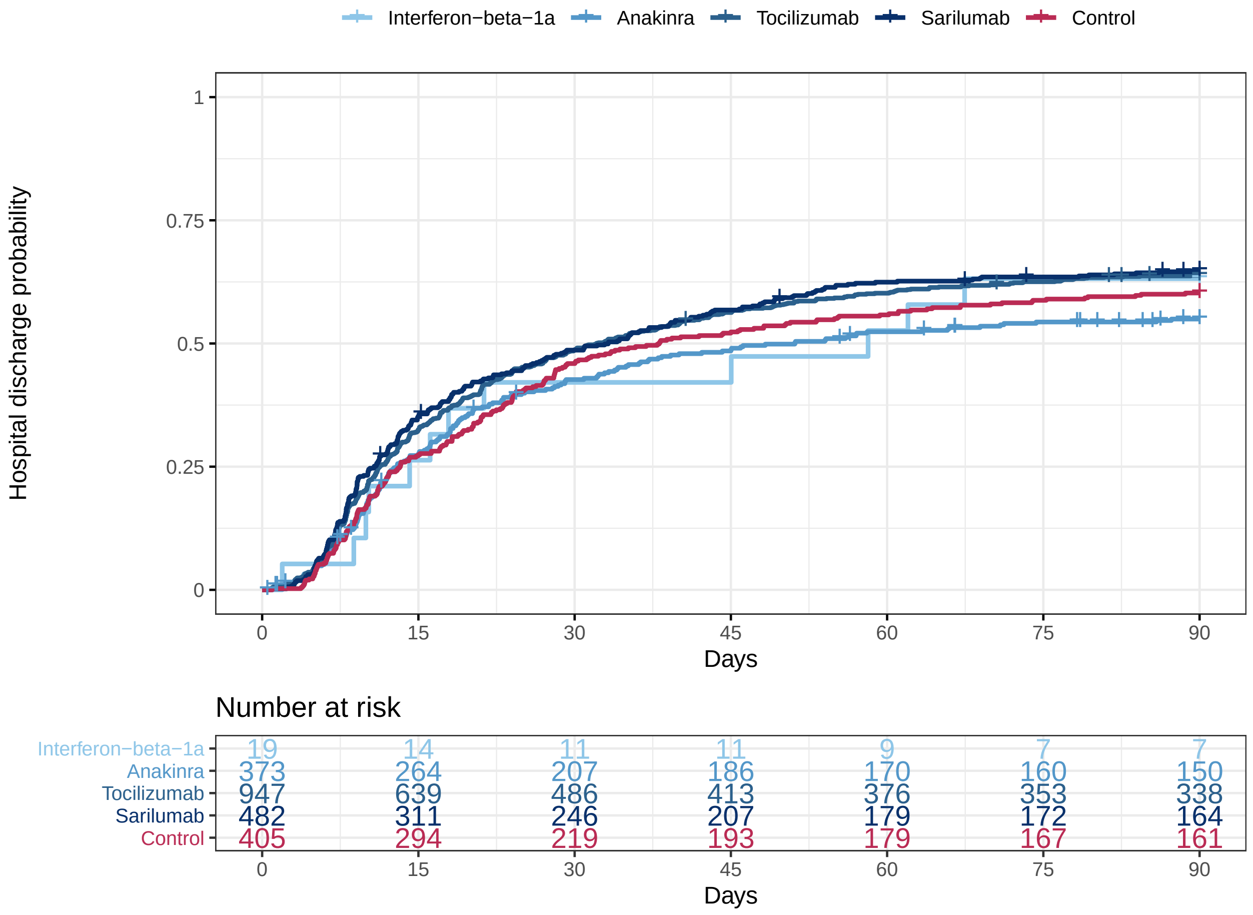

Figure 17: Empirical distribution of length of hospital stay for interferon-beta-1a, anakinra, tocilizumab, sarilumab, and control. This plot is restricted to the Immune Modulation ITT population.

Table 60: Summary of 2.5th, 10th, 25th, 50th, 75th, 90th, and 97.5th percentiles from the Kaplan-Meier estimates for length of hospital stay (in days). Displaying the observed percentiles for this outcome.

	2.5	10.0	25.0	50.0	75.0	90.0	97.5
Interferon-beta-1a	1.92	8.8	14.16	58.18	-	-	-
Anakinra	3.94	7.07	12.92	51.14	-	-	-
Tocilizumab	3.75	7.03	11.29	32.11	-	-	-
Sarilumab	4.18	6.45	10.75	33.22	-	-	-
Control	4.89	7.36	13.26	38.11	-	-	-

Table 61: Hazard ratio parameters for secondary analysis of length of hospital stay

	Mean	SD	Median	CrI
Interferon-beta-1a	1.18	0.34	1.15	(0.62, 1.94)
Anakinra	1.05	0.12	1.04	(0.84, 1.30)
Tocilizumab	1.31	0.11	1.31	(1.11, 1.55)
Sarilumab	1.31	0.14	1.30	(1.06, 1.59)
Age<39	3.16	0.33	3.15	(2.57, 3.84)
Age 40-49	1.90	0.16	1.89	(1.60, 2.23)
Age 50-59	1.81	0.13	1.81	(1.57, 2.07)
Age 70-79	0.70	0.06	0.70	(0.59, 0.83)
Age 80+	0.44	0.08	0.43	(0.29, 0.62)
Female	1.22	0.07	1.22	(1.09, 1.37)

Table 62: Posterior probabilities for secondary analysis of length of hospital stay

	Posterior Probability
Interferon-beta-1a is optimal	0.292
Interferon-beta-1a is superior to control	0.686
Interferon-beta-1a is futile (OR < 1.2)	0.558
Interferon-beta-1a is harmful (OR < 1)	0.314
Anakinra is optimal	0.001
Anakinra is superior to control	0.647
Anakinra is futile (OR < 1.2)	0.897
Anakinra is harmful (OR < 1)	0.352
Tocilizumab is optimal	0.373
Tocilizumab is superior to control	1.000
Tocilizumab is futile (OR < 1.2)	0.156
Tocilizumab is harmful (OR < 1)	0.001
Sarilumab is optimal	0.334
Sarilumab is superior to control	0.994
Sarilumab is futile (OR < 1.2)	0.224
Sarilumab is harmful (OR < 1)	0.006
Interferon-beta-1a equivalent to anakinra	0.430
Interferon-beta-1a equivalent to tocilizumab	0.446
Interferon-beta-1a equivalent to sarilumab	0.447
Anakinra equivalent to tocilizumab	0.301
Anakinra equivalent to sarilumab	0.348
Tocilizumab equivalent to sarilumab	0.985
Anakinra superior to tocilizumab	0.005
Anakinra superior to sarilumab	0.008
Tocilizumab superior to sarilumab	0.539

6.7 Secondary analysis of WHO scale at 14 days

- Model: Primary analysis ordinal model
- Factors: Age, sex, site, time, Immune Modulation Therapy Domain interventions (control, interferon-beta-1a, anakinra, tocilizumab, and sarilumab), COVID-19 Antiviral domain interventions (no antiviral, lopinavir−ritonavir, hydroxychloroquine, combination therapy), Corticosteroid domain interventions (no corticosteroid, fixed-duration corticosteroids, shock-dependent corticosteroids), Immunoglobulin domain interventions (no convalescent plasma, convalescent plasma, delayed convalescent plasma), and Anticoagulation interventions (usual care thromboprophylaxis, therapeutic anticoagulation)
- Treatment effects for tocilizumab and sarilumab are modeled with independent priors.
- There are 2 missing values of WHO scale at day 14 due to missing daily data. These patients are removed from this analysis.

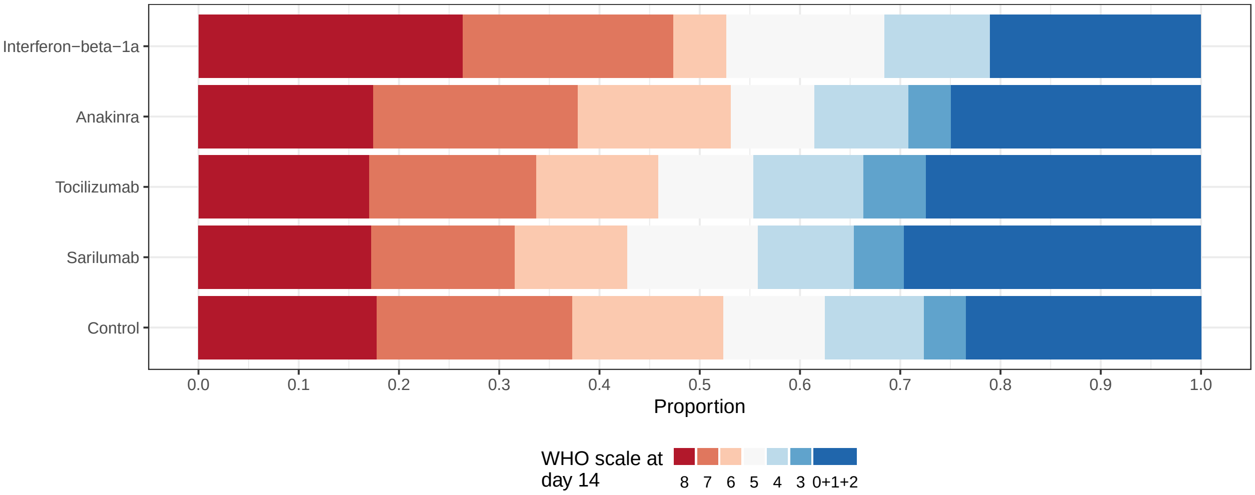

Figure 18: Empirical distribution of modified WHO scale at day 14 for interferon-beta-1a, anakinra, tocilizumab, sarilumab, and control. This plot is restricted to the Immune Modulation ITT population.

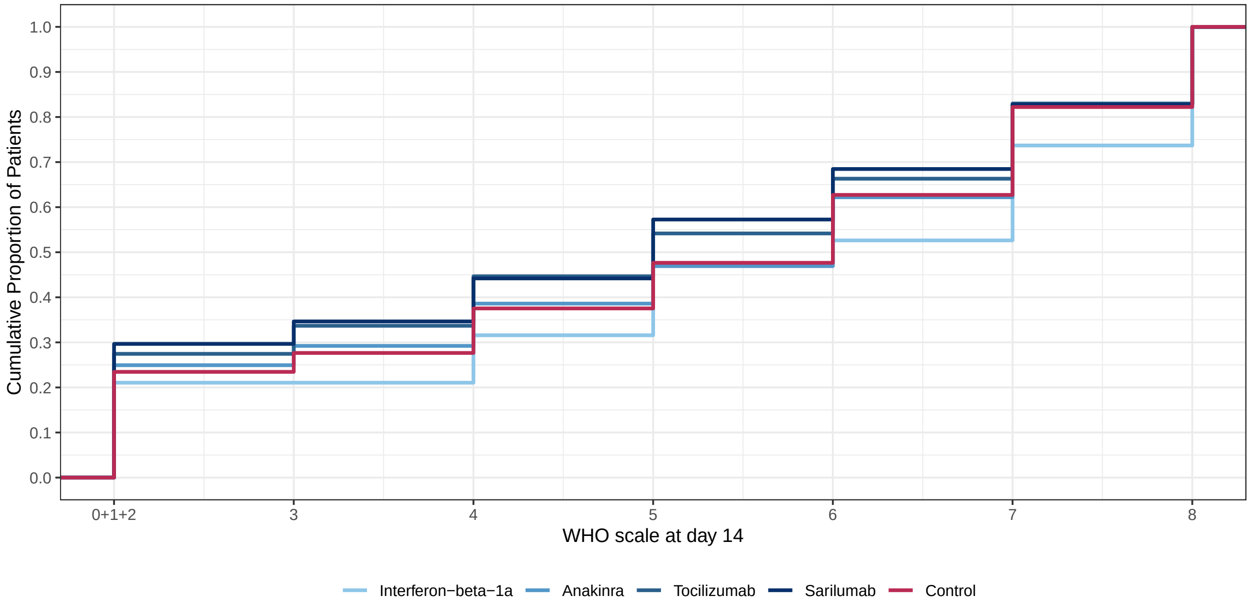

Figure 19: Empirical cumulative distribution of WHO scale for interferon-beta-1a, anakinra, tocilizumab, sarilumab, and control. This plot is restricted to the Immune Modulation ITT population.

Table 63: Odds ratio parameters for secondary analysis of WHO scale at 14 days

	Mean	SD	Median	CrI
Interferon-beta-1a	1.22	0.50	1.13	(0.52, 2.46)
Anakinra	1.19	0.18	1.18	(0.87, 1.60)
Tocilizumab	1.54	0.19	1.53	(1.20, 1.95)
Sarilumab	1.67	0.24	1.65	(1.25, 2.18)
Lopinavir−ritonavir	0.88	0.12	0.88	(0.67, 1.15)
Hydroxychloroquine	0.79	0.15	0.78	(0.52, 1.10)
Fixed-dose corticosteroids	1.34	0.29	1.31	(0.86, 2.00)
Shock-dependent corticosteroids	0.99	0.22	0.96	(0.63, 1.49)
Therapeutic anticogulation	0.79	0.09	0.78	(0.62, 0.98)
Convalescent plasma	0.92	0.08	0.92	(0.78, 1.08)
Delayed convalescent plasma	0.71	0.38	0.63	(0.23, 1.66)
Lopinavir−ritonavir*Tocilizumab combination	1.38	0.26	1.36	(0.95, 1.95)
Hydroxychloroquine*Tocilizumab combination	1.21	0.28	1.19	(0.74, 1.81)
Tocilizumab*Fixed-dose corticosteroids combination	2.06	0.51	2.00	(1.23, 3.26)
Lopinavir−ritonavir*Tocilizumab interaction	1.01	0.05	1.01	(0.92, 1.12)
Tocilizumab*Fixed-dose corticosteroids interaction	1.00	0.05	1.00	(0.91, 1.11)
Hydroxychloroquine*Tocilizumab interaction	1.00	0.05	1.00	(0.90, 1.10)
Age<39	4.35	0.58	4.31	(3.35, 5.58)
Age 40-49	2.31	0.22	2.29	(1.92, 2.78)
Age 50-59	1.74	0.14	1.74	(1.49, 2.03)
Age 70-79	0.55	0.05	0.54	(0.46, 0.64)
Age 80+	0.27	0.04	0.27	(0.19, 0.37)
Female	1.05	0.07	1.05	(0.92, 1.19)

Table 64: Posterior probabilities for secondary analysis of WHO scale at 14 days

	Posterior Probability
Interferon-beta-1a is optimal	0.160
Interferon-beta-1a is superior to control	0.620
Interferon-beta-1a is futile (OR < 1.2)	0.556
Interferon-beta-1a is harmful (OR < 1)	0.380
Anakinra is optimal	0.002
Anakinra is superior to control	0.856
Anakinra is futile (OR < 1.2)	0.545
Anakinra is harmful (OR < 1)	0.144
Tocilizumab is optimal	0.206
Tocilizumab is superior to control	1.000
Tocilizumab is futile (OR < 1.2)	0.025
Tocilizumab is harmful (OR < 1)	0.000
Sarilumab is optimal	0.632
Sarilumab is superior to control	1.000
Sarilumab is futile (OR < 1.2)	0.013
Sarilumab is harmful (OR < 1)	0.000
Lopinavir−ritonavir*tocilizumab combination OR > 1	0.949
Hydroxychloroquine*tocilizumab combination OR > 1	0.776
Fixed-dose steroids*tocilizumab combination OR > 1	0.998
Interferon-beta-1a equivalent to anakinra	0.347
Interferon-beta-1a equivalent to tocilizumab	0.276
Interferon-beta-1a equivalent to sarilumab	0.233
Anakinra equivalent to tocilizumab	0.273
Anakinra equivalent to sarilumab	0.125
Tocilizumab equivalent to sarilumab	0.802
Anakinra superior to tocilizumab	0.021
Anakinra superior to sarilumab	0.006
Tocilizumab superior to sarilumab	0.260

6.8 Secondary analysis of major thrombotic events or death

Table 65: Summary of major thrombotic events or death by intervention in the Immune Modulation ITT population. Note that this table displays the number of patients with one or more major thrombotic events rather than the total number of major thrombotic events.

Intervention	Patients (N)	Known (n)	Death	Major thrombotic event	Death or major thrombotic event (y)	Death or major thrombotic event rate (y/n)
Overall	2226	1137	409	97	456	0.401
Interferon-beta-1a	19	14	5	0	5	0.357
Anakinra	373	97	45	6	48	0.495
Tocilizumab	947	505	174	40	192	0.380
Sarilumab	482	276	91	26	104	0.377
Control	405	245	94	25	107	0.437
Pooled IL-6ra	1429	781	265	66	296	0.379

Table 66: Summary of the number of major thrombotic events by intervention in the Immune Modulation ITT population

Intervention	Patients	Known	Patients with major thrombotic events	Total number of major thrombotic events
Overall	2226	1138	97	106
Interferon-beta-1a	19	14	0	0
Anakinra	373	97	6	8
Tocilizumab	947	506	40	42
Sarilumab	482	276	26	31
Control	405	245	25	25

Table 67: Summary of the components of major thrombotic events by intervention in the Immune Modulation ITT population

Intervention	# Major thrombotic events	Pulmonary embolism	Myocardial infarction	Ischemic cerebrovascular event	Systemic arterial thromboembolism
Overall	106	61	16	21	8
Interferon-beta-1a	0	0	0	0	0
Anakinra	8	4	2	2	0
Tocilizumab	42	20	8	12	2
Sarilumab	31	19	6	3	3
Control	25	18	0	4	3

- Model: Primary dichotomous model
- Factors: Age, sex, site, time, Immune Modulation Therapy Domain interventions (control, interferon-beta-1a, anakinra, tocilizumab, and sarilumab)
- Treatment effects for tocilizumab and sarilumab are pooled into a single IL-6ra effect.

Table 68: Odds ratio parameters for the secondary analysis of freedom from major thrombotic events and death

	Mean	SD	Median	CrI
Interferon-beta-1a	1.63	1.00	1.38	(0.51, 4.17)
Anakinra	1.01	0.29	0.97	(0.57, 1.69)
Pooled IL-6ra	1.78	0.34	1.75	(1.22, 2.55)
Age<39	8.51	3.63	7.75	(3.76, 17.65)
Age 40-49	3.18	0.76	3.08	(1.97, 4.91)
Age 50-59	2.88	0.52	2.82	(2.01, 3.99)
Age 70-79	0.54	0.10	0.53	(0.37, 0.75)
Age 80+	0.28	0.09	0.26	(0.14, 0.48)
Female	1.36	0.21	1.35	(1.00, 1.80)

Table 69: Posterior probabilities for the secondary analysis of freedom from major thrombotic events and death

	Posterior Probability
Interferon-beta-1a is optimal	0.335
Interferon-beta-1a is superior to control	0.730
Interferon-beta-1a is futile (OR < 1.2)	0.392
Interferon-beta-1a is harmful (OR < 1)	0.270
Anakinra is optimal	0.005
Anakinra is superior to control	0.460
Anakinra is futile (OR < 1.2)	0.772
Anakinra is harmful (OR < 1)	0.540
Pooled IL-6ra is optimal	0.659
Pooled IL-6ra is superior to control	0.999
Pooled IL-6ra is futile (OR < 1.2)	0.021
Pooled IL-6ra is harmful (OR < 1)	0.001
Interferon-beta-1a equivalent to anakinra	0.203
Interferon-beta-1a equivalent to pooled IL-6ra	0.236
Anakinra equivalent to pooled IL-6ra	0.046
Anakinra superior to pooled IL-6ra	0.007

6.9 Secondary analysis of major thrombotic events

Table 70: Summary of major thrombotic events by intervention in the Immune Modulation ITT population. Note that this table displays the number of patients with one or more major thrombotic events rather than the total number of major thrombotic events.

Intervention	Patients (N)	Known (n)	Major thrombotic event	Major thrombotic event rate (y/n)
Overall	2226	1138	97	0.085
Interferon-beta-1a	19	14	0	0.000
Anakinra	373	97	6	0.062
Tocilizumab	947	506	40	0.079
Sarilumab	482	276	26	0.094
Control	405	245	25	0.102
Pooled IL-6ra	1429	782	66	0.084

- Model: Primary dichotomous model

- Factors: Age, sex, site, time, Immune Modulation Therapy Domain interventions (control, interferon-beta-1a, anakinra, tocilizumab, and sarilumab)
- Treatment effects for tocilizumab and sarilumab are pooled into a single IL-6ra effect.

Table 71: Odds ratio parameters for the secondary analysis of freedom from major thrombotic events

	Mean	SD	Median	CrI
Interferon-beta-1a	3.07	3.07	2.16	(0.52, 11.15)
Anakinra	1.79	0.91	1.58	(0.66, 4.10)
Pooled IL-6ra	1.24	0.37	1.19	(0.68, 2.09)
Age<39	2.83	1.80	2.40	(0.92, 7.18)
Age 40-49	1.40	0.51	1.31	(0.67, 2.66)
Age 50-59	1.83	0.55	1.75	(1.00, 3.14)
Age 70-79	1.01	0.29	0.97	(0.57, 1.70)
Age 80+	2.93	2.22	2.35	(0.79, 8.49)
Female	1.10	0.27	1.07	(0.67, 1.71)

Table 72: Posterior probabilities for the secondary analysis of freedom from major thrombotic events

	Posterior Probability
Interferon-beta-1a is optimal	0.612
Interferon-beta-1a is superior to control	0.843
Interferon-beta-1a is futile (OR < 1.2)	0.220
Interferon-beta-1a is harmful (OR < 1)	0.157
Anakinra is optimal	0.312
Anakinra is superior to control	0.844
Anakinra is futile (OR < 1.2)	0.272
Anakinra is harmful (OR < 1)	0.156
Pooled IL-6ra is optimal	0.057
Pooled IL-6ra is superior to control	0.729
Pooled IL-6ra is futile (OR < 1.2)	0.513
Pooled IL-6ra is harmful (OR < 1)	0.271
Interferon-beta-1a equivalent to anakinra	0.157
Interferon-beta-1a equivalent to pooled IL-6ra	0.145
Anakinra equivalent to pooled IL-6ra	0.277
Anakinra superior to pooled IL-6ra	0.745

6.10 Secondary analysis of major bleeding events

Table 73: Summary of major bleeds by intervention for the Immune Modulation ITT population

Intervention	Patients (N)	Known (n)	Patients with Major Bleeds (y)	Major Bleed Rate (y/n)
Overall	2226	458	8	0.017
Interferon-beta-1a	19	10	1	0.100
Anakinra	373	37	1	0.027
Tocilizumab	947	170	4	0.024
Sarilumab	482	93	0	0.000
Control	405	148	2	0.014
Pooled IL-6ra	1429	263	4	0.015

- Model: Primary dichotomous model
- Factors: Age, sex, Immune Modulation Therapy Domain interventions (control, interferon-beta-1a, anakinra, tocilizumab, and sarilumab)
- Treatment effects for tocilizumab and sarilumab are pooled into a single IL-6ra effect.
- Due to the small number of major bleeding events and concerns about model stability, site and time factors were removed from this model.

Table 74: Odds ratio parameters for the secondary analysis of freedom from major bleeding events

	Mean	SD	Median	CrI
Interferon-beta-1a	0.88	0.96	0.59	(0.12, 3.47)
Anakinra	1.31	1.26	0.96	(0.23, 4.44)
Pooled IL-6ra	1.46	0.93	1.24	(0.39, 3.89)
Age<39	8.35	10.10	5.25	(0.86, 34.70)
Age 40-49	1.88	1.70	1.39	(0.34, 6.41)
Age 50-59	5.26	5.37	3.71	(0.85, 19.63)
Age 70-79	0.79	0.57	0.64	(0.19, 2.30)
Age 80+	0.78	0.85	0.54	(0.13, 2.87)
Female	0.49	0.33	0.41	(0.13, 1.32)

Table 75: Posterior probabilities for the secondary analysis of freedom from major bleeding events

	Posterior Probability
Interferon-beta-1a is optimal	0.134
Interferon-beta-1a is superior to control	0.270
Interferon-beta-1a is harmful (OR < 1)	0.730
Anakinra is optimal	0.280
Anakinra is superior to control	0.474
Anakinra is harmful (OR < 1)	0.526
Pooled IL-6ra is optimal	0.414
Pooled IL-6ra is superior to control	0.637
Pooled IL-6ra is harmful (OR < 1)	0.363
Interferon-beta-1a equivalent to anakinra	0.123
Interferon-beta-1a equivalent to pooled IL-6ra	0.112
Anakinra equivalent to pooled IL-6ra	0.159
Anakinra superior to pooled IL-6ra	0.386

7 Subgroup analyses

7.1 Subgroup analyses by baseline C-reactive protein subgroup

In this section, subgroup analyses are performed by baseline C-reactive protein (CRP) subgroup. The CRP subgroups are defined based on terciles of CRP. Patients with CRP lower than 85 μg/ml are included in the lowest CRP subgroup. Patients with CRP greater/equal to 85 and less than 169 μg/ml are included in the middle CRP subgroup. Patients with CRP greater or equal to 169 μg/ml are included in the highest CRP subgroup. Patients with unknown CRP are included in a fourth “unknown” subgroup.

7.1.1 Data summaries of baseline CRP in the Immune Modulation ITT population

Table 76: Summary of baseline CRP subgroup by intervention

Intervention	Patients	Lower CRP tercile	Middle CRP tercile	Upper CRP tercile	Unknown CRP
Interferon-beta-1a	19	6 (31.6%)	5 (26.3%)	3 (15.8%)	5 (26.3%)
Anakinra	373	110 (29.5%)	118 (31.6%)	96 (25.7%)	49 (13.1%)
Tocilizumab	947	237 (25%)	261 (27.6%)	280 (29.6%)	169 (17.8%)
Sarilumab	482	135 (28%)	139 (28.8%)	142 (29.5%)	66 (13.7%)
Control	405	78 (19.3%)	85 (21%)	91 (22.5%)	151 (37.3%)
Pooled IL-6ra	1429	372 (26%)	400 (28%)	422 (29.5%)	235 (16.4%)
Overall	2226	566 (25.4%)	608 (27.3%)	612 (27.5%)	440 (19.8%)

Table 77: Summary of subjects by baseline CRP subgroup in the Immune Modulation ITT population

Intervention	Patients	Known	Deaths (%)	OSFD median (IQR)	OSFD in survivors* median (IQR)
**Lower tercile of CRP**					
Interferon-beta-1a	6	6	2 (33.3%)	4.5 (-0.75, 10.5)	10 (6.75, 12.75)
Anakinra	110	109	42 (38.5%)	1 (-1, 17)	15 (3, 19)
Tocilizumab	237	235	79 (33.6%)	10 (-1, 18)	17 (10, 19)
Sarilumab	135	135	45 (33.3%)	11 (-1, 17)	16 (11.25, 18)
Control	78	78	30 (38.5%)	10 (-1, 17)	16.5 (11.75, 18.25)
Pooled IL-6ra	372	370	124 (33.5%)	10 (-1, 18)	17 (10, 19)
**Middle tercile of CRP**					
Interferon-beta-1a	5	5	3 (60%)	-1 (-1, 11)	15.5 (13.25, 17.75)
Anakinra	118	116	45 (38.8%)	0 (-1, 14)	13 (5.5, 17)
Tocilizumab	261	260	83 (31.9%)	8.5 (-1, 16)	14 (8, 18)
Sarilumab	139	138	43 (31.2%)	9.5 (-1, 17)	15 (8.5, 18)
Control	85	85	29 (34.1%)	9 (-1, 15)	14 (9, 17)
Pooled IL-6ra	400	398	126 (31.7%)	9 (-1, 16)	14.5 (8, 18)
**Upper tercile of CRP**					
Interferon-beta-1a	3	3	0 (0%)	16 (8, 16.5)	16 (8, 16.5)
Anakinra	96	94	40 (42.6%)	0 (-1, 14)	13 (0.5, 16.75)
Tocilizumab	280	278	103 (37.1%)	1.5 (-1, 16)	14 (4.5, 18)
Sarilumab	142	141	48 (34%)	8 (-1, 16)	15 (9, 17)
Control	91	91	32 (35.2%)	0 (-1, 14)	12 (0, 16.5)
Pooled IL-6ra	422	419	151 (36%)	3 (-1, 16)	14 (7, 17)
**Unknown CRP**					
Interferon-beta-1a	5	5	2 (40%)	0 (-1, 0)	0 (0, 10)
Anakinra	49	46	18 (39.1%)	3 (-1, 14.75)	13.5 (7.75, 17.25)
Tocilizumab	169	165	52 (31.5%)	7 (-1, 15)	14 (6, 17)
Sarilumab	66	66	21 (31.8%)	7 (-1, 16.75)	14 (7, 18)
Control	151	151	59 (39.1%)	0 (-1, 13.5)	11 (0, 17)
Pooled IL-6ra	235	231	73 (31.6%)	7 (-1, 15.5)	14 (6, 18)

* Days free of organ support in survivors within 21 days

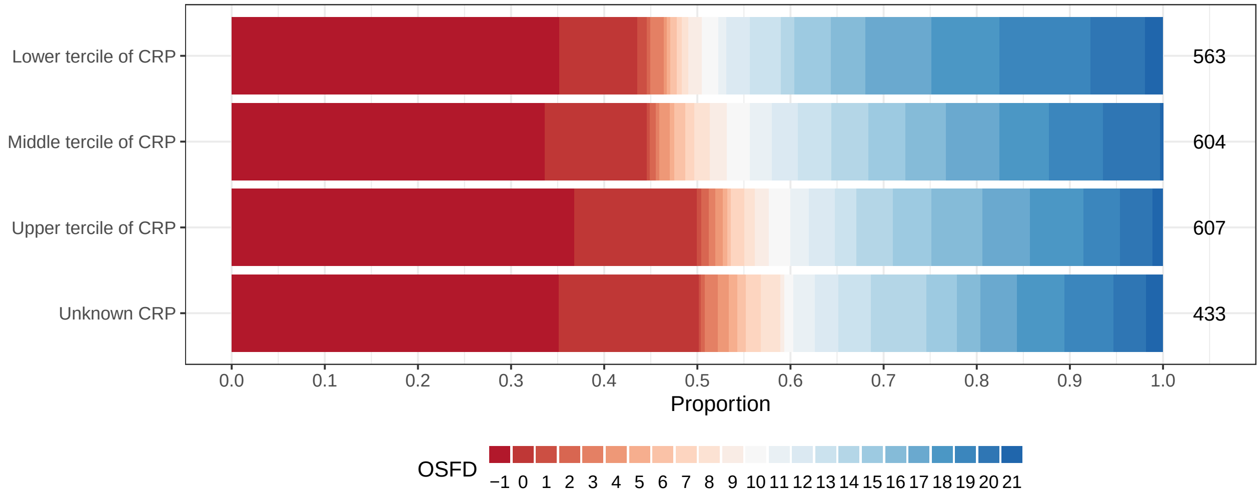

Figure 20: Empirical distribution of organ support free days (OSFD) by baseline CRP subgroup. This plot is restricted to the Immune Modulation ITT population.

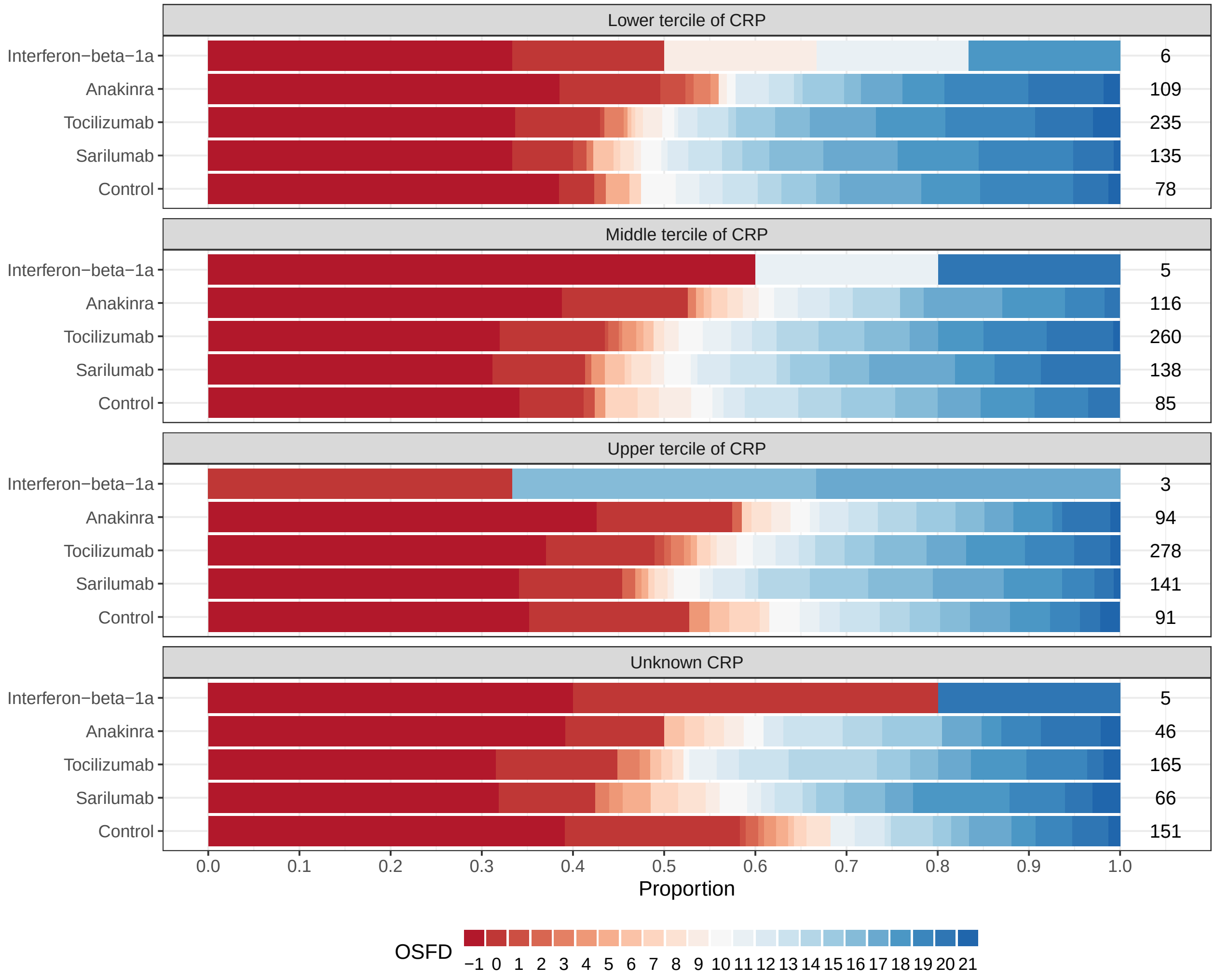

Figure 21: Empirical distribution of organ support free days (OSFD) for interferon-beta-1a, anakinra, tocilizumab, sarilumab, and control by baseline CRP subgroup. This plot is restricted to the Immune Modulation ITT population.

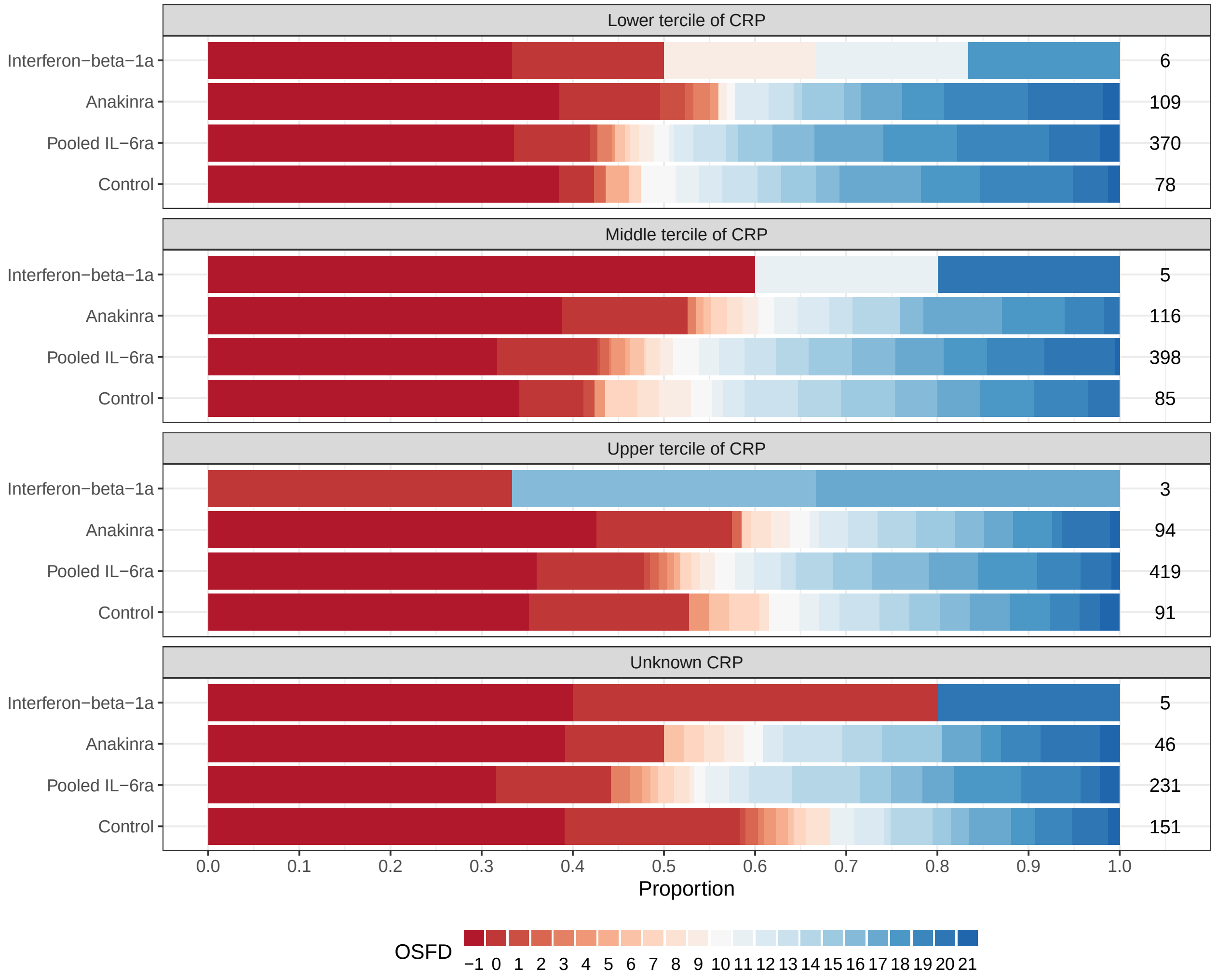

Figure 22: Empirical distribution of organ support free days (OSFD) for pooled IL-6ra and control interventions by baseline CRP subgroup. This plot is restricted to the Immune Modulation ITT population.

7.1.2 Subgroup analysis with differential effect on OSFD by baseline CRP subgroup

- Model: Primary analysis ordinal model
- Factors: Age, sex, site, time, Immune Modulation Therapy Domain interventions (control, interferon-beta-1a, anakinra, tocilizumab, and sarilumab), COVID-19 Antiviral domain interventions (no antiviral, lopinavir−ritonavir, hydroxychloroquine, combination therapy), Corticosteroid domain interventions (no corticosteroid, fixed-duration corticosteroids, shock-dependent corticosteroids), Immunoglobulin domain interventions (no convalescent plasma, convalescent plasma, delayed convalescent plasma), and Anticoagulation interventions (usual care thromboprophylaxis, therapeutic anticoagulation)

- Treatment effects for tocilizumab and sarilumab are modeled with independent priors.
- Differential treatment effects are modeled by baseline CRP tercile (low/middle/high/unknown). Due to small sample sizes, a differential effect by CRP subgroup is not estimated for Interferon-beta-1a.

Table 78: Odds ratio parameters for subgroup analysis with differential effect on OSFD by baseline CRP subgroup

	Mean	SD	Median	CrI
Interferon-beta-1a	1.24	0.52	1.14	(0.52, 2.47)
Anakinra, lower CRP tercile	1.04	0.25	1.01	(0.64, 1.60)
Anakinra, middle CRP tercile	0.92	0.20	0.90	(0.58, 1.38)
Anakinra, upper CRP tercile	1.34	0.32	1.30	(0.83, 2.05)
Anakinra, unknown CRP	0.94	0.29	0.90	(0.50, 1.64)
Tocilizumab, lower CRP tercile	1.30	0.23	1.28	(0.91, 1.82)
Tocilizumab, middle CRP tercile	1.33	0.23	1.31	(0.95, 1.83)
Tocilizumab, upper CRP tercile	1.90	0.32	1.87	(1.35, 2.59)
Tocilizumab, unknown CRP	1.43	0.25	1.41	(1.00, 1.99)
Sarilumab, lower CRP tercile	1.42	0.30	1.39	(0.93, 2.09)
Sarilumab, middle CRP tercile	1.37	0.28	1.34	(0.91, 2.01)
Sarilumab, upper CRP tercile	1.88	0.38	1.85	(1.24, 2.69)
Sarilumab, unknown CRP	1.69	0.45	1.63	(0.97, 2.71)
Age<39	4.24	0.52	4.21	(3.31, 5.34)
Age 40-49	2.42	0.24	2.41	(1.99, 2.93)
Age 50-59	1.84	0.15	1.83	(1.56, 2.14)
Age 70-79	0.52	0.05	0.51	(0.43, 0.62)
Age 80+	0.31	0.05	0.30	(0.22, 0.43)
Female	1.15	0.07	1.15	(1.01, 1.30)
Middle tercile CRP subgroup (relative to low)	0.96	0.12	0.95	(0.74, 1.22)
Upper tercile CRP subgroup (relative to low)	0.55	0.07	0.55	(0.42, 0.70)
Unknown CRP subgroup (relative to low)	0.68	0.10	0.68	(0.51, 0.90)

Table 79: Posterior probabilities for subgroup analysis with differential effect on OSFD by baseline CRP subgroup

	Posterior Probability
Interferon-beta-1a is superior to control	0.627
Interferon-beta-1a is futile	0.548
Anakinra is superior to control in lower CRP tercile	0.525
Anakinra is futile in lower CRP tercile	0.767
Anakinra is superior to control in middle CRP tercile	0.310
Anakinra is futile in middle CRP tercile	0.910
Anakinra is superior to control in upper CRP tercile	0.871
Anakinra is futile in upper CRP tercile	0.359
Anakinra is superior to control with unknown CRP	0.364

Table 79: Posterior probabilities for subgroup analysis with differential effect on OSFD by baseline CRP subgroup *(continued)*

	Posterior Probability
Anakinra is futile with unknown CRP	0.833
Tocilizumab is superior to control in lower CRP tercile	0.924
Tocilizumab is futile in lower CRP tercile	0.349
Tocilizumab is superior to control in middle CRP tercile	0.948
Tocilizumab is futile in middle CRP tercile	0.304
Tocilizumab is superior to control in upper CRP tercile	1.000
Tocilizumab is futile in upper CRP tercile	0.004
Tocilizumab is superior to control with unknown CRP	0.975
Tocilizumab is futile with unknown CRP	0.185
Sarilumab is superior to control in lower CRP tercile	0.944
Sarilumab is futile in lower CRP tercile	0.232
Sarilumab is superior to control in middle CRP tercile	0.933
Sarilumab is futile in middle CRP tercile	0.288
Sarilumab is superior to control in upper CRP tercile	0.998
Sarilumab is futile in upper CRP tercile	0.017
Sarilumab is superior to control with unknown CRP	0.966
Sarilumab is futile with unknown CRP	0.122

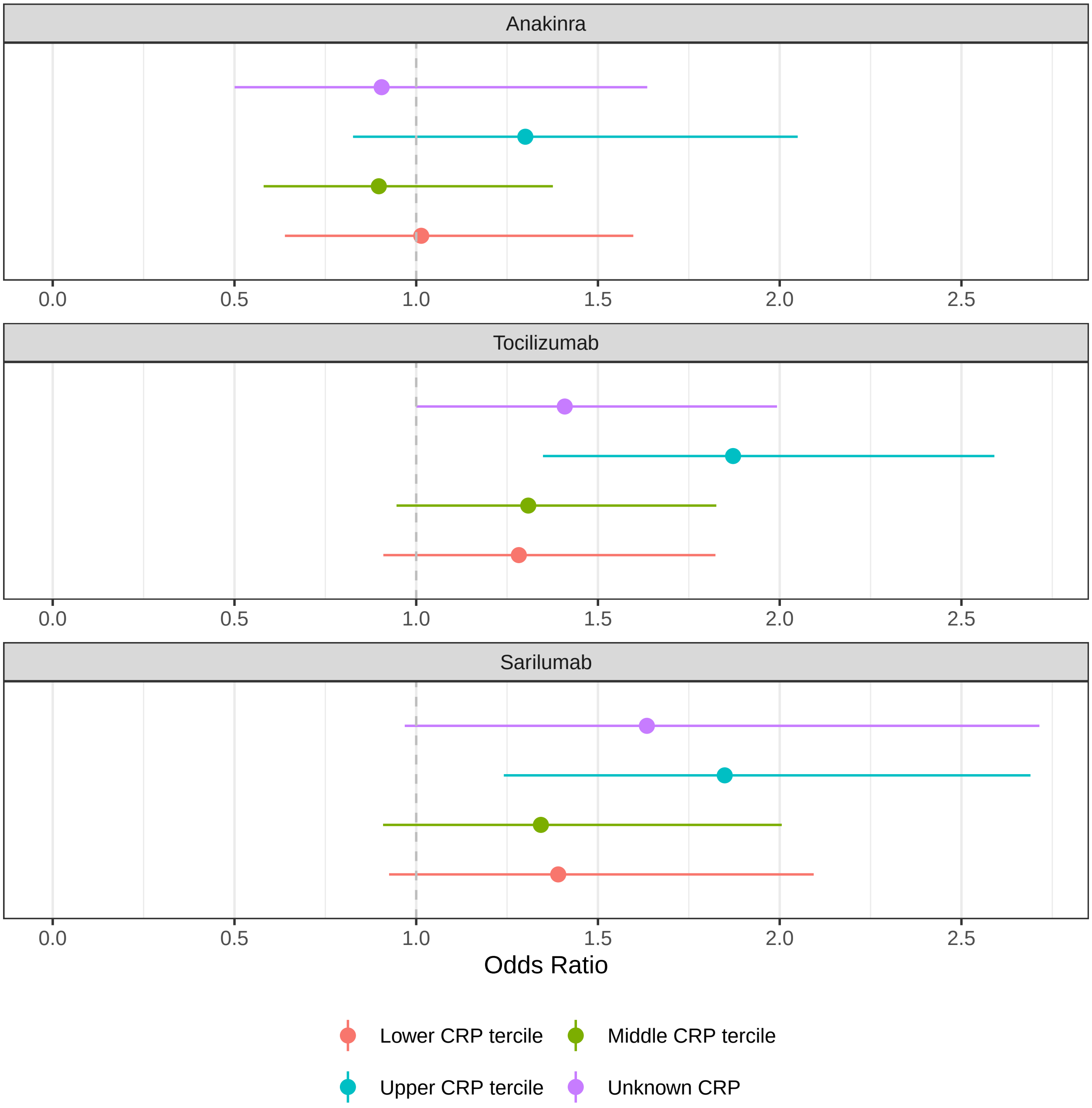

Figure 23: Estimated OSFD odds ratios for anakinra, tocilizumab, and sarilumab by CRP subgroup.

7.1.3 Subgroup analysis with differential effect on hospital survival by baseline CRP subgroup

- Model: Primary dichotomous model
- Factors: Age, sex, site, time, Immune Modulation Therapy Domain interventions (control, interferon-beta-1a, anakinra, tocilizumab, and sarilumab), COVID-19 Antiviral domain interventions (no antiviral,

lopinavir–ritonavir, hydroxychloroquine, combination therapy), Corticosteroid domain interventions (no corticosteroid, fixed-duration corticosteroids, shock-dependent corticosteroids), Immunoglobulin domain interventions (no convalescent plasma, convalescent plasma, delayed convalescent plasma), and Anticoagulation interventions (usual care thromboprophylaxis, therapeutic anticoagulation)

- Treatment effects for tocilizumab and sarilumab are modeled with independent priors.
- Differential treatment effects are modeled by baseline CRP tercile (low/middle/high/unknown). Due to small sample sizes, a differential effect by CRP subgroup is not estimated for Interferon-beta-1a.

Table 80: Odds ratio parameters for subgroup analysis with differential effect on hospital survival by baseline CRP subgroup

	Mean	SD	Median	CrI
Interferon-beta-1a	1.32	0.70	1.17	(0.46, 3.10)
Anakinra, lower CRP tercile	0.85	0.24	0.82	(0.48, 1.41)
Anakinra, middle CRP tercile	0.99	0.27	0.95	(0.57, 1.62)
Anakinra, upper CRP tercile	1.23	0.35	1.18	(0.68, 2.06)
Anakinra, unknown CRP	0.94	0.36	0.89	(0.43, 1.80)
Tocilizumab, lower CRP tercile	1.09	0.24	1.07	(0.70, 1.63)
Tocilizumab, middle CRP tercile	1.32	0.29	1.29	(0.85, 1.95)
Tocilizumab, upper CRP tercile	1.80	0.38	1.76	(1.18, 2.65)
Tocilizumab, unknown CRP	1.45	0.34	1.41	(0.90, 2.21)
Sarilumab, lower CRP tercile	1.33	0.35	1.29	(0.79, 2.15)
Sarilumab, middle CRP tercile	1.38	0.36	1.34	(0.81, 2.20)
Sarilumab, upper CRP tercile	1.86	0.47	1.80	(1.11, 2.95)
Sarilumab, unknown CRP	1.76	0.60	1.66	(0.89, 3.23)
Age<39	8.51	1.92	8.25	(5.48, 12.90)
Age 40-49	3.56	0.50	3.52	(2.69, 4.65)
Age 50-59	2.47	0.26	2.45	(2.00, 3.02)
Age 70-79	0.47	0.05	0.47	(0.38, 0.57)
Age 80+	0.26	0.05	0.26	(0.18, 0.37)
Female	1.26	0.11	1.26	(1.07, 1.48)
Middle tercile CRP subgroup (relative to low)	1.09	0.17	1.08	(0.79, 1.48)
Upper tercile CRP subgroup (relative to low)	0.66	0.10	0.65	(0.48, 0.87)
Unknown CRP subgroup (relative to low)	0.79	0.14	0.78	(0.56, 1.10)

Table 81: Posterior probabilities for subgroup analysis with differential effect on hospital survival by baseline CRP subgroup

	Posterior Probability
Interferon-beta-1a is superior to control	0.631
Interferon-beta-1a is futile	0.519
Anakinra is superior to control in lower CRP tercile	0.236

Table 81: Posterior probabilities for subgroup analysis with differential effect on hospital survival by baseline CRP subgroup *(continued)*

	Posterior Probability
Anakinra is futile in lower CRP tercile	0.915
Anakinra is superior to control in middle CRP tercile	0.433
Anakinra is futile in middle CRP tercile	0.793
Anakinra is superior to control in upper CRP tercile	0.728
Anakinra is futile in upper CRP tercile	0.520
Anakinra is superior to control with unknown CRP	0.365
Anakinra is futile with unknown CRP	0.802
Tocilizumab is superior to control in lower CRP tercile	0.624
Tocilizumab is futile in lower CRP tercile	0.706
Tocilizumab is superior to control in middle CRP tercile	0.876
Tocilizumab is futile in middle CRP tercile	0.373
Tocilizumab is superior to control in upper CRP tercile	0.997
Tocilizumab is futile in upper CRP tercile	0.030
Tocilizumab is superior to control with unknown CRP	0.935
Tocilizumab is futile with unknown CRP	0.238
Sarilumab is superior to control in lower CRP tercile	0.840
Sarilumab is futile in lower CRP tercile	0.386
Sarilumab is superior to control in middle CRP tercile	0.871
Sarilumab is futile in middle CRP tercile	0.329
Sarilumab is superior to control in upper CRP tercile	0.991
Sarilumab is futile in upper CRP tercile	0.049
Sarilumab is superior to control with unknown CRP	0.942
Sarilumab is futile with unknown CRP	0.158

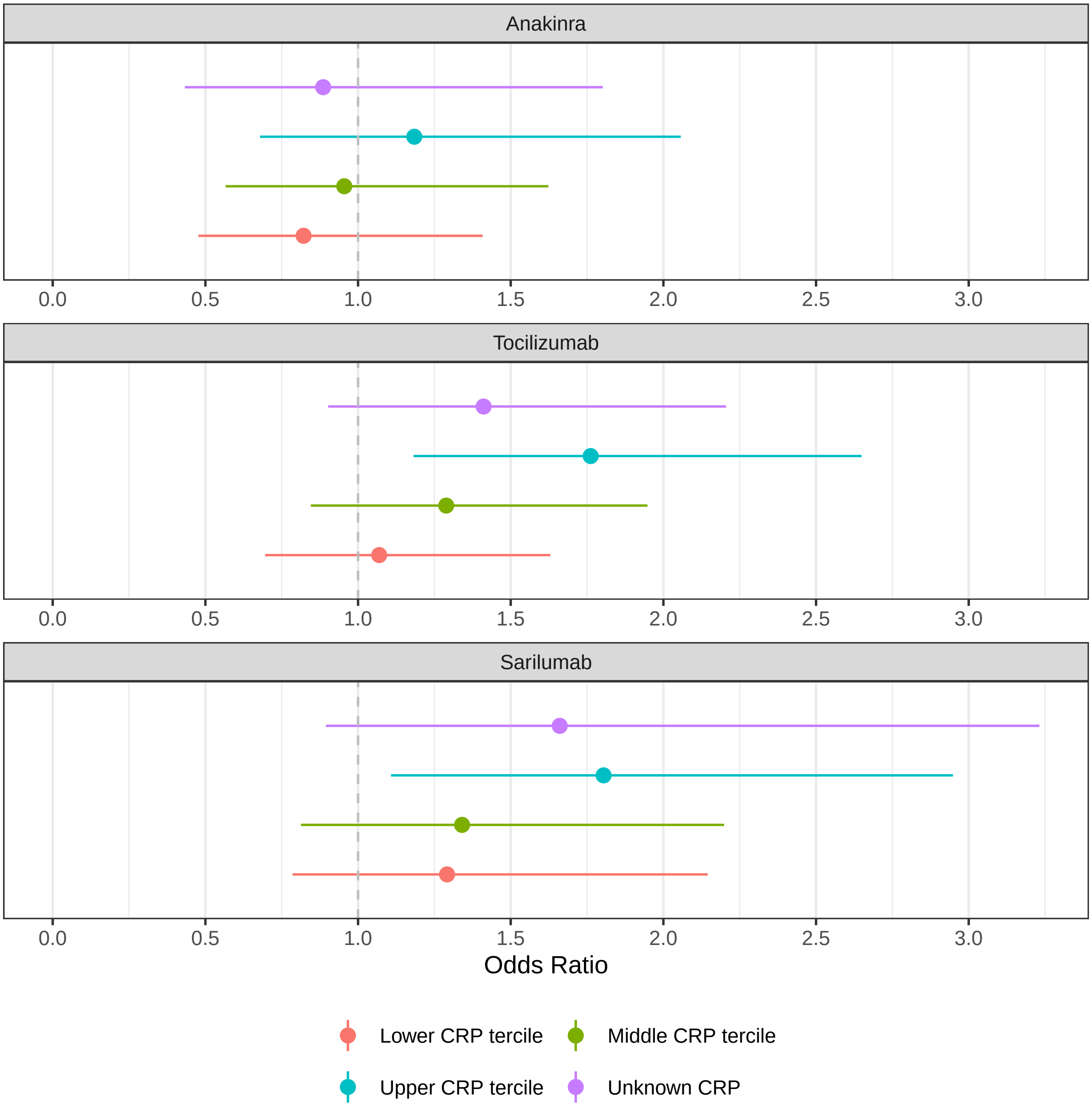

Figure 24: Estimated hospital survival odds ratios for anakinra, tocilizumab, and sarilumab by CRP subgroup.

7.2 Subgroup analyses by baseline mechanical ventilation status

7.2.1 Data summaries of baseline mechanical ventilation status in the Immune Modulation ITT population

Table 82: Summary of baseline invasive mechanical ventilation by intervention

Intervention	Patients	No IMV at baseline	IMV at baseline
Interferon-beta-1a	19	12 (63.2%)	7 (36.8%)
Anakinra	373	235 (63%)	138 (37%)
Tocilizumab	947	628 (66.3%)	319 (33.7%)
Sarilumab	482	336 (69.7%)	146 (30.3%)
Control	405	282 (69.6%)	123 (30.4%)
Pooled IL-6ra	1429	964 (67.5%)	465 (32.5%)
Overall	2226	1493 (67.1%)	733 (32.9%)

Table 83: Summary of subjects by baseline mechanical ventilation status in the Immune Modulation ITT population

Intervention	Patients	Known	Deaths (%)	OSFD median (IQR)	OSFD in survivors* median (IQR)
**No invasive mechanical ventilation at baseline**					
Interferon-beta-1a	12	12	3 (25%)	10 (-0.25, 17.25)	16 (9, 18)
Anakinra	235	230	76 (33%)	10 (-1, 17)	16 (10, 18)
Tocilizumab	628	621	175 (28.2%)	13 (-1, 18)	16 (11, 19)
Sarilumab	336	335	103 (30.7%)	13 (-1, 17)	16 (12, 18)
Control	282	282	90 (31.9%)	7 (-1, 16.75)	14 (6.75, 18)
Pooled IL-6ra	964	956	278 (29.1%)	13 (-1, 18)	16 (12, 18)
**Invasive mechanical ventilation at baseline**					
Interferon-beta-1a	7	7	4 (57.1%)	-1 (-1, 0)	0 (0, 5.5)
Anakinra	138	135	69 (51.1%)	-1 (-1, 7.5)	8 (0, 13)
Tocilizumab	319	317	142 (44.8%)	0 (-1, 11)	10 (0, 14)
Sarilumab	146	145	54 (37.2%)	0 (-1, 12)	9 (0, 15)
Control	123	123	60 (48.8%)	0 (-1, 9.5)	9 (0, 14)
Pooled IL-6ra	465	462	196 (42.4%)	0 (-1, 11)	9.5 (0, 15)

* Days free of organ support in survivors within 21 days

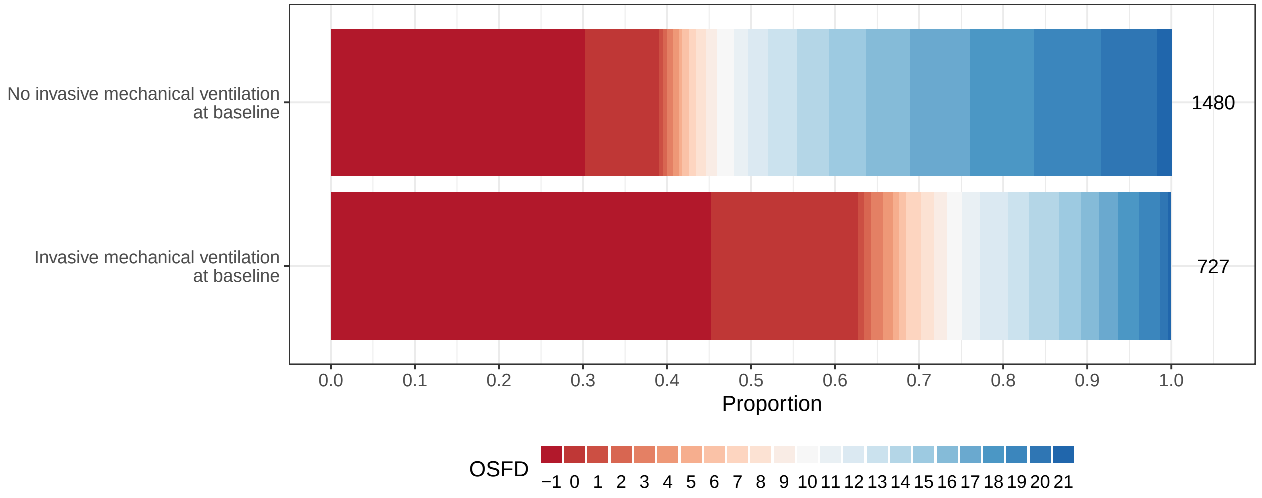

Figure 25: Empirical distribution of organ support free days (OSFD) by baseline invasive mechanical ventilation status. This plot is restricted to the Immune Modulation ITT population.

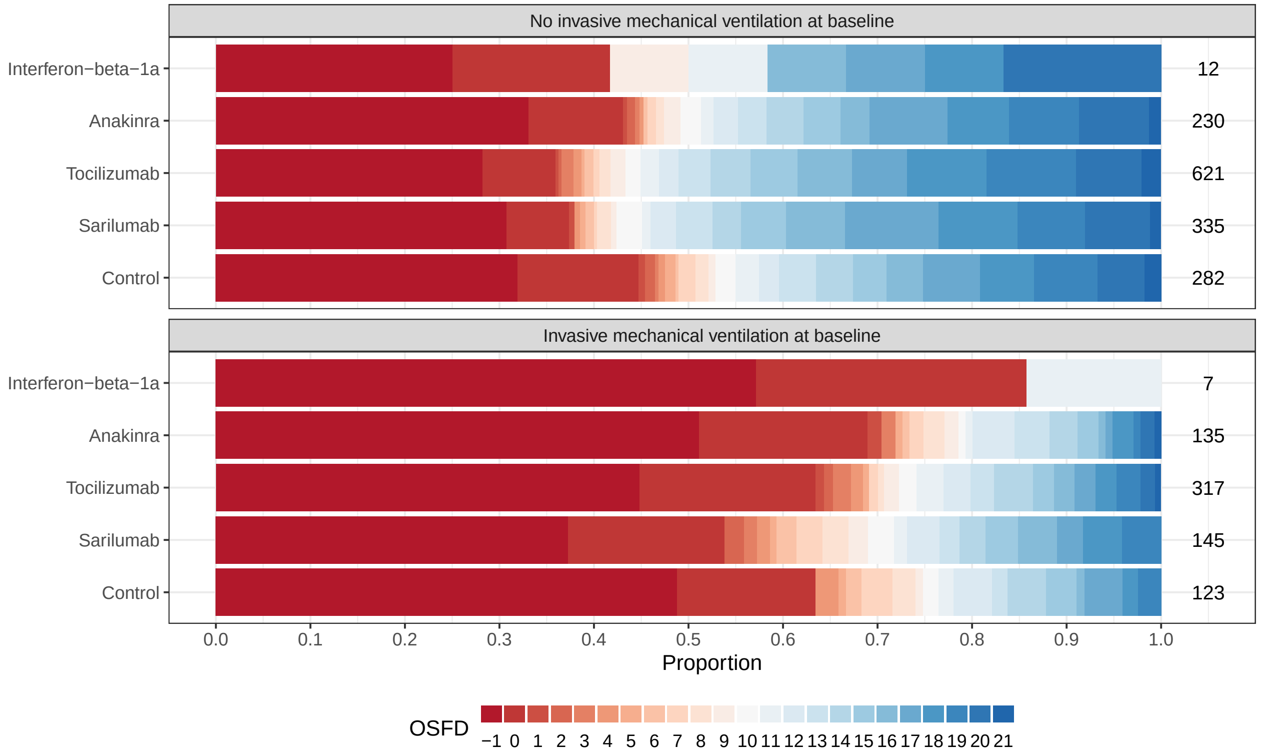

Figure 26: Empirical distribution of organ support free days (OSFD) for interferon-beta-1a, anakinra, tocilizumab, sarilumab, and control by baseline invasive mechanical ventilation status. This plot is restricted to the Immune Modulation ITT population.

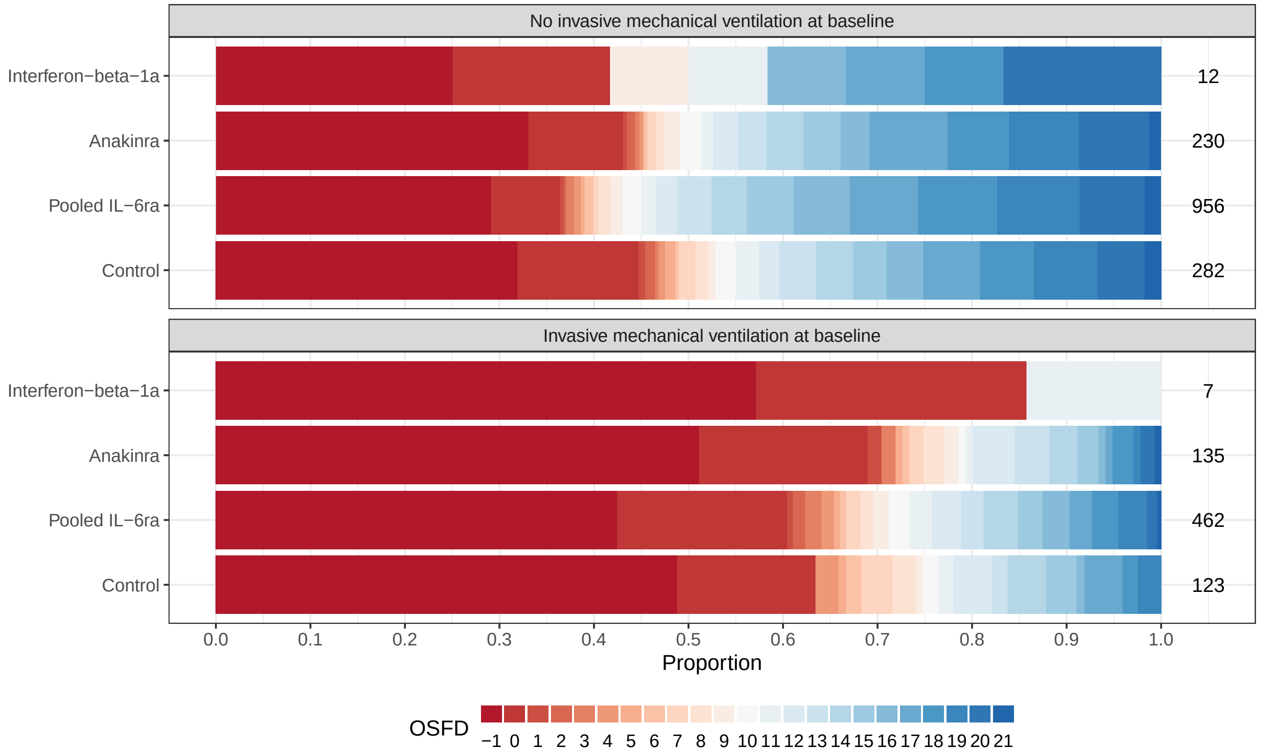

Figure 27: Empirical distribution of organ support free days (OSFD) for pooled IL-6ra and control interventions by baseline invasive mechanical ventilation status. This plot is restricted to the Immune Modulation ITT population.

7.2.2 Subgroup analysis with differential effect on OSFD by baseline mechanical ventilation status

- Model: Primary analysis ordinal model
- Factors: Age, sex, site, time, Immune Modulation Therapy Domain interventions (control, interferon-beta-1a, anakinra, tocilizumab, and sarilumab), COVID-19 Antiviral domain interventions (no antiviral, lopinavir−ritonavir, hydroxychloroquine, combination therapy), Corticosteroid domain interventions (no corticosteroid, fixed-duration corticosteroids, shock-dependent corticosteroids), Immunoglobulin domain interventions (no convalescent plasma, convalescent plasma, delayed convalescent plasma), and Anticoagulation interventions (usual care thromboprophylaxis, therapeutic anticoagulation)
- Differential treatment effects for immune modulation interventions by baseline invasive mechanical ventilation status will be compared to the control arm. A posterior probability of superiority of 99% will be used as a statistical trigger for efficacy for each subgroup.
- Population: Unblinded ITT

Table 84: Odds ratio parameters for subgroup analysis with differential effect on OSFD by mechanical ventilation

	Mean	SD	Median	CrI
Interferon-beta-1a, no IMV	1.87	0.95	1.67	(0.66, 4.24)
Interferon-beta-1a, IMV	0.83	0.53	0.70	(0.21, 2.24)
Anakinra, no IMV	1.11	0.19	1.09	(0.78, 1.53)
Anakinra, IMV	0.89	0.19	0.87	(0.57, 1.31)
Tocilizumab, no IMV	1.62	0.20	1.61	(1.26, 2.07)
Tocilizumab, IMV	1.22	0.19	1.20	(0.88, 1.64)
Sarilumab, no IMV	1.56	0.23	1.54	(1.16, 2.08)
Sarilumab, IMV	1.40	0.28	1.38	(0.94, 2.02)
Age<39	4.39	0.54	4.36	(3.43, 5.53)
Age 40-49	2.29	0.22	2.28	(1.88, 2.76)
Age 50-59	1.83	0.15	1.82	(1.56, 2.13)
Age 70-79	0.51	0.05	0.50	(0.42, 0.60)
Age 80+	0.28	0.05	0.27	(0.19, 0.38)
Female	1.20	0.08	1.20	(1.06, 1.36)
Mechanical ventilation (relative no IMV)	0.41	0.04	0.41	(0.34, 0.48)

Table 85: Posterior probabilities for subgroup analysis with differential effect on OSFD by mechanical ventilation with antiviral interventions

	Posterior Probability
Interferon-beta-1a is superior to control with no IMV	0.861
Interferon-beta-1a is futile with no IMV	0.247
Interferon-beta-1a is superior to control with IMV	0.274
Interferon-beta-1a is futile with IMV	0.821
Anakinra is superior to control with no IMV	0.695
Anakinra is futile with no IMV	0.710
Anakinra is superior to control with IMV	0.254
Anakinra is futile with IMV	0.941
Tocilizumab is superior to control with no IMV	1.000
Tocilizumab is futile with no IMV	0.009
Tocilizumab is superior to control with IMV	0.876
Tocilizumab is futile with IMV	0.495
Sarilumab is superior to control with no IMV	0.999
Sarilumab is futile with no IMV	0.045
Sarilumab is superior to control with IMV	0.948
Sarilumab is futile with IMV	0.240

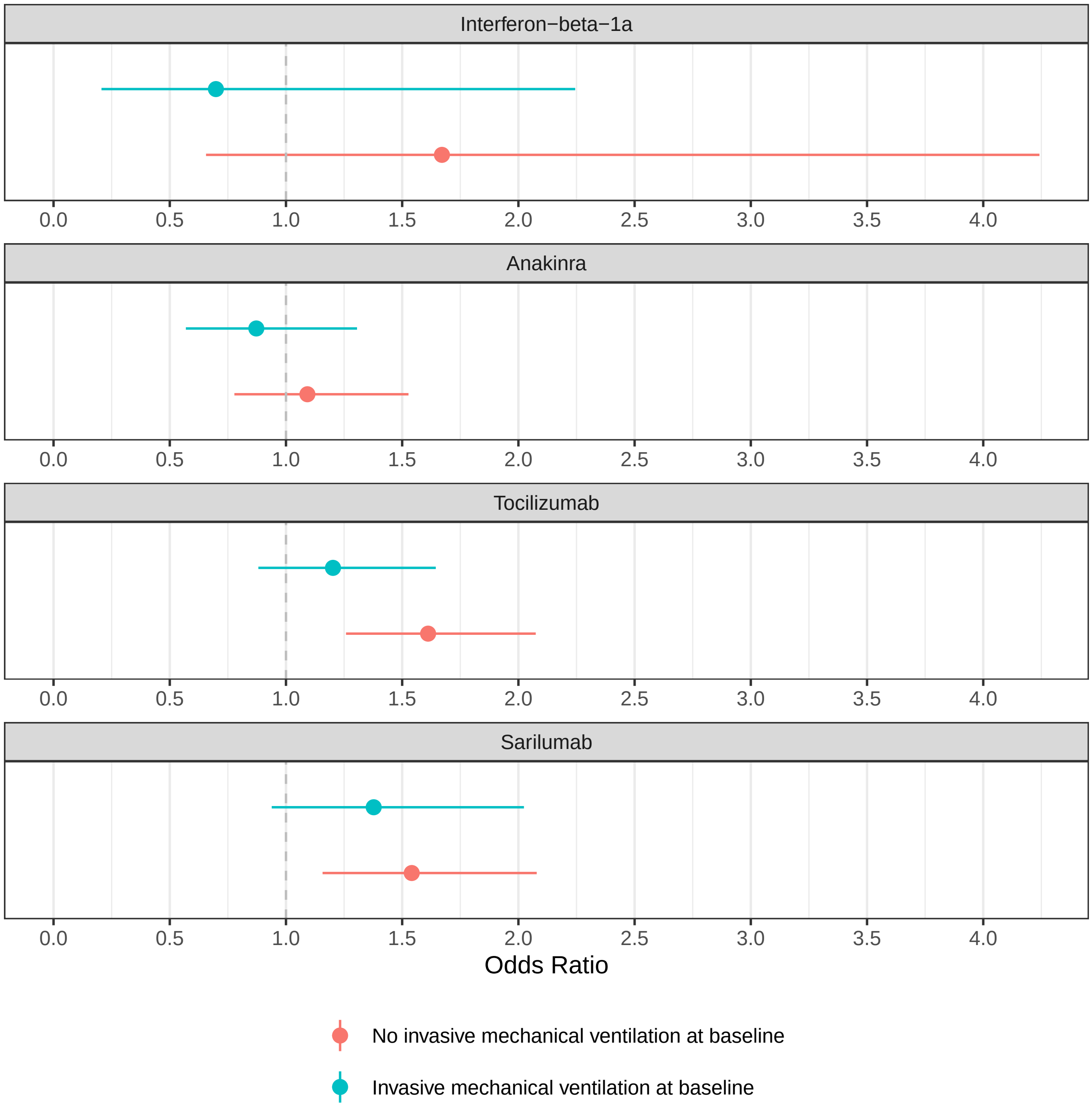

Figure 28: Estimated OSFD odds ratios for interferon, anakinra, tocilizumab, and sarilumab by baseline mechanical ventilation subgroup.

7.2.3 Subgroup analysis with differential effect on hospital survival by baseline mechanical ventilation status

- Model: Primary dichotomous model

- Factors: Age, sex, site, time, Immune Modulation Therapy Domain interventions (control, interferon-beta-1a, anakinra, tocilizumab, and sarilumab), COVID-19 Antiviral domain interventions (no antiviral, lopinavir–ritonavir, hydroxychloroquine, combination therapy), Corticosteroid domain interventions (no corticosteroid, fixed-duration corticosteroids, shock-dependent corticosteroids), Immunoglobulin domain interventions (no convalescent plasma, convalescent plasma, delayed convalescent plasma), and Anticoagulation interventions (usual care thromboprophylaxis, therapeutic anticoagulation)
- Differential treatment effects for immune modulation interventions by baseline invasive mechanical ventilation status will be compared to the control arm. A posterior probability of superiority of 99% will be used as a statistical trigger for efficacy for each subgroup.
- Population: Unblinded ITT

Table 86: Odds ratio parameters for subgroup analysis with differential effect on hospital survival by mechanical ventilation

	Mean	SD	Median	CrI
Interferon-beta-1a, no IMV	2.13	1.38	1.78	(0.59, 5.74)
Interferon-beta-1a, IMV	0.91	0.66	0.74	(0.21, 2.59)
Anakinra, no IMV	1.13	0.25	1.10	(0.72, 1.70)
Anakinra, IMV	0.84	0.22	0.81	(0.50, 1.34)
Tocilizumab, no IMV	1.56	0.25	1.54	(1.12, 2.11)
Tocilizumab, IMV	1.18	0.23	1.16	(0.79, 1.70)
Sarilumab, no IMV	1.51	0.29	1.48	(1.02, 2.16)
Sarilumab, IMV	1.57	0.39	1.53	(0.95, 2.45)
Age<39	8.65	1.95	8.41	(5.53, 13.16)
Age 40-49	3.42	0.47	3.38	(2.60, 4.45)
Age 50-59	2.44	0.25	2.43	(1.98, 2.96)
Age 70-79	0.46	0.05	0.46	(0.38, 0.56)
Age 80+	0.25	0.05	0.25	(0.17, 0.36)
Female	1.29	0.11	1.28	(1.09, 1.51)
Mechanical ventilation (relative no IMV)	0.59	0.07	0.59	(0.47, 0.74)

Table 87: Posterior probabilities for subgroup analysis with differential effect on hospital survival by mechanical ventilation with antiviral interventions

	Posterior Probability
Interferon-beta-1a is superior to control with no IMV	0.840
Interferon-beta-1a is futile with no IMV	0.250
Interferon-beta-1a is superior to control with IMV	0.325
Interferon-beta-1a is futile with IMV	0.768

Table 87: Posterior probabilities for subgroup analysis with differential effect on hospital survival by mechanical ventilation with antiviral interventions *(continued)*

	Posterior Probability
Anakinra is superior to control with no IMV	0.678
Anakinra is futile with no IMV	0.647
Anakinra is superior to control with IMV	0.208
Anakinra is futile with IMV	0.938
Tocilizumab is superior to control with no IMV	0.996
Tocilizumab is futile with no IMV	0.063
Tocilizumab is superior to control with IMV	0.768
Tocilizumab is futile with IMV	0.576
Sarilumab is superior to control with no IMV	0.980
Sarilumab is futile with no IMV	0.132
Sarilumab is superior to control with IMV	0.959
Sarilumab is futile with IMV	0.163

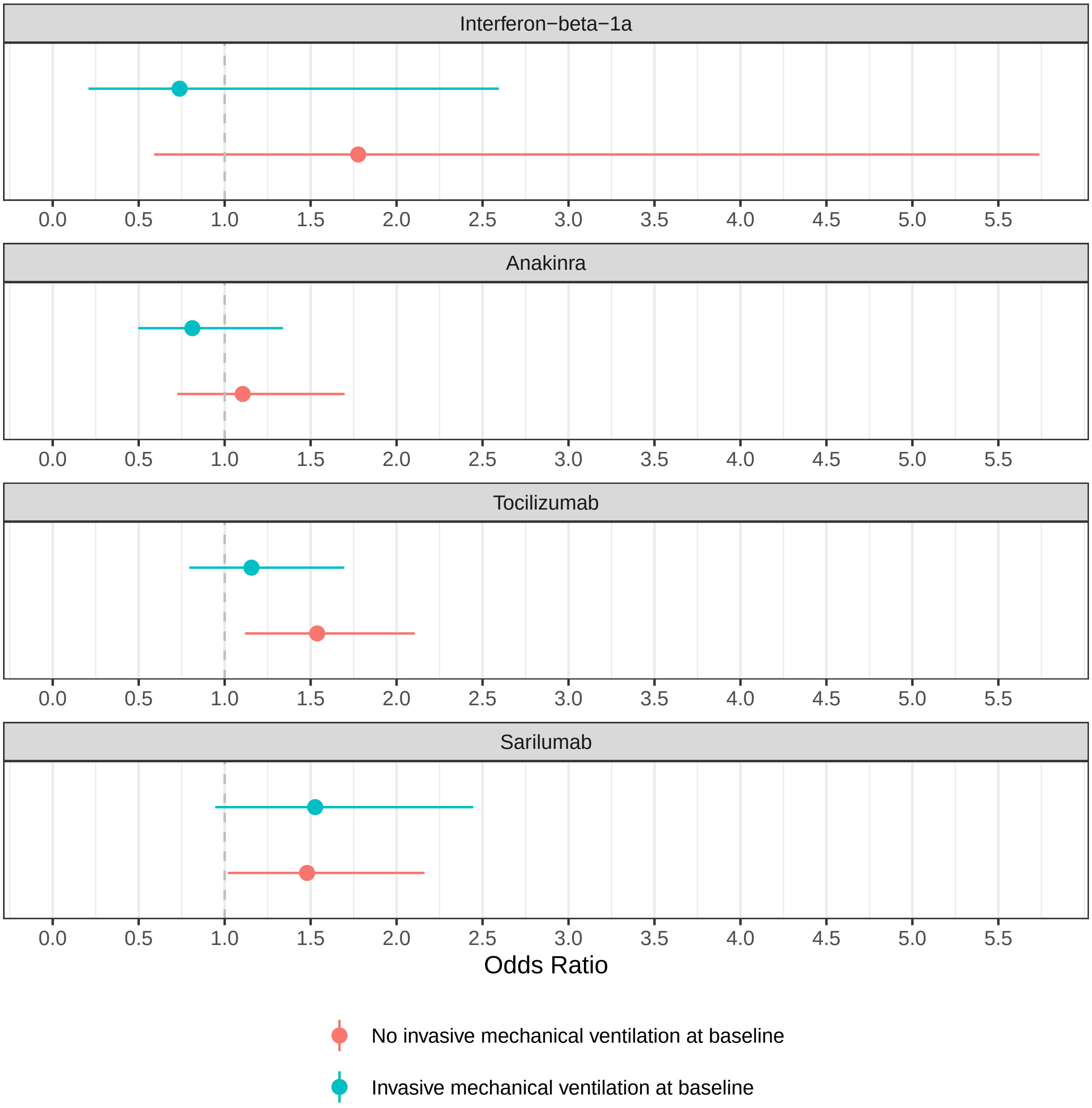

Figure 29: Estimated hospital survival odds ratios for interferon, anakinra, tocilizumab, and sarilumab by baseline mechanical ventilation subgroup.

8 Report production and data sources

All analyses in this report are based on the following documents:

- Statistical Analysis Appendix for REMAP-COVID, version 1, dated August 18, 2020;
- Statistical Analysis Plan for the Immune Modulation Therapy Domain for Patients with COVID-19 Pandemic Infection Suspected Or Proven (PISOP), version 1.1, dated April 15, 2021;
- Statistical Analysis Plan for the Tocilizumab Analysis of the Immune Modulation Therapy Domain for Patients with COVID-19 Pandemic Infection Suspected Or Proven (PISOP); version 1.0, dated November 25, 2020;
- Current State of the Statistical Model: Pandemic Model, version 3.1-IM, dated May 5, 2021;
- Addendum to the Immune Modulation PISOP SAP and Current State Documents, dated May 5, 2021

The blinded ITSC analysis team at Berry Consultants performed the analyses in this report using data received from multiple sources. The OSFD outcomes and treatment assignments for patients in unblinded domains were sent from the unblinded Statistical Analysis Committee (SAC) with all blinded information removed. The baseline, discharge and daily data for patients randomized in unblinded domains were sent from an unblinded data coordination team at Monash University. The table below shows the file names for the data exports from each data source and the date each file was received by the blinded ITSC analysis committee. All merging and summarization of data was done using the R statistical computing environment. This report was generated from an Rmarkdown document in the Rstudio software.

Table 88: Summary of data sources

Filename	Date received	Description
merged_REMAP_PISOPSevere_IM1_2021-05-11.csv	May 11, 2021	OSFD and treatment assignments from unblinded SAC
Immune modulation baseline discharge compliance data_May3_Designteam.csv	May 10, 2021	Baseline and discharge data for unblinded ITT
Immune Modulation daily data_May3_Designteam.csv	May 10, 2021	Daily ICU data for unblinded ITT
