# Supplementary material for: Tocilizumab, sarilumab and anakinra in critically ill patients with COVID-19: a randomised, controlled, open-label, adaptive platform trial
Source: Thorax. 2025 May 13;80(8):e222488. doi: 10.1136/thorax-2024-222488 (PMC12322455; doi:10.1136/thorax-2024-222488)
Supplement: online supplemental file 5 [file thorax-80-8-s005.pdf]

Domain-Specific Appendix: COVID-19 Immune Modulation Therapy

SUPERSEDED

REMAP-CAP: Randomized, Embedded, Multifactorial Adaptive Platform trial for Community-Acquired Pneumonia

COVID-19 Immune Modulation Therapy Domain-Specific Appendix Version 2.0 dated 07 April 2020

Summary

In this domain of the REMAP-CAP trial, participants meeting the platform entry criteria for REMAP-CAP admitted to participating intensive care units with suspected or microbiological testing-confirmed COVID-19 infection will be randomized to receive one of up to five interventions depending on availability and acceptability:

- No immune modulation for COVID-19 (no placebo)
- interferon-beta-1a (IFN-β1a)
- anakinra (interleukin-1 receptor antagonist; IL1Ra)
- tocilizumab (IL-6 receptor antagonist; IL6Ra)
- sarilumab (IL-6 receptor antagonist; IL6Ra)

This domain will enroll only patients only in the pandemic infection is suspected or proven (PISOP) stratum and be analyzed in the Pandemic Statistical Model as outlined from the Pandemic Appendix to Core (PAtC).

At this participating site the following interventions have been selected within this domain:

☐ No immune modulation for COVID-19 (no placebo)

☐ interferon-beta-1a (IFN-β1a)

☐ anakinra (interleukin-1 receptor antagonist; IL1Ra)

☐ tocilizumab (IL-6 receptor antagonist; IL6Ra)

☐ sarilumab (IL-6 receptor antagonist; IL6Ra)

SUPERSEDED

**REMAP-CAP: COVID-19 Immune Modulation Therapy Domain Summary**	
Interventions	• No immune modulation for COVID-19 (no placebo)<br>• Interferon-beta-1a (IFN-β1a)<br>• Anakinra (interleukin-1 receptor antagonist)<br>• Tocilizumab (interleukin-6 receptor antagonist)<br>• Sarilumab (interleukin-6 receptor antagonist)
Unit of Analysis and Strata	The default unit-of-analysis for this domain will be the pandemic infection suspected or confirmed (PISOP) stratum. Analysis and Response Adaptive Randomization are applied by PISOP stratum. Unit of analysis may be modified to allow analysis to be stratified by SARS-CoV-2 infection confirmed or not confirmed with borrowing permitted. If this occurs, Response Adaptive Randomization will be applied to patients in the PISOP stratum using probabilities derived from SARC-CoV-2 confirmed stratum. Unit of analysis may be further modified by application of a biomarker strata.
Evaluable treatment-by-treatment Interactions	Treatment-treatment interactions will be evaluated between interventions in this domain and interventions in the Corticosteroid Domain and with the COVID-19 Antiviral Therapy Domain. No other interactions will be evaluated with any other domain.
Nesting	There is one nest, comprising tocilizumab and sarilumab, which are both interleukin-6 inhibitors.
Timing of Reveal	Randomization with Immediate Reveal and Initiation or Randomization with Deferred Reveal if prospective agreement to participate is required.
Inclusions	Patients will be eligible for this domain if:<br>• COVID-19 infection is suspected by the treating clinician or has been confirmed by microbiological testing<br>• Microbiological testing for SARS-CoV-2 infection of upper or lower respiratory tract secretions or both has occurred or is intended to occur
Domain-Specific Exclusions	Patients will be excluded from this domain if they have any of the following:<br>• More than 24 hours has elapsed since ICU admission<br>• Patient has already received any dose of one or more of any form of interferon, anakinra, tocilizumab, or sarilumab during this hospitalization or is on long-term therapy with any of these agents prior to this hospital admission<br>• Known condition or treatment resulting in ongoing immune suppression including neutropenia prior to this hospitalization<br>• Patient has been randomized in a trial evaluating an immune modulation agent for proven or suspected COVID-19 infection, where the protocol of that trial requires ongoing administration of study drug<br>• The treating clinician believes that participation in the domain would not be in the best interests of the patient
Intervention-Specific Exclusions	• Known hypersensitivity to an agent specified as an intervention in this domain will exclude a patient from receiving that agent<br>• Intention to prescribe systemic corticosteroids for any reason, other than participation in the Corticosteroid Domain of this platform, will result in exclusion from receiving IFN-β1a<br>• Known hypersensitivity to proteins produced by E. coli will result in exclusion from receiving anakinra<br>• Known or suspected pregnancy will result in exclusion from the anakinra, IFN-β1a, tocilizumab and sarilumab interventions.<br>• A baseline alanine aminotransferase or an aspartate aminotransferase that is more than five times the upper limit of normal will result in exclusion from receiving tocilizumab or sarilumab<br>• A baseline platelet count < 50 x 10^9 / L will result in exclusion from receiving tocilizumab or sarilumab

SUPERSEDED

Outcome measures	Primary REMAP endpoint: as defined in an operational document specified from the Pandemic Appendix to the Core Protocol Section 7.5.1<br><br>Secondary REMAP endpoints: as defined in an operational document specified from Pandemic Appendix to the Core Protocol Section 7.5.2<br><br>Secondary domain-specific endpoints (during hospitalization censored 90 days from the date of enrollment):<br><br>• Serial detection of SARS-CoV-2 in upper or lower respiratory tract specimens (using only specimens collected for routine clinically indicated testing)<br><br>• Serious Adverse Events (SAE) as defined in Core Protocol

SUPERSEDED

TABLE OF CONTENTS

1. ABBREVIATIONS 8

2. PROTOCOL APPENDIX STRUCTURE 10

3. COVID-19 IMMUNE MODULATION THERAPY DOMAIN-SPECIFIC APPENDIX VERSION 11

3.1. Version history 11

4. COVID-19 IMMUNE MODULATION THERAPY DOMAIN GOVERNANCE 11

4.1. Domain members 11

4.2. Contact Details 12

4.3. COVID-19 Immune Modulation therapy Domain-Specific Working Group Authorization 13

5. BACKGROUND AND RATIONALE 13

5.1. Domain definition 13

5.2. Domain-specific background 13

5.2.1. COVID-19 infection 13

5.2.2. Clinical trials for COVID-19 infection 15

5.2.3. Intervention strategy for this domain 17

5.2.4. IFN-β1a 18

5.2.5. Anakinra 23

5.2.6. IL-6 receptor antagonists 25

6. DOMAIN OBJECTIVES 30

7. TRIAL DESIGN 31

7.1. Population 31

7.2. Eligibility criteria 31

7.2.1. Domain inclusion criteria 31

7.2.2. Domain exclusion criteria 31

7.2.3. Intervention exclusion criteria 32

7.3. Interventions 33

7.3.1. Immune modulation interventions 33

7.3.2. IFN-β1a 33

7.3.3. IL-1Ra 34

7.3.4. Tocilizumab 34

7.3.5. Sarilumab 34

7.3.6. Discontinuation of study drug 35

SUPERSEDED

7.3.7. COVID-19 immune modulation strategy in patients negative for COVID-19 infection 35
7.4. Concomitant care 35
7.5. Endpoints 35
7.5.1. Primary endpoint 35
7.5.2. Secondary endpoints 35
8. TRIAL CONDUCT 36
8.1. Microbiology 36
8.2. Domain-specific data collection 36
8.2.1. Clinical data collection 36
8.3. Criteria for discontinuation 37
8.4. Blinding 37
8.4.1. Blinding 37
8.4.2. Unblinding 37
9. STATISTICAL CONSIDERATIONS 37
9.1. Domain-specific stopping rules 37
9.2. Unit-of-analysis and strata 37
9.3. Timing of revealing of randomization status 38
9.4. Interactions with interventions in other domains 38
9.5. Nesting of interventions 39
9.6. Threshold probability for superiority and inferiority 40
9.7. Threshold odds ratio delta for equivalence 40
9.8. Informative priors 40
9.9. Post-trial sub-groups 40
10. ETHICAL CONSIDERATIONS 41
10.1. Data Safety and Monitoring Board 41
10.2. Potential domain-specific adverse events 41
10.3. Domain-specific consent issues 42
11. GOVERNANCE ISSUES 42
11.1. Funding of domain 42
11.2. Funding of domain interventions and outcome measures 43
11.3. Domain-specific declarations of interest 43
12. REFERENCES 44

SUPERSEDED

13. APPENDIX 1. OVERVIEW OF DESIGN AND INITIAL RESULTS FOR IMMUNE MODULATION THERAPY COVID-19 DOMAIN AND CORTICOSTEROID DOMAIN 49
13.1. Introduction 49
13.1.1. Treatment Arms 49
13.1.2. Primary Endpoint 49
13.2. Primary Analysis Model 50
13.2.1. Model Specification 50
13.3. Study Design 51
13.3.1. Timing of Interim Analyses 51
13.3.2. Allocation 52
13.3.3. Arm Dropping Rules for Futility 52
13.3.4. Final Success Rule 52
13.4. Simulation Details 52
13.4.1. Control rate assumptions 53
13.4.2. Treatment Effects 54
13.5. Operating Characteristics 55
13.6. Summary 56

SUPERSEDED

1. ABBREVIATIONS

AE	Adverse Event
ALT	Alanine Aminotransferase
ARDS	Acute Respiratory Distress Syndrome
CCP	Clinical Characterization Protocol
CRS	Cytokine Release Syndrome
DSA	Domain-Specific Appendix
DMSB	Data Safety and Monitoring Board
DSWG	Domain-Specific Working Group
FDA	U.S. Food and Drug Administration
ICU	Intensive Care Unit
IFN	Interferon
INTEREST trial	Efficacy and Safety of FP-1201-lyo (Interferon Beta-1a) in Patients Having Acute Respiratory Distress Syndrome (ARDS)
IL	Interleukin
ISIG	International Statistics Interest Group
ITSC	International Trial Steering Committee
LFT	Liver function tests
MAS	Macrophage Activation Syndrome
MERS-CoV	Middle East respiratory syndrome coronavirus
MIRACLE trial	The MERS-CoV Infection tReated With A Combination of Lopinavir/Ritonavir and IntErferon-β1b trial
MMF	Mycophenolate mofetil
OTD	Optimal Tolerated Dose
PAtC	Pandemic Appendix to the Core Protocol
PISOP	Pandemic infection is suspected or proven

SUPERSEDED

PY	Patient years
REMAP-CAP	Randomized, Embedded, Multifactorial, Adaptive Platform trial for Community-Acquired Pneumonia
RO	Receptor Occupancy
RSA	Region-Specific Appendix
SAE	Serious Adverse Event
SARS	Serious Acute Respiratory Syndrome
SOBI	Swedish Orphan Biovitrum
WHO	World Health Organization

SUPERSEDED

2. PROTOCOL APPENDIX STRUCTURE

The structure of this protocol is different to that used for conventional trials because this trial is highly adaptive and the description of these adaptations is better understood and specified using a 'modular' protocol design. While all adaptations are pre-specified, the structure of the protocol is designed to allow the trial to evolve over time, for example by the introduction of new domains or interventions or both (see glossary, Section 1.2 Core Protocol for definitions of these terms) and commencement of the trial in new geographical regions.

The protocol has multiple modules, in brief, comprising a Core Protocol (overview and design features of the study); a Statistical Analysis Appendix (details of the current statistical analysis plan and models); Simulations Appendix (details of the current simulations of the REMAP); multiple Domain-Specific Appendices (DSA) (detailing all interventions currently being studied in each domain); and multiple Region-Specific Appendices (RSA) (detailing regional management and governance).

The Core Protocol contains all information that is generic to the trial, irrespective of the regional location in which the trial is conducted and the domains or interventions that are being tested. The Core Protocol may be amended but it is anticipated that such amendments will be infrequent.

The Core Protocol does not contain information about the intervention(s), within each domain, because one of the trial adaptations is that domains and interventions will change over time. Information about interventions within each domain is covered in a DSA. These Appendices are anticipated to change over time, with removal and addition of options within an existing domain, at one level, and removal and addition of entire domains, at another level. Each modification to a DSA will be subject to a separate ethics application for approval.

The Core Protocol does not contain detailed information about the statistical analysis or simulations, because the analytic model will also change over time in accordance with the domain and intervention trial adaptations but this information is contained in the Statistical Analysis and Simulations Appendices. These Appendices are anticipated to change over time, as trial adaptations occur. Each modification will be subject to approval from the International Trial Steering Committee (ITSC) in conjunction with advice from the International Statistics Interest Group (ISIG) and the Data Safety and Monitoring Board (DSMB).

The Core Protocol also does not contain information that is specific to a particular region in which the trial is conducted, as the locations that participate in the trial are also anticipated to increase

SUPERSEDED

over time. Information that is specific to each region that conducts the trial is contained within a RSA. This includes information related to local management, governance, and ethical and regulatory aspects. It is planned that, within each region, only that region's RSA, and any subsequent modifications, will be submitted for ethical review in that region.

The current version of the Core Protocol, DSAs, RSAs and the Statistical Analysis Appendix is listed in the Protocol Summary and on the study website (www.remapcap.org).

3. COVID-19 IMMUNE MODULATION THERAPY DOMAIN-SPECIFIC APPENDIX VERSION

The version of the COVID-19 Immune Modulation Therapy Domain-Specific Appendix is in this document's header and on the cover page.

3.1. *Version history*

Version 1: Approved by the COVID-19 Domain-Specific Working Group (DSWG) on 11 March, 2020.

Version 2: Approved by the COVID-19 DSWG on 07 April, 2020.

4. COVID-19 IMMUNE MODULATION THERAPY DOMAIN GOVERNANCE

4.1. *Domain members*

Chair (Immune Modulation Domain):

Dr Lennie Derde

Deputy Chair (Immune Modulation Domain):

Professor Ville Pettilä

Members:

Professor Derek Angus

Dr Kenneth Baillie

Professor Richard Beasley

A/Prof Scott Berry

SUPERSEDED

Professor Marc Bonten

Professor Frank Brunkhorst

Professor Allen Cheng

Dr Nichole Cooper

Professor Menno de Jong

Dr Lennie Derde

Dr Rob Fowler

Mr James Galea

Professor Herman Goossens

Professor Anthony Gordon

Dr Thomas Hills

Mr Cameron Green

Professor Andrew King

Dr Ed Litton

Professor John Marshall

Dr Colin McArthur

Dr Susan Morpeth

Dr Srinivas Murthy

Dr Mihai Netea

Professor Alistair Nichol

Dr Kayode Ogungbenro

A/Prof Rachael Parke

Ms Jane Parker

Professor Kathy Rowan

Dr Steve Tong

Dr Tim Uyeki

Dr Frank van de Veerdonk

Professor Steve Webb

Dr Taryn Youngstein

4.2. *Contact Details*

Chair: Dr Lennie Derde

SUPERSEDED

Julius Center for Health Sciences and Primary Care

University Medical Center Utrecht

Heidelberglaan 100

3508 GA, Utrecht

THE NETHERLANDS

Phone: +31 88 755 5555

Email: L.P.G.Derde@umcutrecht.nl

4.3. *COVID-19 Immune Modulation therapy Domain-Specific Working Group Authorization*

The COVID-19 Domain-Specific Working Group have read the appendix and authorize it as the official COVID-19 Immune Modulation Therapy Domain-Specific Appendix for the study entitled REMAP-CAP. Signed on behalf of the committee,

Chair ______________________ Date 07 April 2020

Dr Lennie Derde

SUPERSEDED

5. BACKGROUND AND RATIONALE

5.1. *Domain definition*

This is a domain within the REMAP-CAP to test the effectiveness of different strategies for immune mediation for suspected or microbiological testing-confirmed COVID-19 in patients with concomitant severe pneumonia who are admitted to an Intensive Care Unit (ICU).

It should be noted that the Corticosteroid Domain represents an alternative approach to immune modulation, and this domain will also be active for patients with suspected or microbiological testing-confirmed COVID-19.

5.2. *Domain-specific background*

5.2.1. COVID-19 infection

The first report of infection with COVID-19 occurred in Wuhan, China, in late 2019. Since that time, and as of the time of writing of this DSA, there have been tens of thousands of reported cases across the region with a range of severity, several thousand deaths and documented sustained human-

human transmission. On January 30th 2020, the World Health Organization (WHO) declared this outbreak a Public Health Emergency of International Concern (https://www.who.int/news-room/detail/30-01-2020-statement-on-the-second-meeting-of-the-international-health-regulations-(2005)-emergency-committee-regarding-the-outbreak-of-novel-coronavirus-(2019-ncov)). Given past history with novel coronaviruses, such as Severe Acute Respiratory Syndrome (SARS) and Middle East respiratory syndrome coronavirus (MERS-CoV), public health agencies have responded aggressively to the urgent need to acquire knowledge regarding this emerging infection. An important component of this urgently needed knowledge includes understanding the effectiveness of alternative treatment strategies in patients with suspected or proven infection. It should also be noted that clinical guidance issued by the WHO indicates that unproven therapies should be administered preferably only as part of a clinical trial (https://www.who.int/docs/default-source/coronaviruse/clinical-management-of-novel-cov.pdf).

Estimates of the burden of critical illness among patients infected with COVID-19 vary, with estimates of case-fatality and proportion of patients who become critically ill being unstable. Several factors contribute to this uncertainty including differential timing between diagnosis and development of critical illness or death, the true incidence of infection being uncertain because of possible under-reporting of asymptomatic or mild cases, the sensitivity of diagnostic methods, possible limitation on the number of diagnostic tests that can be performed, and changing case-definitions. Nevertheless, it is recognized that fatal pneumonia is common and that there is potential for widespread disease activity outside China.

There have been several reports of clinical disease from Chinese investigators. These reports describe a progressive severe pneumonia, with a significant proportion requiring mechanical ventilation and some reports of multi-organ dysfunction. In a report of three cases who developed clinical and radiographic features of pneumonia, one patient required mechanical ventilation and died subsequently (Zhu et al., 2020) In a study of 41 hospitalized patients with laboratory-confirmed COVID-19 infection, 13 (32%) patients were admitted to an ICU and six (15%) died. Invasive mechanical ventilation was required in four (10%) patients, with two patients (5%) receiving extracorporeal membrane oxygenation as salvage therapy (Huang et al.). In another study of 99 hospitalized patients with COVID-19 pneumonia, 23 (23%) were admitted to ICU, 17 (17%) developed acute respiratory distress syndrome (ARDS), three (3%) acute renal failure and four (4%) septic shock. [3] In a study of 138 patients with COVID-19 infection, 36/138 required ICU care. Patients admitted to ICU were older and were more likely to have underlying comorbidities. In the ICU, four patients (11.1% of those admitted to ICU) received high-flow oxygen and 15 (44.4%) received

SUPERSEDED

noninvasive ventilation. Invasive mechanical ventilation was required in 17 patients (47.2%), four of whom received extracorporeal membrane oxygenation as rescue therapy. A total of 13 patients received vasopressors and two patients received kidney replacement therapy (Wang et al., 2020b). In a study from the Chinese Centers for Disease Control that reported on 72,314 patients, 49% of patients defined as critically ill died before hospital discharge (1023 of 2087) (Wu and McGoogan, 2020).

As with the other major coronaviruses that have circulated in outbreaks in recent decades, SARS and MERS-CoV, no specific immune modulation therapy, or an element of supportive care, has been formally evaluated in randomized controlled trials. Currently, randomized trials are ongoing for infected patients with MERS-CoV in Saudi Arabia, examining the role of lopinavir/ritonavir + IFN-β1a, compared to standard care alone (Arabi et al., 2018). These agents were chosen due to biologic plausibility, given *in vitro* evidence suggesting effect against MERS-CoV. Other proposed strategies for acute management of these patients include immunomodulatory therapies, the use of non-approved antiviral agents, and specific antibody formulations.

Interim recommendations from the WHO for clinical care of infected patients focus upon supportive care, including organ support as needed, prevention of complications, and no specific antivirals at this point in time, with any specific therapy to only be provided as part of a research protocol (https://www.who.int/docs/default-source/coronaviruse/clinical-management-of-novel-cov.pdf).

5.2.2. Clinical trials for COVID-19 infection

5.2.2.1. Current clinical trials and interventions being evaluated

As of 24th February 2020, more than 150 clinical studies from China had been registered on trial registration sites. Many of these trials are single center and with sample sizes that are unlikely to be sufficient to detect plausible treatment effects, with some studies being uncontrolled or observational. There is also a rapid decline in incidence of new infection in China and many clinical trials are unlikely to achieve their planned sample size.

A wide range of interventions are being evaluated in trials that have been registered including arbidol, lopinavir/ritonavir, darunavir/cobicistat, remdesivir, favipiravir, baloxavir, chloroquine, intravenous immunoglobulin, inhaled and parenteral IFN-α or IFN-β glucocorticoids (different agents and doses), mesenchymal and other stem cells, microbiota transplantation, and a range of traditional Chinese medicines.

SUPERSEDED

WHO has provided guidance regarding both trial design and prioritization of candidate therapies. With regards to trial design, WHO notes that there are no antivirals with proven efficacy in patients with COVID-19. As such, WHO guidance is that trials should utilize a 'standard of care' comparator, that is, a control group that does not receive an agent intended to be active against COVID-19 infection or its associated immune response (https://apps.who.int/iris/bitstream/handle/10665/330694/WHO-HEO-RDBlueprintnCoV-2020.4-eng.pdf?ua=1). WHO recommend that trials be conducted with lopinavir/ritonavir, an antiviral licensed for use in patients with Human Immunodeficiency Virus infection and that this agent is evaluated in clinical trials either alone or in combination with IFN-β1 https://apps.who.int/iris/bitstream/handle/10665/330694/WHO-HEO-RDBlueprintnCoV-2020.4-eng.pdf?ua=1).

At launch, this domain will evaluate two active interventions that are designed to modify host immune responses to viral infection, particularly around the severity of viral pneumonitis and progression to ARDS. These agents are IFN-β1a and anakinra, which acts as an interleukin-1 receptor antagonist. The inclusion of IFN-β1a will allow evaluation of combination therapy with lopinavir/ritonavir with the use of this antiviral agent being specified in a separate DSA. Additional agents may be added subsequently.

5.2.2.2. Need for evidence in patients who are critically ill

There is need to evaluate interventions for COVID-19 infection in patients who are critically ill. The number of current studies that are focused on patients who are critically ill is uncertain and, for those studies that are enrolling hospitalized patients, it is unclear if stratification by severity is a design feature. The need for studies that focus on patients who are critically ill arises because of the possibility of differential treatment effect between patients who are critically ill compared with noncritically ill patients.

There are two reasons for this possibility, one generic to all interventions evaluated in the critically ill and one that is specific to immune modulation therapy. Firstly, among trials that evaluate interventions in patients who are critically ill it is common for the results of the trial to be different to that which was predicted based on a prior understanding of mechanism of action combined with known mechanism of disease (Landoni et al., 2015, Webb, 2015). This observation reinforces the importance of not necessarily relying on extrapolation of results (both positive and negative) from patients who are not critically ill. Secondly, it is likely that the immune response that drives transition from viral pneumonitis to ARDS occur only or predominantly in patients who are critically

SUPERSEDED

ill or becoming critically ill (Peiris et al., 2003). As such, exposure to immune modulation therapy for patients with mild or moderate disease may have different treatment effects compared with patients who are critically ill.

5.2.2.3. Need for evidence that takes into account concomitant therapy

As far as can be ascertained, all current clinical trials evaluate a single strategy, such as antiviral therapy or immune modulation. However, it is biologically plausible that there is interaction between antiviral and immune modulatory therapies or interaction among different immune modulatory therapies or both. For example, an immune modulation strategy that dampens the host immune or inflammatory response may also result in uncontrolled viral replication. As such, administration of immune modulation strategy may be harmful in the absence of co-administration of antiviral agent, an immune modulation strategy may be effective only in the presence of co-administration of an active antiviral agent, and an antiviral agent may be ineffective alone but effective when co-administered with an agent that modulates the immune response. Additionally, it is plausible that combinations of different immune modulation therapy may be synergistic or antagonistic. As noted below, this is particularly relevant for the combination of corticosteroids and IFN-β1a. The importance of testing combinations of agents is reinforced by the observation of the widespread use of these agents, including in combination, in published case series (Chen et al., 2020, Guan et al., 2020, Huang et al., 2020, Wang et al., 2020a).

In this regard, and within REMAP-CAP, the COVID-19 Immune Modulation Therapy Domain should be considered in conjunction with the COVID-19 Antiviral Therapy Domain and the pre-existing Corticosteroid Domain of REMAP-CAP. The pandemic statistical model, as described from the PAtC, will allow evaluation of interactions between these domains, as specified in DSAs that are specific for COVID-19 infection.

5.2.3. Intervention strategy for this domain

It is possible that evidence will emerge from a range of clinical trials regarding appropriate immune modulation strategy for patients who are critically ill. It is intended that this domain of REMAP-CAP will evolve, taking into account evidence derived from other clinical trials, as well as availability of potentially effective immune modulation therapies. WHO guidance notes the flexibility associated with REMAP-CAP as a platform for the testing of multiple agents, including serial testing of additional interventions (https://apps.who.int/iris/bitstream/handle/10665/330680/WHO-HEO-RDBlueprint%28nCoV%29-2020.1-eng.pdf?ua=1)

SUPERSEDED

At the commencement of this domain, a control group is included, i.e. some patients will not receive an immune modulation agent, either in this domain or in the Corticosteroid Domain that is intended to be active during COVID-19 infection. This is appropriate for two reasons. Firstly, there is relatively limited trial or clinical experience with the administration of these immune modulation agents in patients who are critically ill due to viral infection, and it is not reasonable to presume that such agents are not capable of causing net harm. Secondly, designs that included only active interventions are not able to ascertain if any option is better or worse than no treatment. If, during the evolution of this domain, there is sufficient evidence of effectiveness of agents or clinical practice changes to include the routine use of such agents or both, the control intervention that specifies that no immune modulatory agent is administered will be abandoned.

Although this domain will commence with two active immune modulation therapies, it is intended that additional agents can be added (allowing evaluation of several agents against a common control intervention) as well as allowing introduction of combinations of agents (to evaluate potential synergy). Any changes to the intervention structure of the domain will be specified using one or more amendments to this DSA with implementation occurring only after ethical approval has been obtained. The initial selection of agents to be evaluated are IFN-β1a and anakinra. Commencing with Version 2.0, additional agents have been added and are outlined below.

If at any stage evidence of harm or definitive evidence of absence of effectiveness in critically ill patients emerges for any intervention specified in this domain, the ITSC, as advised by the DSWG, may remove an intervention prior to declaration of a Platform Conclusion. If this occurs, presentation and publication of results that relate to that intervention will occur, so as to contribute additional weight of evidence in the public domain.

5.2.4. IFN-β1a

5.2.4.1. Biological rationale and pre-clinical studies

Through mechanistic studies, humans infected with coronaviruses have been shown to have blockade of the interferon response during critical illness. SARS-CoV and MERS-CoV attenuates the interferon response of the innate immune system, and this mechanism is thought to impair the antiviral adaptive type 1 T helper cell (Th-1) immune response (Lau et al., 2013, Faure et al., 2014, Zielecki et al., 2013, Frieman et al., 2008). Because of this, supplementing interferon has been long thought biologically plausible for acutely infected patients, and has been administered in a number of settings, including SARS and MERS-CoV infected patients across the world.

SUPERSEDED

In vitro data demonstrate that IFN-α and IFN-β have inhibitory effects on MERS-CoV and SARS-CoV (de Wilde et al., 2013, Zielecki et al., 2013). Different preparations of recombinant rIFNs (rIFN-α2a, rIFN-α2b, rIFN-β1a and rIFN-β1b) are active against MERS-CoV in-vitro (Hart et al., 2014) although IFN-β1b causes the greatest *in vitro* inhibition of MERS-CoV (Chan et al., 2013, Hart et al., 2014). One study examined the *in vitro* MERS-CoV susceptibility to different rIFN preparations (rIFN-α2b, rIFN-γ, rIFN-universal, and rIFN-α2a, rIFN-β) and found that rIFN-β had the strongest MERS-CoV inhibition, at 41 times lower than the previously reported 50% inhibitory concentration (IC50) (56.08 U ml-1) of rIFN-α2b (Hart et al., 2014). Another *in vitro* study found that serum concentrations achievable at therapeutic doses of rIFN-β-1b were 3-4 times higher than the *in vitro* inhibitory concentrations of MERS-CoV, whereas those of other rIFN preparations and RBV were lower than inhibitory levels (Chan et al., 2013). Recombinant IFN-β1b (Betaseron®, Bayer, Leverkusen, Germany) is approved for multiple sclerosis (Jankovic, 2010, Hurwitz et al., 2008, Gottesman and Friedman-Urevich, 2006).

The effects of lopinavir/ritonavir, IFN-β1b and mycophenolate mofetil (MMF), all of which have shown viral inhibitory effects *in vitro*, have been tested in common marmosets with severe MERS-CoV infections (Chan et al., 2015). The animals treated with lopinavir/ritonavir or IFN-β1b had improved clinical, radiological, pathological and viral-load outcomes compared with untreated animals. By contrast, treatment with MMF resulted in severe or fatal disease, with higher mean viral loads than in untreated animals. Untreated animals and MMF-treated animals had a mortality of 67% by 36 hours compared to 0–33% among animals treated with lopinavir/ritonavir or IFN-β1b (Chan et al., 2015).

In addition, IFN-β may help attenuate lung injury (i.e. ARDS). In addition to a direct key role against viral infections, IFN-β has an identified role in enhancing endothelial barrier function through upregulation of the production of adenosine. INF beta-1a activates Cluster of Differentiation 73 (CD73), a cell surface enzyme that converts adenosine from adenosine monophosphate. Adenosine has ability to enhance endothelial barrier function under vascular leakage. Preclinical studies have shown that CD73 expression on endothelial cells is up-regulated by IFN-β1a treatment in a time- and dose-dependent fashion (Kiss et al., 2007). In a “two-hit” model, mice were subjected to cecal ligation and puncture, followed four days later by delivery of intratracheal *Pseudomonas aeruginosa*. Administration of subcutaneous IFN-β one day before bacterial challenge reduced the odds ratio for 7-day mortality by 85% (odds ratio, 0.15; 95% confidence interval, 0.03–0.82; $P = 0.045$) (Mould and Janssen, 2018, Hiruma et al., 2018).

SUPERSEDED

5.2.4.2. Clinical studies of IFN-β1a

5.2.4.2.1. Coronavirus infection

During the Korean outbreak of MERS, most patients that developed respiratory illness received triple antiviral therapy composed of pegylated IFN-α, ribavirin, and lopinavir/ritonavir; however, data about the efficacy of this approach is lacking (Min et al., 2016). These findings, together with the availability and safety profiles of lopinavir/ritonavir and IFN-β1b, suggest that the combination of these agents has potential efficacy for the treatment of patients with MERS. At present, the MIRACLE trial (the MERS-CoV Infection tReated With A Combination of Lopinavir/Ritonavir and IntErferon-β1b) is being conducted in Saudi Arabia to assess the efficacy of administering a combination of lopinavir/ritonavir and recombinant IFN-β1b to hospitalized adults with laboratory-confirmed MERS (Arabi et al., 2018).

5.2.4.2.2. ARDS

In an open-label, non-randomized, phase 1–2 study of intravenous human recombinant IFN-β1a (FP-1201) in patients with ARDS, an optimal tolerated dose (OTD) in the first, dose-escalation phase and subsequently enrolled patients were given the OTD of intravenous FP-1201 for 6 days (Bellingan et al., 2014). By day 28, 3 (8%) of 37 patients in the treatment cohort and 19 (32%) of 59 patients in the control cohort had died (odds ratio 0·19 [95% CI 0·03–0·72]; p=0·01).

Following this phase 1-2 study, the phase III randomized placebo-controlled multicenter international INTEREST trial (FPCLI002, clinicaltrials.gov: NCT02622724) has been completed and published recently (Ranieri et al., 2020). In this trial, comprising 300 critically ill patients with confirmed ARDS, the use of 10 µg daily dose of lyophilized IFN-β1a (FP-1201-lyo) for six days was not associated with reduced mortality or ventilator use. The day 28 mortality in the FP-1201-lyo group was 26% and in the placebo group 23%. Two-thirds of the ARDS patients had CAP as the underlying cause for ARDS. The proportion of patients with a viral cause of CAP was not recorded (unpublished data, Ville Petilla). However, based on the usual, i.e. non-pandemic distribution of different causes of CAP, it is reasonable to presume that a minority of these patients would have had a viral cause of CAP.

Post-hoc analysis of the INTEREST trial suggested a strong negative interaction between corticosteroid administration and IFN-β1a with corticosteroids inhibiting the biological effect of IFN-β1a. Subjects receiving FP-1201-lyo without concomitant glucocorticoids had 28-day mortality of 11% compared with 32% mortality in subjects receiving concomitant glucocorticoids. Additional *in vitro* and *ex vivo* analyses of human lung tissue demonstrated that glucocorticoids inhibit the effect

SUPERSEDED

of IFN-β1a to up-regulate CD73 on lung capillary endothelium, which is proposed to be the mechanism of any beneficial effect of IFN-β1a (unpublished data under review).

In the IFN-β1a treatment arm of the INTEREST trial 54% of patient received corticosteroids during the 28-day study period, 56 % of these were receiving corticosteroids at randomization, 27% received corticosteroids during IFN-β1a treatment (day 1 – 6), and 17% received corticosteroids after IFN-β1a treatment (day 7 onwards). Respective day 28 mortality rates were 50%, 33%, and 15% respectively. In the post-hoc propensity-matched analysis of the IFN-β1a arm, baseline systemic corticosteroid treatment was independently associated with day 28 mortality (OR 5.4, 95% CI 2.1 – 13.9, P < 0.001) and corticosteroid treatment overlapping with IFN-β1a treatment (D1 – D6) also independently associated with D28 mortality (OR 3.9, 95% CI 1.9 – 12.7, P = 0.008) (unpublished data under review).

These results support the possibility of benefit from IFN-β1a treatment when administered alone and a harmful interaction between when there is co-administration of corticosteroids and IFN-β1a. This is potentially relevant to REMAP-CAP because of the presence of a separate domain that includes administration of corticosteroids. However, the validity of this association and its relevance to patients with COVID-19 infection is uncertain. Firstly, the patient population enrolled in the INTEREST study enrolled patients with ARDS of any cause. It is likely that few if any patients will have had ARDS secondary to viral pneumonia. Secondly, the evaluation of the possible interaction between corticosteroids and IFN-β1a is a post hoc observational analysis in which residual confounding, caused by severity of illness, may be responsible for some or all of the observed association. Thirdly, it is likely that there will be co-administration of these agents in routine patient care and occurrence of this has been reported by Chinese physicians on WHO COVID-19 Clinical Network conference calls as well as use of corticosteroids in reported case series (Chen et al., 2020, Guan et al., 2020, Huang et al., 2020, Wang et al., 2020a, Wu and McGoogan, 2020). As such, it is important to determine the effectiveness of corticosteroids alone, IFN-β1a alone, and evaluate, as rapidly as possible, any harmful interaction.

5.2.4.3. Safety profile

Recombinant human IFN-β1a is an approved for intramuscular or subcutaneous administration in patients with relapsing and remitting multiple sclerosis. The safety profile in ambulatory patients is well characterized with adverse events being rare and including pulmonary hypertension, new onset autoimmune disease, neutropenia, and thrombocytopenia, thrombotic microangiopathy, and

SUPERSEDED

worsening of cardiac angina (REBIF® - IFN-β1a Medical Product Summary available on the website of the European Medicines Agency, http://www.ema.europa.eu)

The use of IFN-β1a (FP-1201-lyo) in critically ill patients with ARDS is not associated with substantially increased risks or adverse events in comparison to placebo. Fever was the most common adverse event in both phase III trials (Ranieri et al., 2020, Bellingan et al., 2014). In the INTEREST trial (Ranieri et al., 2020), 18 (12.5%) patients and 12 (7.9%) patients in the FP-1201-lyo group and placebo groups, experienced fever as an Adverse Event (AE). Anemia was the second most common AE occurring in (16 [11.1%] patients and 11 [7.2%] patients in the FP-1201-lyo group and placebo groups, respectively. In the Japanese phase III trial (MR11A8-2) fever was reported more frequently (34.7% and 6.8%) as an AE for FP-1201-lyo and placebo groups, respectively. The other commonly reported AEs were decreased platelet count (6.1%) in the FP-1201-lyo group and increased hepatic enzymes (6.8% in both groups).

The incidence of serious adverse events (SAEs) in the INTEREST trial (fatal and non-fatal) was similar in the active (53.5%) and placebo (50.7%) groups. The overall incidence of AEs considered related to study drug was higher in the active group (28.5%) compared with the placebo group (21.7%), with the difference explained mostly by difference in fever. The incidence of SAEs in the MR11A8-2 trial (fatal and non-fatal) was similar in the active (53.1%) and placebo (47.7%) groups, but none of the events were strongly suspected of being causally related to the study drug. These trials, conducted in critically ill patients provides evidence that the safety profile in this patient population is acceptable.

5.2.4.4. IFN-β1a dosing

In previous trials (Bellingan et al., 2014, Ranieri et al., 2020) enrolling patients with ARDS, IFN-β1a 10 µg has been administered by dilution 1 mL of sterile water which is then administered as an intravenous bolus injection via a central or peripheral line. The dose selected for this study is based on information from the previous studies, where the maximum tolerated dose was found to be 22 µg. In some regions IFN-β1a is available in a preparation containing 22 µg in a 0.5mL syringe. Therefore the dose in this trial will be 10-11 µg, depending on the preparation available. A dose of 10 µg was shown to be the OTD based on information from dose-limiting toxicity and proven markers of IFN-β1a biological activity. In these trials IFN-β1a was administered once daily for 6 days or until ICU discharge, whichever occurred first. In the immune modulation domain of REMAP-CAP IFN-β1a will be administered using the administration regimen used in the INTEREST trial (Ranieri et al., 2020).

SUPERSEDED

5.2.5. Anakinra

5.2.5.1. Biological rationale and pre-clinical studies

The clinical pathway to critical illness and death in patients with viral pneumonitis, such as that caused by influenza or SARS, involves development of ARDS. It is likely that a similar pathway occurs in COVID-19. In patients with ARDS secondary to influenza and SARS there is evidence of cytophagocytosis on histopathological examination (Harms et al., 2010). Cytophagocytosis is a hallmark of macrophage activation syndrome (MAS), which is an inflammasome/Interleukin-1(IL-1)-mediated disease (Grom et al., 2016). MAS refers to an acute overwhelming inflammation caused by a cytokine storm and is recognized as a life-threatening complication of various diseases, including infection (Grom et al., 2016, Shakoory et al., 2016). The clinical and laboratory features of MAS include sustained fever, hyperferritinemia and high IL-18, pancytopenia, fibrinolytic consumptive coagulopathy, and liver dysfunction (Schulert and Grom, 2015, Rigante et al., 2015, Grom et al., 2016).

Genetic susceptibility studies also link MAS to outcome in viral pneumonitis. A whole exome study performed in fatal cases of H1N1 with signs of MAS found a high percentage of mutations in genes that are linked to genetic causes of diseases similar to MAS, suggesting that a genetic background might predispose to developing MAS in influenza (Schulert et al., 2016). Underscoring the importance of the inflammasome in MAS is the observation that a monogenetic mutation in the inflammasome underlies primary MAS (Canna et al., 2014).

There is also evidence of excessive inflammatory activation and MAS in viral pneumonitis and ARDS caused by infection with Coronavirus. In the case series of critically ill patients with SARS from Toronto and Singapore respiratory infection lead to death by first causing acute lung injury and multiple organ failure (Booth et al., 2003, Lew et al., 2003). Interestingly, in contrast to immunoparalysis seen in sepsis, this acute lung injury was thought to be due to an exacerbated innate host response to SARS-CoV (Gralinski et al., 2018, Smits et al., 2010). Another study revealed high IL-18 levels (Baas et al., 2006), a cytokine that is associated with MAS (Schulert and Grom, 2015) and MAS has also been reported in SARS (Nicholls et al., 2003). Similar pulmonary hyperinflammation was seen on histology in MERS patients (Arabi et al., 2017). In COVID-19 elevated IL-6 and ferritin levels have been reported (Chen et al., 2020). The mean ferritin level was 808.7 ng/ml in 99 patients with COVID-19 pneumonia of whom 23% were admitted to the ICU. The main reason patients were admitted to the ICU was because of ARDS.

SUPERSEDED

There is pre-clinical evidence that inflammasome-mediated disease is amendable to treatment that targets IL-1 signaling, such as recombinant IL-1 receptor antagonist (rIL-1Ra; anakinra) (Cavalli and Dinarello, 2018). Therefore, anakinra treatment may be an effective targeted therapy in viral pneumonitis due to SARS-CoV2. Anakinra neutralizes the biologic activity of IL-1a and IL-1b by competitively inhibiting their binding to interleukin-1 type I receptor (IL-1RI) and preventing signaling of the IL-1RI (Arend et al., 1990). Interleukin-1 (IL-1) is a pivotal pro-inflammatory cytokine mediating many cellular responses.

Anakinra is widely used in rheumatic diseases, but has also been evaluated previously for treatment of critically ill patients with sepsis and septic shock (Fisher et al., 1994, Knaus et al., 1996, Opal et al., 1997). In these trials, for patients with sepsis, with a combined sample size of almost 2000 patients, anakinra did not reduce overall all-cause mortality. However, a post-hoc analysis indicated that survival was improved in the subgroup of sepsis patients with features of MAS (ferritin elevation in excess of 2,000 ng/ml, coagulopathy, and liver enzyme elevations) (Shakoory et al., 2016). Such a post hoc finding should only be regarded as hypothesis generating, but the finding does support a potential beneficial effect of anakinra, particularly in the treatment or prevention of development of ARDS in patients with viral pneumonitis due to its association with MAS.

5.2.5.2. Safety profile

There is extensive experience with the use of anakinra in ambulatory patients with rheumatic and inflammatory diseases. In placebo-controlled trials that enroll patients with rheumatoid arthritis the adverse reactions reported most frequently were injection site reactions, which were mild to moderate in the majority of patients. This is not an issue when anakinra is administered intravenously as will occur in this trial. The incidence of SAEs with anakinra (100 mg/day) was comparable with placebo (7.1% compared with 6.5% in the placebo group) (https://www.ema.europa.eu/en/medicines/human/EPAR/kineret). The incidence of serious infection was higher in anakinra-treated patients compared to patients receiving placebo (1.8% vs. 0.7%) (https://www.ema.europa.eu/en/medicines/human/EPAR/kineret). Neutropenia occurred more frequently in patients receiving anakinra compared with placebo, 2.4% compared to 0.4%, respectively (https://www.ema.europa.eu/en/medicines/human/EPAR/kineret). In clinical trials that recruited patients with septic shock or severe sepsis, anakinra was well tolerated with no difference in occurrence of AEs and SAEs between anakinra and placebo (Fisher et al., 1994, Knaus et al., 1996, Opal et al., 1997). In these studies anakinra was administered at doses of up to 2 mg/kg/hour intravenously (which is 35 times the standard dose in rheumatoid arthritis over a 72-hour treatment period).

SUPERSEDED

5.2.5.3. Anakinra dosing

To neutralize IL-1 signaling completely, IL-1Ra must be present in at least a concentration 1000 times higher than IL-1 (Arend et al., 1990). When this occurs, signaling is blocked for at least 4-6 hours. The trial will use a dose of 300 mg intravenously once initially to reach the amount of IL-1Ra that can completely block IL-1 signaling. It will then continue at a maintenance dose of 100 mg intravenously every 6 hours to maintain adequate drug levels to block IL-1. This is a much lower dose than 2 mg/kg per hour administered in previous clinical and pre-clinical studies that recruited patients with severe sepsis and septic shock, which did not report serious side effects.

The mean plasma clearance of anakinra in subjects with mild (creatinine clearance 50-80 ml / min) and moderate (creatinine clearance 30-49 ml/min) renal failure was reduced by 16% and 50%, respectively, and it is stated that anakinra can be given once daily in a similar dose as for normal renal function. However, with severe renal failure, end stage renal disease (creatinine clearance < 30 ml/min), and renal replacement therapy, maintenance anakinra doses should be given at 12 hourly intervals (https://www.ema.europa.eu/en/medicines/human/EPAR/kineret).

5.2.6. IL-6 receptor antagonists

5.2.6.1. Biological rationale

Experimental models of Coronavirus infection support a role for IL-6 in pathogenesis. In SARS, high levels of interleukin (IL)-6 are released from monocytes and macrophages in response to interaction between the viral spike (S) protein and cell-surface TLR-2 (Jacques et al., 2009). In this model, the release of IL-6 and TNF-α was mostly dependent on the activation of the ERK-1/2 MAPK and JNK pathways and, to a lesser extent, the p38 MAPK and NF-kB pathways. The pathology induced by in a mouse Coronavirus model, utilizing the Mouse Hepatitis Virus, was mediated by the release of intrahepatic IL-6 and TNF-a via the TLR2 receptor, as demonstrated in TLR2 knockout mice and by histopathological observations. In a mouse model of SARS, excessive secretion of IL-6 and IL-8 exacerbate pulmonary pathogenesis by modulating intrinsic functions of monocyte-derived macrophages and dendritic cells. These cytokines may also facilitate a mechanism for evading the host immune response by delaying and inhibiting the ability of dendritic cells to prime naïve T cells. This provides a dual rationale for the potential benefit of IL-6 blockade in Coronavirus infection.

In patients with COVID-19, increased secretion of IL-6 was observed in monocytes and CD4+ cells of patients who developed a severe clinical course, in comparison with patients who had uncomplicated infection or healthy controls (Zhou et al., 2020b) (https://www.medrxiv.org/content/10.1101/2020.02.25.20025643v1.full.pdf). These preclinical

SUPERSEDED

findings together suggest that IL-6 may be a key mediator in severe COVID-19 and is a suitable target for immunomodulatory therapy.

5.2.6.2. Tocilizumab

5.2.6.2.1. Clinical studies of tocilizumab

Tocilizumab is a humanized monoclonal antibody that inhibits both membrane-bound and soluble IL-6 receptors. IL-6, which is secreted by monocytes and macrophages, is one of the main drivers of immunologic response and symptoms in patients with cytokine-release syndrome (CRS). While tocilizumab was first approved by the Food and Drug Administration (FDA) in 2010 for the treatment of rheumatoid arthritis, it has received additional approval for treatment of patients with giant cell arteritis, and systemic and juvenile forms of idiopathic arthritis. In 2017, Tocilizumab received additional approval for the treatment of severe or life-threatening CAR T-associated CRS due to its efficacy and safety profile. While criteria for grading CRS severity varies by cancer center, it has been proposed to administer tocilizumab to CRS patients with any of the following: oxygen requirement < 40%, hypotension responsive to fluids or a low dose of a single vasoactive agent, or Grade 2 organ toxicity as defined by the Common Terminology Criteria for Adverse Events.(Lee et al., 2018)

Hyperinflammatory states and cytokine storming, including elevated IL-6, has been reported in patients with severe COVID-19 and were associated with increased mortality in patients in China (Zhou et al., 2020a). A pre-print (non-peer reviewed) case series of 21 patients treated with tocilizumab between February 5-14, 2020 in China reported clinical success, with rapid resolution of fever and C-reactive protein, decreased oxygen requirements, and resolution of lung opacities on computerized tomography imaging. The authors state the patients all had “routine treatment for a week” before tocilizumab, which was described as “standard care according to national treatment guidelines” including lopinavir, methylprednisolone, and other supportive care. All patients had IL-6 analyzed prior to tocilizumab administration with a mean value of 132.38 ± 278.54 pg/mL (normal < 7 pg/mL). No adverse events were described in this case-series although no assessment of long-term outcomes was reported. If effective, there is uncertainty regarding optimal timing of administration or of possible differential treatment effect depending on stage of disease.

There are several clinical studies of tocilizumab in COVID019 underway. NCT04317092 is a single arm study of 330 participants who are hospitalised due to COVID-19 and have either low oxygen saturations (<93% on air) or have been intubated for <24 hours. All patients will be treated with tocilizumab (8mg/kg) and the primary outcome is one-month mortality. NCT04320615 will evaluate the efficacy, safety, pharmacodynamics, and pharmacokinetics of tocilizumab (8mg/kg) compared

SUPERSEDED

with a matching placebo in combination with standard of care (SOC) in hospitalized patients with severe COVID-19 pneumonia. Participants will be hospitalised patients due to COVID-19 with oxygen saturations ≤93% or a PaO2 / FiO2 ratio of 300mmHg. The primary outcome is Clinical Status Assessed Using a 7-Category Ordinal Scale at day 28. There are also anecdotal reports of the off-trial use of tocilizumab in several countries including Italy, United Kingdom, and Australia. As outlined earlier WHO guidance and Chief Medical Officer guidance in the United Kingdom (https://www.nihr.ac.uk/news/uks-top-doctors-urge-recruitment-of-covid-19-patients-for-vital-scientific-research/24591), there is an imperative that interventions that are being used by clinicians, without evidence of the balance between risks and harms, are incorporated within controlled clinical trials as rapidly as possible.

5.2.6.2.2. Safety profile

There is extensive experience with the use of tocilizumab for ambulatory patients with rheumatoid arthritis and other inflammatory diseases as part of pivotal clinical trials or post-marketing surveillance. For patients who participated pivotal phase III trials for rheumatoid arthritis and were treated with 4 to 8mg/kg of intravenous tocilizumab, the most common adverse effects (incidence range 3-8%) over a follow-period of 6 months were upper respiratory tract infections, nasopharyngitis, headache, hypertension, and transaminitis (https://www.actemrahcp.com/ra/clinical-study-safety/clinical-study-safety.html). The incidence of these adverse events was slightly higher compared to patients treated with methotrexate or other disease modifying agents. The most common serious adverse events were infections (cellulitis, herpes zoster, gastroenteritis, diverticulitis, sepsis, and bacterial arthritis). The cumulative incidence of serious infections was 5 per 100 patient years (PY) compared to 4 per 100 PY for other disease modifying agents. Less commonly observed serious adverse events were new medically confirmed malignancies (1.3 per 100 PY), myocardial infarctions (0.3 per 100 PY), hepatic events (0.04 per 100 PY), and medically confirmed gastrointestinal perforations (0.2 per 100 PY). IL-6 antagonism has been associated with neutropenia and thrombocytopenia in patients receiving chronic therapy with tocilizumab for giant cell arteritis or rheumatoid arthritis (https://www.medicines.org.uk/emc/product/6673/smpc#PRODUCTINFO).

In a case series of 53 adult patients with relapsed or refractory B-cell acute lymphoblastic leukemia, Grade 3 CRS or higher was associated with increased risk of subsequent infection but it was unclear whether tocilizumab or corticosteroid use promoted this risk (Park et al., 2018). There were no reported adverse events in the 60 tocilizumab-treated patients submitted to the FDA for the CRS indication, which recommends a maximum of 4 doses for treatment (Lee et al., 2019).

SUPERSEDED

To date, to the best of our knowledge, only an uncontrolled case series of 21 patients (17 assessed as severe and 4 as critical) treated with tocilizumab has been published as a pre-print (chinaXiv:202003.00026v1). In this case series, 18 patients received one dose (400mg) and 3 patients had a repeat dose within 12 hours. All patients had a return to normal body temperature. Fifteen patients had reduced oxygen requirements, and two were extubated within 5 days. CT scans were reported as showing improvement in 19 patients. At the time of the report 90% of the patients had been discharged from the hospital. There were no reports of secondary pulmonary infections or other deteriorations. No adverse drug reactions were reported during the treatment. It should also be noted that pharma sponsored trials of IL-6 blockade have been registered.

Although it is clear that Tocilizumab can increase the risk of infection, and it is acknowledged that patients who are critically ill are at risk of secondary bacterial infections, these risks should be interpreted in the context of short-term treatment in patients with COVID-19 who have high reported mortality. As such, it is reasonable to conclude that the potential benefits of treatment with tocilizumab as anti-inflammatory agent outweigh the known potential risks.

5.2.6.2.3. Tocilizumab dosing

Immunotherapy with tocilizumab is listed as a treatment option for severe or critical cases of COVID-19 with elevated IL-6 in the 7th edition of the National Health Commission of the People's Republic of China COVID-19 Diagnosis and Treatment Guide (http://kjfy.meetingchina.org/msite/news/show/cn/3337.html). The recommended dose is 4-8 mg/kg with the option to repeat a dose in 12 hours.

5.2.6.3. Sarilumab

5.2.6.3.1. Pre-clinical studies of sarilumab

Sarilumab is a recombinant humanized monoclonal antibody (IgG1) that binds to both soluble and membrane-bound human interleukin-6 receptor alpha (IL-6Rα) to inhibit IL-6 mediated signaling. Three phase I randomized, double-blind, placebo-controlled trials of sarilumab in rheumatoid arthritis were carried out in a total of 83 active treatment and 24 placebo patients using ascending doses of 50, 100, 150, and 200 mg (June and Olsen, 2016). A dose-dependent reduction in levels of acute phase reactants was observed, with a greater than 90% reduction in high sensitivity c-reactive protein (hs-CRP) and serum amyloid A after administration of the 200 mg dose of sarilumab. Compared to tocilizumab, the *in vitro* binding affinity of sarilumab (K 61.9 pM) for the human interleukin-6 receptor (IL-6R) is 15–22-fold higher (June and Olsen, 2016).

SUPERSEDED

5.2.6.3.2. Clinical studies of sarilumab

Safety and efficacy of sarilumab was evaluated in a combined phase II /III multicenter, randomized, double-blind placebo-controlled studies in 1588 patients with rheumatoid arthritis (Huizinga et al., 2014, Genovese et al., 2015). Patients were randomized to placebo or five different subcutaneous dosing regimens (100/150/200 mg every 2 weeks; 100/150mg every week). Significant clinical responses compared to placebo were seen after 12 weeks of treatment across all doses of 150 mg Q2 weeks or greater. Disease activity at day 28 combined with c-reactive protein levels responses showed a dose response relationship with sarilumab and the highest remission response was seen in the 150 mg weekly regimen.

An adaptive phase 2/3, randomized, double-blind, placebo-controlled study assessing efficacy and safety of sarilumab for hospitalized patients with COVID-19 (NCT04315298) is currently enrolling with a planned sample size of 400 patients. The trial evaluates a single dose of 400mg intravenous sarilumab, in comparison with placebo. At this time, the rate of recruitment into this trial is not known. Simulations conducted to support the development of the pandemic component of REMAP-CAP indicate that 200 patients enrolled to active treatment would have sufficient power to detect only a very strong treatment effect. Additional trials comparing the efficacy and safety of alternative dosing regimens are in planning (NCT 04324073, NCT 04322773, NCT 04321993).

5.2.6.3.3. Safety profile of sarilumab

The safety profile of sarilumab in ambulatory patients is well established. No dose-limiting toxicities were observed in the phase II/III trials of sarilumab. In phase II/part A trial of 306 patients with active rheumatoid arthritis, who also received concurrent treatment with methotrexate, dose-related neutropenia was observed. The level of Alanine Aminotransferase (ALT) increased to more than three times the upper limit of normal in 4% of patients and there was elevation of serum cholesterol levels in 10 to 20% of patients after 12 weeks of treatment (June, 2016). In the phase III/part B of the trial, which included 1282 patients in the safety analysis, AEs and SAEs were more common in the sarilumab group (11.3% in the 200-mg group vs. 5.4% placebo). The most common AEs and SAEs were related to infections. Injection site reactions were reported in approximately 10% of patients treated with sarilumab. Eight malignancies occurred, with a higher incidence reported among patients treated with sarilumab. Neutropenia in the range of 0.5–1.0 × 109 /L was observed in 5.1% of the sarilumab 150-mg and 7.8% of the 200-mg groups and occurred in a dose-dependent manner. Increase in liver function parameters occurred in 9.5% of the 150-mg sarilumab group subjects versus 2.1% of the placebo group subjects. Increase in serum cholesterol occurred in 43% of the sarilumab group subjects versus 18% of placebo group subjects.

SUPERSEDED

5.2.6.3.4. *Sarilumab dosing*

To allow comparison with the other ongoing clinical trial for hospitalized patients with COVID-19 (NCT04315298) a single dose of 400mg sarilumab will be administered.

6. DOMAIN OBJECTIVES

The objective of this domain is to determine the effectiveness of different immune modulation strategies for patients with severe pneumonia who have suspected or microbiological testing-confirmed COVID-19 infection.

We hypothesize that the probability of occurrence of the primary endpoint specified from the PAtC will differ based on the allocated immune modulation strategy. The following interventions will be available:

- No immune modulation for COVID-19 (no placebo)
- interferon-β1a (IFN-β1a)
- anakinra
- tocilizumab
- sarilumab

We hypothesize that the treatment effect of different immune modulation strategies is different depending on whether COVID-19 infection is confirmed to be present or absent.

We hypothesize that the treatment effect of different immune modulation strategies is different depending on biomarker strata status.

We hypothesize that the treatment effect of different immune modulation strategies is different depending on allocation status in the Corticosteroid Domain. This is a treatment-by-treatment interaction between the interventions in the COVID-19 Immune Modulation Domain and the Corticosteroid Domain. Based on post-hoc analyses of the INTEREST trial (Ranieri et al., 2020) regarding combination therapy with IFN-β1a and corticosteroids, this interaction will be evaluated with an informative prior that is negative.

We hypothesize that the treatment effect of different immune modulation strategies is different depending on allocation status in the COVID-19 Antiviral Therapy Domain. This is a treatment-by-treatment interaction between the interventions in the COVID-19 Antiviral Therapy Domain and the COVID-19 Immune Modulation Therapy Domain.

SUPERSEDED

Each participating site has the option to opt-in to two or more interventions to be included in the randomization schedule depending on local clinical preference, usual practice, acceptable practice, and the availability of the intervention at that site. At launch, and continuing at time of Version 2.0 participation in the 'no immune modulation for COVID-19 intervention' is mandatory.

7. TRIAL DESIGN

This domain will be conducted as part of the REMAP-CAP trial (see Core Protocol Section 7). Treatment allocation will be adaptive, as described in the Core Protocol Section 7.5.2 and from the PAtC.

7.1. *Population*

The REMAP enrolls patients with severe pneumonia admitted to ICU (see Core Protocol Section 7.3).

7.2. *Eligibility criteria*

Patients are eligible for this domain if they meet all of the platform-level inclusion and none of the platform-level exclusion criteria (see Core Protocol Section 7.4 and PAtC). Patients eligible for the REMAP may have conditions that exclude them from the COVID-19 Immune Modulation Domain.

7.2.1. Domain inclusion criteria

Patients are eligible for this domain if:

- COVID-19 infection is suspected by the treating clinician or has been confirmed by microbiological testing (i.e. PISOP stratum)
- Microbiological testing for SARS-CoV-2 of upper or lower respiratory tract secretions or both has occurred or is intended to occur

7.2.2. Domain exclusion criteria

Patients will be excluded from this domain if they have any of the following:

- More than 24 hours has elapsed since ICU admission
- Patient has already received any dose of one or more of any form of interferon, anakinra, tocilizumab, or sarilumab during this hospitalization or is on long-term therapy with any of these agents prior to this hospital admission

SUPERSEDED

- Known condition or treatment resulting in ongoing immune suppression including neutropenia prior to this hospitalization
- Patient has been randomized in a trial evaluating an immune modulation agent for proven or suspected COVID-19 infection, where the protocol of that trial requires ongoing administration of study drug
- The treating clinician believes that participation in the domain would not be in the best interests of the patient

7.2.3. Intervention exclusion criteria

Patients may also be excluded from receiving one or more interventions within the domain for patient-specific reasons. In such cases, patients will be randomly allocated a remaining intervention from among those available at that site.

Patients who are eligible for only a single intervention at a site (i.e. all other interventions are contraindicated) are not eligible for this domain. Patients in whom all interventions are contraindicated will be treated according to the current standard of care at the clinician's discretion.

- Known hypersensitivity to an agent specified as an intervention in this domain will exclude a patient from receiving that agent
-
- Intention to prescribe systemic corticosteroids for any reason, other than participation in the Corticosteroid Domain of this platform, will result in exclusion from receiving IFN-β1a
- Known hypersensitivity to proteins produced by E. coli will result in exclusion from receiving anakinra
- Known or suspected pregnancy will result in exclusion from the anakinra, IFN-β1a, tocilizumab, and sarilumab interventions. It is normal clinical practice that women admitted who are in an age group in which pregnancy is possible will have a pregnancy test conducted. The results of such tests will be used to determine interpretation of this exclusion criteria.
- A baseline alanine aminotransferase or an aspartate aminotransferase that is more than five times the upper limit of normal will result in exclusion from receiving tocilizumab or sarilumab
- A baseline platelet count < 50 x 10^9 / L will result in exclusion from receiving tocilizumab or sarilumab

SUPERSEDED

7.3. *Interventions*

7.3.1. Immune modulation interventions

Patients will be randomly assigned to receive one of the following open-label strategies. All interventions will be commenced immediately after allocation status is revealed.

☐ No immune modulation for COVID-19 (no placebo)

☐ interferon beta-1a (IFN-β1a)

☐ anakinra

☐ tocilizumab

☐ sarilumab

It is required that all sites will participate in the 'No immune modulation for COVID-19' intervention, and each site has the option to opt-in to one or more of the remaining interventions based on local practice and availability of the intervention.

7.3.2. IFN-β1a

7.3.2.1. Dosing

IFN-β1a 10 or 11 µg will be diluted in 4.5 mL of 0.9% saline. The diluted IFN-β1a will be administered as an intravenous bolus injection via a central or peripheral line. The injection will be followed with a 5 mL flush of sterile saline. IFN-β1a is available in Europe, Australia, North America, and Middle and South-East Asia as REBIF®.

The injection should be given at the same time each day, up to one hour either side of that time. If for any reason this is not possible, the treatment window may be extended by up to 4 hours. If study drug is not administered within this 4-hour period it should be omitted for that day and recommence the following day.

7.3.2.2. Duration of therapy

IFN-β1a will be administered once daily for 6 days or until ICU discharge, whichever occurs first.

SUPERSEDED

7.3.3. IL-1Ra

7.3.3.1. Dosing

Anakinra will be administered as an intravenous bolus injection via a central or peripheral line. A loading dose of 300 mg will be administered, followed by maintenance doses of 100 mg of anakinra administered every 6 hours. In patients with creatinine clearance of less than 30 ml/min or receiving renal replacement therapy, anakinra will be dosed every 12 hours.

7.3.3.2. Duration of therapy

Anakinra will be administered four times daily until the patient has been breathing without receiving invasive mechanical ventilation for more than 24 hours or for 14 days in patients who continue to receive invasive mechanical ventilation. For patients not receiving invasive mechanical ventilation, the drug will stop on ICU discharge or after 14 days, whichever occurs first.

7.3.4. Tocilizumab

7.3.4.1. Dosing

Tocilizumab will be administered at a dose of 8mg/kg based on measured or estimated body weight with total dose not exceeding 800mg. Tocilizumab will be administered as an intravenous infusion via a central or peripheral line over a one-hour period. The appropriate dose of drug will be mixed in a 100 ml bag of 0.9% saline, after removing an equivalent volume of saline, 0.4ml/kg, to match the added drug, so that the total volume is 100 mls. The infusion speed must be 10 mls per hour for 15minutes and then increased to 130 mls per hour for the next 45 minutes. After completion of the infusion of active study drug, at least 20 mls of 0.9% saline should be used to flush the drug through the giving set.

7.3.4.2. Duration of therapy

A single dose will be administered. If the treating clinician believe there has not been sufficient clinical improvement, repeat administration of the same dose can be administered between 12 and 24 hours after the initial dose.

7.3.5. Sarilumab

7.3.5.1. Dosing

Sarilumab will be administered as a single dose of 400 mg as an intravenous infusion via a central or peripheral line over a one-hour period. The drug will be mixed in a 100 ml bag of 0.9% saline, after removing an equivalent volume of saline so that the total volume is 100 mls. The infusion speed

SUPERSEDED

must be 10 mls per hour for 15 minutes and then increased to 130 mls per hour for the next 45 minutes. After completion of the infusion of active study drug, at least 20 mls of 0.9% saline should be used to flush the drug through the giving set.

7.3.5.2. Duration of therapy

A single dose of 400 mg will be administered.

7.3.6. Discontinuation of study drug

An immunomodulatory agent for COVID-19 infection should be discontinued if there is development of an SAE. Study drug can be discontinued at any time by the treating clinician if doing so is regarded as being in the best interests of the patient.

7.3.7. COVID-19 immune modulation strategy in patients negative for COVID-19 infection

In patients with suspected COVID-19 infection who receive an allocation status to receive any of the active interventions but who subsequently test negative for COVID-19 infection after allocation may have treatment ceased unless the treating clinician believes that doing so is not clinically appropriate. This decision should take into account the known or suspected sensitivity of testing for COVID-19 infection.

7.4. Concomitant care

Additional agents, other than those specified in the platform, that are intended to modulate the immune response against COVID-19 infection should not be administered. In patients who have received an allocation status in the Antibiotic Domain, and have microbiological testing confirmed COVID-19 infection, continuation of empiric anti-bacterial agents will be as per the Antibiotic Domain-Specific Appendix (Section 8.3). All treatment that is not specified by assignment within the platform will be determined by the treating clinician.

7.5. Endpoints

7.5.1. Primary endpoint

The primary endpoint for this domain is the primary outcome specified in an operational document from within the options specified from the PAtC.

7.5.2. Secondary endpoints

All secondary endpoints as specified from the PAtC Section 7.5.2.

SUPERSEDED

The domain-specific secondary outcome measures (occurring during the index hospitalization, censored 90 days after enrollment) will be:

- Serial detection of SARS-CoV-2 in upper or lower respiratory tract specimens (using only specimens collected for routine clinically indicated testing)
- SAE as defined in Core Protocol and this DSA below

8. TRIAL CONDUCT

8.1. *Microbiology*

Microbiological testing will be performed as per local practice, including bacterial and viral testing to guide clinical care. Results of these tests will be collected but no additional testing is specified in this protocol.

Sites that are participating in this domain are encouraged to also participate in the Clinical Characterization Protocol (CCP) for patients with COVID-19 that has been established by the International Severe Acute Respiratory and Emerging Infectious Consortium (https://isaric.tghn.org/CCP/). This protocol specifies the collection of biological samples from patients with COVID-19. If additional sampling is required, beyond that specified in the CCP, for example for studies of pharmacokinetics, this is permitted but will occur via separate protocol. Samples collected in patients who are enrolled in the CCP or other studies may be made available to REMAP-CAP investigators to evaluate aspects of host or pathogen biology associated with assignment in this domain. Ethical approval at such sites and agreement from patients to undertake the CCP and other studies will be obtained separately.

8.2. *Domain-specific data collection*

8.2.1. Clinical data collection

Additional domain-specific data will be collected.

- Administration of systemic corticosteroids
- Administration of antiviral agents intended to be active against COVID-19 infection
- Administration of immune modulatory agents intended to influence host response to COVID-19 infection

SUPERSEDED

8.3. *Criteria for discontinuation*

Refer to Core Protocol Section 8.7 for criteria for discontinuation of participation in the REMAP-CAP trial.

8.4. *Blinding*

8.4.1. Blinding

All medication will be administered on an open-label basis.

8.4.2. Unblinding

Not relevant.

9. STATISTICAL CONSIDERATIONS

9.1. *Domain-specific stopping rules*

If a Platform conclusion of equivalence in the primary endpoint is demonstrated, the DSMB and the ITSC may consider continuation of randomization if clinically relevant differences in secondary endpoints have not been demonstrated and it is considered plausible that clinically relevant differences in one or more secondary endpoints may be capable of being demonstrated. In all other respects the stopping rules for this domain are those outlined in the Core Protocol Section and from the PAtC.

9.2. *Unit-of-analysis and strata*

The default unit-of-analysis, for both analysis of treatment effect and the Response Adaptive Randomization, will be the PISOP stratum, as specified from the PAtC. As determined by the ITSC, and based on an understanding of the sensitivity and availability of testing for COVID-19 infection, the unit-of analysis may be modified to allow separate analysis of the COVID-19 infection confirmed and not confirmed stratum. This will be an operational decision.

An additional strata may be applied to the unit-of-analysis which will be determined by status with respect to one or more biomarkers comprising d-dimer, ferritin, C-reactive protein, neutrophil count, and lymphocyte count collected from samples collected closest to the time of randomization and not more than 2 hours after randomization. The exact biomarker strata structure will be specified in an operational document that will be finalized before the first interim analysis that applies the

SUPERSEDED

biomarker strata. It is believed that there is insufficient information regarding the biology of the disease to set the composition, categories and thresholds for this biomarker at the time of this amendment.

At the time of a Platform Conclusion, results will be reported for all randomized patients, patients in whom COVID-19 infection is confirmed by microbiological testing, microbiological tests do not detect or isolate COVID-19 infection, and testing is not performed.

The shock strata will not contribute to unit-of-analysis for this domain, as this strata is not applied in the Pandemic Statistical Model.

The influenza strata will not contribute to unit-of-analysis for this domain.

9.3. *Timing of revealing of randomization status*

The timing of the revealing of allocation status and administration of interventions is specified to be Randomization with Immediate Reveal and Initiation or Randomization with Deferred Reveal if prospective agreement to participate is required for this domain (see section 7.8.3.6 in Core Protocol)

9.4. *Interactions with interventions in other domains*

An *a priori* interaction with the Antibiotic Domain is not able to be evaluated as analysis occurs in different statistical models.

An *a priori* interaction with the Macrolide Duration Domain is not considered possible and will not be incorporated into the statistical models used to analyze this domain.

An *a priori* interaction with the Antiviral Domain is not able to be evaluated as analysis occurs in different statistical models.

An *a priori* interaction with the Corticosteroid Domain is considered possible and will be incorporated into the statistical models used to analyze this domain. The statistical model will specify an informative prior for interaction between assignment in the Corticosteroid Domain and assignment to IFN-β1a. For example, the informative prior specifies a negative effect of the interaction, with prior median of a 50% reduction in the odds ratio. The median of this prior on the odds ratio scale is equal to 0.5, the mean is equal to 0.824, and 75% of the mass of the prior falls below 1. The 95% prior credible interval is between 0.07 and 3.44. With this negative prior, and

SUPERSEDED

assuming the negative interaction is real between IFN-β1a and the fixed-duration hydrocortisone intervention, a mean of 20 patients are exposed to this combination before the regimen containing both interventions is dropped from the platform (see Appendix 1). The strength of the negative prior is conservative with respect to the published and unpublished results of the INTEREST trial (Ranieri et al., 2020).

If a harmful interaction is present, the use of an informative prior that is negative reduces the number of patients exposed to this combination. If a negative interaction is confirmed, the combination is eliminated rapidly from the randomization schedule. If a negative interaction is not supported by accruing data, the frequency with which this combination will occur will rise progressively to allow evaluation of potential synergy. The use of an informative prior that is negative, may increase slightly the likelihood of a type I error for the interaction (i.e. concluding that the combination is harmful when the combination is not harmful) (see Appendix 1). Nevertheless, based on prior data, the use of an informative prior is prudent to reduce risk of harm from the study (noting that combination therapy is likely, or more likely, in patients not participating in the platform). For the purposes of analysis and reporting, the combination of IFN-β1a with interventions in the Corticosteroid Domain are pre-specified to be an 'intervention' so that identification of interaction between IFN-β1a and either of the interventions that involve administration of corticosteroids will be reported as a conclusion from the study. Simulations used to evaluate the interaction under a variety of scenarios are included in Appendix 1.

An *a priori* interaction with the COVID-19 Antiviral Therapy Domain is considered possible and will be incorporated into the statistical models used to analyze this domain. An interaction may exist between IFN-β1a and antiviral treatment. For the purposes of analysis and reporting this combination is pre-specified to be an 'intervention' i.e. superiority, or inferiority, of the combination can be reported as a conclusion from the study.

No interaction is evaluable between the Ventilation Domain and this domain.

9.5. *Nesting of interventions*

There is one nest within this domain, comprising tocilizumab and sarilumab. The rationale for this is that the mechanism of action of these agents is similar.

SUPERSEDED

9.6. *Threshold probability for superiority and inferiority*

The threshold odds ratio delta for superiority and inferiority in this domain are those specified in the Operating Characteristics document derived from the PAtC. It is noted that the threshold for superiority and inferiority in the current model has been modified from 0.95 to 0.99 to provide adequate control of type I error, following the evaluation of simulations. It is also noted that asymmetric probabilities may be specified for harm, to allow early cessation and declaration of a Platform Conclusion for interventions that are unlikely to be effective and may be harmful. If so, this will be specified in the Operating Characteristics document which is placed in the public domain.

It is also noted that the requirements for declaration of a Platform Conclusion for inferiority for the 'no immune modulation' intervention is modified so that such a declaration will be made if this intervention is inferior to one or more active interventions. The consequence of this is that the 'no immune modulation' intervention no longer needs to be inferior to all other interventions before a Platform conclusion is reached. This is necessary because of the increasing number of interventions within the domain and serves to remove the 'no immune modulation' intervention at the earliest possible time, if there are one or more effective interventions within the domain.

9.7. *Threshold odds ratio delta for equivalence*

The threshold odds ratio delta for equivalence in this domain is that specified from the PAtC (Section 7.8.8).

9.8. *Informative priors*

This domain will launch with priors that are not informative for main effects. As outlined previously, an informative prior will be used for specified interactions with the Corticosteroid Domain. If new immune modulation agents are added to the domain, consideration will be given to the use of informative priors at the time of amendment of the DSA.

9.9. *Post-trial sub-groups*

Domain-specific post-hoc sub-groups will be used in analysis following the conclusion of one or more interventions within the domain. The *a priori* patient sub-groups of interest are:

SUPERSEDED

- Proven concomitant bacterial co-infection, defined as having isolation or detection of a known pathogen that causes CAP from blood, pleural fluid, or lower respiratory tract specimen.
- Shock strata
- Influenza strata
- Receiving invasive mechanical ventilation at baseline
- All remaining potentially evaluable treatment-by-treatment interactions with other domains

10.ETHICAL CONSIDERATIONS

10.1. *Data Safety and Monitoring Board*

The DSMB should be aware that the superiority, inferiority, or equivalence of different interventions with respect to the primary endpoint is possible, and if equivalence is demonstrated, determination of the optimal intervention may be based on secondary endpoints. The DSMB will be informed specifically regarding the evaluation of interaction between the Corticosteroid domain and IFN-β1a.

The DSMB should take into account the public health, as well as clinical significance, of the analyses of this domain and are empowered to discuss results with relevant international and national public health authorities, with rapid dissemination of results to the larger community being the goal.

Safety secondary outcomes will be reported to the DSMB who are empowered to require additional analyses regarding these outcomes as required.

10.2. *Potential domain-specific adverse events*

For patients assigned to IFN-β1a occurrence of any of the following should be reported as an SAE

- Unexplained pulmonary hypertension or right ventricular failure or both
- New onset autoimmune disease (such as thyroiditis, type-I diabetes)
- Severe neutropenia, out of keeping with clinical disease
- Severe thrombocytopenia out of keeping with clinical disease

For patients assigned to anakinra

- Severe neutropenia, out of keeping with clinical disease
- Severe thrombocytopenia out of keeping with clinical disease
- Allergic reactions, including anaphylactic reactions and angio-edema

SUPERSEDED

For patients assigned to tocilizumab or sarilumab

- Severe thrombocytopenia, out of keeping with clinical disease
- Severe neutropenia, out of keeping with clinical disease
- Increase in LFTs to 5x upper limit of normal
- Gastrointestinal perforation
- Allergic reactions, including anaphylactic reactions and angio-edema
- Secondary opportunistic infection, out of keeping with clinical disease

Other SAEs should be reported only where, in the opinion of the site-investigator, the event might reasonably have occurred as a consequence of a study intervention or study participation (see Core Protocol Section 8.13).

10.3. *Domain-specific consent issues*

As noted in the background, and endorsed by the WHO, in the absence of evidence of effectiveness of an immune modulatory agent for COVID-19, the use of a no treatment control is both appropriate and ethical.

The default for this domain is that randomization would require prospective agreement, either from the participant or a participant's authorized representative. Where prospective agreement is required, a period of up to 24 hours from the time of establishing eligibility will be available to obtain agreement and commence the assigned therapy. In such situations allocation status will not be revealed until prospective agreement has been obtained.

During a pandemic, visiting by relatives of affected patients may not be possible. In such situations, alternative methods for confirming consent including electronic and telephone communication, as permitted by an appropriate ethical review body, may be acceptable methods for confirming agreement to participate in this (and other) domains of the platform.

11. GOVERNANCE ISSUES

11.1. *Funding of domain*

Funding sources for the REMAP-CAP trial are specified in the Core Protocol Section 2.5. This domain has not received any additional domain-specific funding but such funding, from any source, may be obtained during the life-time of the domain.

SUPERSEDED

11.2. Funding of domain interventions and outcome measures

The supply and cost re-imbursement of the drugs will be vary by region and possibly even within different countries in a region. The options will include purchase of the drugs by individual recruiting sites or centrally by health bodies. In some cases, the drugs may be donated for trial use by the relevant company (e.g. Roche UK have donated the tocilizumab for use in the UK). In other cases, the costs may be reimbursed by other companies (e.g. Faron Pharmaceuticals for the cost or supply of IFN-β1a). These companies will have no role in the design, conduct, analysis, or reporting of this domain. Costs unrelated to study drug may be sought from one or more of pending grant applications, Faron Pharmaceuticals and SOBI.

11.3. Domain-specific declarations of interest

All investigators involved in REMAP-CAP maintain a registry of interests on the REMAP-CAP website. These are updated periodically and publicly accessible on the study website.

SUPERSEDED

12. REFERENCES

ARABI, Y. M., ALOTHMAN, A., BALKHY, H. H., AL-DAWOOD, A., ALJOHANI, S., AL HARBI, S., KOJAN, S., AL JERAISY, M., DEEB, A. M., ASSIRI, A. M., AL-HAMEED, F., ALSAEDI, A., MANDOURAH, Y., ALMEKHLAFI, G. A., SHERBEENI, N. M., ELZEIN, F. E., MEMON, J., TAHA, Y., ALMOTAIRI, A., MAGHRABI, K. A., QUSHMAQ, I., AL BSHABSHE, A., KHARABA, A., SHALHOUB, S., JOSE, J., FOWLER, R. A., HAYDEN, F. G., HUSSEIN, M. A. & AND THE, M. T. G. 2018. Treatment of Middle East Respiratory Syndrome with a combination of lopinavir-ritonavir and interferon-beta1b (MIRACLE trial): study protocol for a randomized controlled trial. *Trials,* 19**,** 81.

ARABI, Y. M., BALKHY, H. H., HAYDEN, F. G., BOUCHAMA, A., LUKE, T., BAILLIE, J. K., AL-OMARI, A., HAJEER, A. H., SENGA, M., DENISON, M. R., NGUYEN-VAN-TAM, J. S., SHINDO, N., BERMINGHAM, A., CHAPPELL, J. D., VAN KERKHOVE, M. D. & FOWLER, R. A. 2017. Middle East Respiratory Syndrome. *N Engl J Med,* 376**,** 584-594.

AREND, W. P., WELGUS, H. G., THOMPSON, R. C. & EISENBERG, S. P. 1990. Biological properties of recombinant human monocyte-derived interleukin 1 receptor antagonist. *J Clin Invest,* 85**,** 1694-7.

BAAS, T., TAUBENBERGER, J. K., CHONG, P. Y., CHUI, P. & KATZE, M. G. 2006. SARS-CoV virus-host interactions and comparative etiologies of acute respiratory distress syndrome as determined by transcriptional and cytokine profiling of formalin-fixed paraffin-embedded tissues. *J Interferon Cytokine Res,* 26**,** 309-17.

BELLINGAN, G., MAKSIMOW, M., HOWELL, D. C., STOTZ, M., BEALE, R., BEATTY, M., WALSH, T., BINNING, A., DAVIDSON, A., KUPER, M., SHAH, S., COOPER, J., WARIS, M., YEGUTKIN, G. G., JALKANEN, J., SALMI, M., PIIPPO, I., JALKANEN, M., MONTGOMERY, H. & JALKANEN, S. 2014. The effect of intravenous interferon-beta-1a (FP-1201) on lung CD73 expression and on acute respiratory distress syndrome mortality: an open-label study. *Lancet Respir Med,* 2**,** 98-107.

BOOTH, C. M., MATUKAS, L. M., TOMLINSON, G. A., RACHLIS, A. R., ROSE, D. B., DWOSH, H. A., WALMSLEY, S. L., MAZZULLI, T., AVENDANO, M., DERKACH, P., EPHTIMIOS, I. E., KITAI, I., MEDERSKI, B. D., SHADOWITZ, S. B., GOLD, W. L., HAWRYLUCK, L. A., REA, E., CHENKIN, J. S., CESCON, D. W., POUTANEN, S. M. & DETSKY, A. S. 2003. Clinical features and short-term outcomes of 144 patients with SARS in the greater Toronto area. *JAMA,* 289**,** 2801-9.

CANNA, S. W., DE JESUS, A. A., GOUNI, S., BROOKS, S. R., MARRERO, B., LIU, Y., DIMATTIA, M. A., ZAAL, K. J., SANCHEZ, G. A., KIM, H., CHAPELLE, D., PLASS, N., HUANG, Y., VILLARINO, A. V., BIANCOTTO, A., FLEISHER, T. A., DUNCAN, J. A., O'SHEA, J. J., BENSELER, S., GROM, A., DENG, Z., LAXER, R. M. & GOLDBACH-MANSKY, R. 2014. An activating NLRC4 inflammasome mutation causes autoinflammation with recurrent macrophage activation syndrome. *Nat Genet,* 46**,** 1140-6.

CAVALLI, G. & DINARELLO, C. A. 2018. Anakinra Therapy for Non-cancer Inflammatory Diseases. *Front Pharmacol,* 9**,** 1157.

CHAN, J. F., CHAN, K. H., KAO, R. Y., TO, K. K., ZHENG, B. J., LI, C. P., LI, P. T., DAI, J., MOK, F. K., CHEN, H., HAYDEN, F. G. & YUEN, K. Y. 2013. Broad-spectrum antivirals for the emerging Middle East respiratory syndrome coronavirus. *J Infect,* 67**,** 606-16.

CHAN, J. F., YAO, Y., YEUNG, M. L., DENG, W., BAO, L., JIA, L., LI, F., XIAO, C., GAO, H., YU, P., CAI, J. P., CHU, H., ZHOU, J., CHEN, H., QIN, C. & YUEN, K. Y. 2015. Treatment With Lopinavir/Ritonavir or Interferon-beta1b Improves Outcome of MERS-CoV Infection in a Nonhuman Primate Model of Common Marmoset. *J Infect Dis,* 212**,** 1904-13.

SUPERSEDED

CHEN, N., ZHOU, M., DONG, X., QU, J., GONG, F., HAN, Y., QIU, Y., WANG, J., LIU, Y., WEI, Y., XIA, J., YU, T., ZHANG, X. & ZHANG, L. 2020. Epidemiological and clinical characteristics of 99 cases of 2019 novel coronavirus pneumonia in Wuhan, China: a descriptive study. *Lancet,* 395**,** 507-513.

DE WILDE, A. H., RAJ, V. S., OUDSHOORN, D., BESTEBROER, T. M., VAN NIEUWKOOP, S., LIMPENS, R. W., POSTHUMA, C. C., VAN DER MEER, Y., BARCENA, M., HAAGMANS, B. L., SNIJDER, E. J. & VAN DEN HOOGEN, B. G. 2013. MERS-coronavirus replication induces severe in vitro cytopathology and is strongly inhibited by cyclosporin A or interferon-alpha treatment. *J Gen Virol,* 94**,** 1749-60.

FAURE, E., POISSY, J., GOFFARD, A., FOURNIER, C., KIPNIS, E., TITECAT, M., BORTOLOTTI, P., MARTINEZ, L., DUBUCQUOI, S., DESSEIN, R., GOSSET, P., MATHIEU, D. & GUERY, B. 2014. Distinct immune response in two MERS-CoV-infected patients: can we go from bench to bedside? *PLoS One,* 9**,** e88716.

FISHER, C. J., JR., DHAINAUT, J. F., OPAL, S. M., PRIBBLE, J. P., BALK, R. A., SLOTMAN, G. J., IBERTI, T. J., RACKOW, E. C., SHAPIRO, M. J., GREENMAN, R. L. & ET AL. 1994. Recombinant human interleukin 1 receptor antagonist in the treatment of patients with sepsis syndrome. Results from a randomized, double-blind, placebo-controlled trial. Phase III rhIL-1ra Sepsis Syndrome Study Group. *JAMA,* 271**,** 1836-43.

FRIEMAN, M., HEISE, M. & BARIC, R. 2008. SARS coronavirus and innate immunity. *Virus Res,* 133**,** 101-12.

GENOVESE, M. C., FLEISCHMANN, R., KIVITZ, A. J., RELL-BAKALARSKA, M., MARTINCOVA, R., FIORE, S., ROHANE, P., VAN HOOGSTRATEN, H., GARG, A., FAN, C., VAN ADELSBERG, J., WEINSTEIN, S. P., GRAHAM, N. M., STAHL, N., YANCOPOULOS, G. D., HUIZINGA, T. W. & VAN DER HEIJDE, D. 2015. Sarilumab Plus Methotrexate in Patients With Active Rheumatoid Arthritis and Inadequate Response to Methotrexate: Results of a Phase III Study. *Arthritis Rheumatol,* 67**,** 1424-37.

GOTTESMAN, M. H. & FRIEDMAN-UREVICH, S. 2006. Interferon beta-1b (betaseron/betaferon) is well tolerated at a dose of 500 microg: interferon dose escalation assessment of safety (IDEAS). *Mult Scler,* 12**,** 271-80.

GRALINSKI, L. E., SHEAHAN, T. P., MORRISON, T. E., MENACHERY, V. D., JENSEN, K., LEIST, S. R., WHITMORE, A., HEISE, M. T. & BARIC, R. S. 2018. Complement Activation Contributes to Severe Acute Respiratory Syndrome Coronavirus Pathogenesis. *mBio,* 9.

GROM, A. A., HORNE, A. & DE BENEDETTI, F. 2016. Macrophage activation syndrome in the era of biologic therapy. *Nat Rev Rheumatol,* 12**,** 259-68.

GUAN, W. J., NI, Z. Y., HU, Y., LIANG, W. H., OU, C. Q., HE, J. X., LIU, L., SHAN, H., LEI, C. L., HUI, D. S. C., DU, B., LI, L. J., ZENG, G., YUEN, K. Y., CHEN, R. C., TANG, C. L., WANG, T., CHEN, P. Y., XIANG, J., LI, S. Y., WANG, J. L., LIANG, Z. J., PENG, Y. X., WEI, L., LIU, Y., HU, Y. H., PENG, P., WANG, J. M., LIU, J. Y., CHEN, Z., LI, G., ZHENG, Z. J., QIU, S. Q., LUO, J., YE, C. J., ZHU, S. Y., ZHONG, N. S. & CHINA MEDICAL TREATMENT EXPERT GROUP FOR, C. 2020. Clinical Characteristics of Coronavirus Disease 2019 in China. *N Engl J Med*.

HARMS, P. W., SCHMIDT, L. A., SMITH, L. B., NEWTON, D. W., PLETNEVA, M. A., WALTERS, L. L., TOMLINS, S. A., FISHER-HUBBARD, A., NAPOLITANO, L. M., PARK, P. K., BLAIVAS, M., FANTONE, J., MYERS, J. L. & JENTZEN, J. M. 2010. Autopsy findings in eight patients with fatal H1N1 influenza. *Am J Clin Pathol,* 134**,** 27-35.

HART, B. J., DYALL, J., POSTNIKOVA, E., ZHOU, H., KINDRACHUK, J., JOHNSON, R. F., OLINGER, G. G., JR., FRIEMAN, M. B., HOLBROOK, M. R., JAHRLING, P. B. & HENSLEY, L. 2014. Interferon-beta

SUPERSEDED

and mycophenolic acid are potent inhibitors of Middle East respiratory syndrome coronavirus in cell-based assays. *J Gen Virol,* 95**,** 571-7.

HIRUMA, T., TSUYUZAKI, H., UCHIDA, K., TRAPNELL, B. C., YAMAMURA, Y., KUSAKABE, Y., TOTSU, T., SUZUKI, T., MORITA, S., DOI, K., NOIRI, E., NAKAMURA, K., NAKAJIMA, S., YAHAGI, N., MORIMURA, N., CHANG, K. & YAMADA, Y. 2018. IFN-beta Improves Sepsis-related Alveolar Macrophage Dysfunction and Postseptic Acute Respiratory Distress Syndrome-related Mortality. *Am J Respir Cell Mol Biol,* 59**,** 45-55.

HUANG, C., WANG, Y., LI, X., REN, L., ZHAO, J., HU, Y., ZHANG, L., FAN, G., XU, J., GU, X., CHENG, Z., YU, T., XIA, J., WEI, Y., WU, W., XIE, X., YIN, W., LI, H., LIU, M., XIAO, Y., GAO, H., GUO, L., XIE, J., WANG, G., JIANG, R., GAO, Z., JIN, Q., WANG, J. & CAO, B. Clinical features of patients infected with 2019 novel coronavirus in Wuhan, China. *The Lancet*.

HUANG, C., WANG, Y., LI, X., REN, L., ZHAO, J., HU, Y., ZHANG, L., FAN, G., XU, J., GU, X., CHENG, Z., YU, T., XIA, J., WEI, Y., WU, W., XIE, X., YIN, W., LI, H., LIU, M., XIAO, Y., GAO, H., GUO, L., XIE, J., WANG, G., JIANG, R., GAO, Z., JIN, Q., WANG, J. & CAO, B. 2020. Clinical features of patients infected with 2019 novel coronavirus in Wuhan, China. *Lancet,* 395**,** 497-506.

HUIZINGA, T. W., FLEISCHMANN, R. M., JASSON, M., RADIN, A. R., VAN ADELSBERG, J., FIORE, S., HUANG, X., YANCOPOULOS, G. D., STAHL, N. & GENOVESE, M. C. 2014. Sarilumab, a fully human monoclonal antibody against IL-6Ralpha in patients with rheumatoid arthritis and an inadequate response to methotrexate: efficacy and safety results from the randomised SARIL-RA-MOBILITY Part A trial. *Ann Rheum Dis,* 73**,** 1626-34.

HURWITZ, B. J., JEFFERY, D., ARNASON, B., BIGLEY, K., COYLE, P., GOODIN, D., KABA, S., KIRZINGER, S., LYNCH, S., MANDLER, R., MIKOL, D., RAMMOHAN, K., SATER, R., SRIRAM, S., THROWER, B., BOATENG, F., JAKOBS, P., WASH, M. B. & BOGUMIL, T. 2008. Tolerability and safety profile of 12- to 28-week treatment with interferon beta-1b 250 and 500 microg QOD in patients with relapsing-remitting multiple sclerosis: a multicenter, randomized, double-blind, parallel-group pilot study. *Clin Ther,* 30**,** 1102-12.

JACQUES, A., BLEAU, C., TURBIDE, C., BEAUCHEMIN, N. & LAMONTAGNE, L. 2009. Macrophage interleukin-6 and tumour necrosis factor-alpha are induced by coronavirus fixation to Toll-like receptor 2/heparan sulphate receptors but not carcinoembryonic cell adhesion antigen 1a. *Immunology,* 128**,** e181-92.

JANKOVIC, S. M. 2010. Injectable interferon beta-1b for the treatment of relapsing forms of multiple sclerosis. *J Inflamm Res,* 3**,** 25-31.

JUNE, R. R. & OLSEN, N. J. 2016. Room for more IL-6 blockade? Sarilumab for the treatment of rheumatoid arthritis. *Expert Opin Biol Ther,* 16**,** 1303-9.

KISS, J., YEGUTKIN, G. G., KOSKINEN, K., SAVUNEN, T., JALKANEN, S. & SALMI, M. 2007. IFN-beta protects from vascular leakage via up-regulation of CD73. *Eur J Immunol,* 37**,** 3334-8.

KNAUS, W. A., HARRELL, F. E., JR., LABRECQUE, J. F., WAGNER, D. P., PRIBBLE, J. P., DRAPER, E. A., FISHER, C. J., JR. & SOLL, L. 1996. Use of predicted risk of mortality to evaluate the efficacy of anticytokine therapy in sepsis. The rhIL-1ra Phase III Sepsis Syndrome Study Group. *Crit Care Med,* 24**,** 46-56.

LANDONI, G., COMIS, M., CONTE, M., FINCO, G., MUCCHETTI, M., PATERNOSTER, G., PISANO, A., RUGGERI, L., ALVARO, G., ANGELONE, M., BERGONZI, P. C., BOCCHINO, S., BORGHI, G., BOVE, T., BUSCAGLIA, G., CABRINI, L., CALLEGHER, L., CARAMELLI, F., COLOMBO, S., CORNO, L., DEL SARTO, P., FELTRACCO, P., FORTI, A., GANZAROLI, M., GRECO, M., GUARRACINO, F., LEMBO, R., LOBREGLIO, R., MERONI, R., MONACO, F., MUSU, M., PALA, G., PASIN, L., PIERI, M., PISARRA, S., PONTICELLI, G., ROASIO, A., SANTINI, F., SILVETTI, S., SZEKELY, A., ZAMBON,

SUPERSEDED

M., ZUCCHETTI, M. C., ZANGRILLO, A. & BELLOMO, R. 2015. Mortality in Multicenter Critical Care Trials: An Analysis of Interventions With a Significant Effect. *Crit Care Med,* 43**,** 1559-68.

LAU, S. K., LAU, C. C., CHAN, K. H., LI, C. P., CHEN, H., JIN, D. Y., CHAN, J. F., WOO, P. C. & YUEN, K. Y. 2013. Delayed induction of proinflammatory cytokines and suppression of innate antiviral response by the novel Middle East respiratory syndrome coronavirus: implications for pathogenesis and treatment. *J Gen Virol,* 94**,** 2679-90.

LEE, D. W., SANTOMASSO, B. D., LOCKE, F. L., GHOBADI, A., TURTLE, C. J., BRUDNO, J. N., MAUS, M. V., PARK, J. H., MEAD, E., PAVLETIC, S., GO, W. Y., ELDJEROU, L., GARDNER, R. A., FREY, N., CURRAN, K. J., PEGGS, K., PASQUINI, M., DIPERSIO, J. F., VAN DEN BRINK, M. R. M., KOMANDURI, K. V., GRUPP, S. A. & NEELAPU, S. S. 2019. ASTCT Consensus Grading for Cytokine Release Syndrome and Neurologic Toxicity Associated with Immune Effector Cells. *Biol Blood Marrow Transplant,* 25**,** 625-638.

LEW, T. W., KWEK, T. K., TAI, D., EARNEST, A., LOO, S., SINGH, K., KWAN, K. M., CHAN, Y., YIM, C. F., BEK, S. L., KOR, A. C., YAP, W. S., CHELLIAH, Y. R., LAI, Y. C. & GOH, S. K. 2003. Acute respiratory distress syndrome in critically ill patients with severe acute respiratory syndrome. *JAMA,* 290**,** 374-80.

MIN, C. K., CHEON, S., HA, N. Y., SOHN, K. M., KIM, Y., AIGERIM, A., SHIN, H. M., CHOI, J. Y., INN, K. S., KIM, J. H., MOON, J. Y., CHOI, M. S., CHO, N. H. & KIM, Y. S. 2016. Comparative and kinetic analysis of viral shedding and immunological responses in MERS patients representing a broad spectrum of disease severity. *Sci Rep,* 6**,** 25359.

MOULD, K. J. & JANSSEN, W. J. 2018. Recombinant IFN-beta for Postseptic Acute Lung Injury-What's the Mechanism? *Am J Respir Cell Mol Biol,* 59**,** 1-2.

NICHOLLS, J. M., POON, L. L., LEE, K. C., NG, W. F., LAI, S. T., LEUNG, C. Y., CHU, C. M., HUI, P. K., MAK, K. L., LIM, W., YAN, K. W., CHAN, K. H., TSANG, N. C., GUAN, Y., YUEN, K. Y. & PEIRIS, J. S. 2003. Lung pathology of fatal severe acute respiratory syndrome. *Lancet,* 361**,** 1773-8.

OPAL, S. M., FISHER, C. J., JR., DHAINAUT, J. F., VINCENT, J. L., BRASE, R., LOWRY, S. F., SADOFF, J. C., SLOTMAN, G. J., LEVY, H., BALK, R. A., SHELLY, M. P., PRIBBLE, J. P., LABRECQUE, J. F., LOOKABAUGH, J., DONOVAN, H., DUBIN, H., BAUGHMAN, R., NORMAN, J., DEMARIA, E., MATZEL, K., ABRAHAM, E. & SENEFF, M. 1997. Confirmatory interleukin-1 receptor antagonist trial in severe sepsis: a phase III, randomized, double-blind, placebo-controlled, multicenter trial. The Interleukin-1 Receptor Antagonist Sepsis Investigator Group. *Crit Care Med,* 25**,** 1115-24.

PARK, J. H., ROMERO, F. A., TAUR, Y., SADELAIN, M., BRENTJENS, R. J., HOHL, T. M. & SEO, S. K. 2018. Cytokine Release Syndrome Grade as a Predictive Marker for Infections in Patients With Relapsed or Refractory B-Cell Acute Lymphoblastic Leukemia Treated With Chimeric Antigen Receptor T Cells. *Clin Infect Dis,* 67**,** 533-540.

PEIRIS, J. S., CHU, C. M., CHENG, V. C., CHAN, K. S., HUNG, I. F., POON, L. L., LAW, K. I., TANG, B. S., HON, T. Y., CHAN, C. S., CHAN, K. H., NG, J. S., ZHENG, B. J., NG, W. L., LAI, R. W., GUAN, Y., YUEN, K. Y. & GROUP, H. U. S. S. 2003. Clinical progression and viral load in a community outbreak of coronavirus-associated SARS pneumonia: a prospective study. *Lancet,* 361**,** 1767-72.

RANIERI, V. M., PETTILA, V., KARVONEN, M. K., JALKANEN, J., NIGHTINGALE, P., BREALEY, D., MANCEBO, J., FERRER, R., MERCAT, A., PATRONITI, N., QUINTEL, M., VINCENT, J. L., OKKONEN, M., MEZIANI, F., BELLANI, G., MACCALLUM, N., CRETEUR, J., KLUGE, S., ARTIGAS-RAVENTOS, A., MAKSIMOW, M., PIIPPO, I., ELIMA, K., JALKANEN, S., JALKANEN, M., BELLINGAN, G. & GROUP, I. S. 2020. Effect of Intravenous Interferon beta-1a on Death and

SUPERSEDED

Days Free From Mechanical Ventilation Among Patients With Moderate to Severe Acute Respiratory Distress Syndrome: A Randomized Clinical Trial. *JAMA*.

RIGANTE, D., EMMI, G., FASTIGGI, M., SILVESTRI, E. & CANTARINI, L. 2015. Macrophage activation syndrome in the course of monogenic autoinflammatory disorders. *Clin Rheumatol,* 34**,** 1333-9.

SCHULERT, G. S. & GROM, A. A. 2015. Pathogenesis of macrophage activation syndrome and potential for cytokine- directed therapies. *Annu Rev Med,* 66**,** 145-59.

SCHULERT, G. S., ZHANG, M., FALL, N., HUSAMI, A., KISSELL, D., HANOSH, A., ZHANG, K., DAVIS, K., JENTZEN, J. M., NAPOLITANO, L., SIDDIQUI, J., SMITH, L. B., HARMS, P. W., GROM, A. A. & CRON, R. Q. 2016. Whole-Exome Sequencing Reveals Mutations in Genes Linked to Hemophagocytic Lymphohistiocytosis and Macrophage Activation Syndrome in Fatal Cases of H1N1 Influenza. *J Infect Dis,* 213**,** 1180-8.

SHAKOORY, B., CARCILLO, J. A., CHATHAM, W. W., AMDUR, R. L., ZHAO, H., DINARELLO, C. A., CRON, R. Q. & OPAL, S. M. 2016. Interleukin-1 Receptor Blockade Is Associated With Reduced Mortality in Sepsis Patients With Features of Macrophage Activation Syndrome: Reanalysis of a Prior Phase III Trial. *Crit Care Med,* 44**,** 275-81.

SMITS, S. L., DE LANG, A., VAN DEN BRAND, J. M., LEIJTEN, L. M., VAN, I. W. F., EIJKEMANS, M. J., VAN AMERONGEN, G., KUIKEN, T., ANDEWEG, A. C., OSTERHAUS, A. D. & HAAGMANS, B. L. 2010. Exacerbated innate host response to SARS-CoV in aged non-human primates. *PLoS Pathog,* 6**,** e1000756.

WANG, D., HU, B., HU, C., ZHU, F., LIU, X., ZHANG, J., WANG, B., XIANG, H., CHENG, Z., XIONG, Y., ZHAO, Y., LI, Y., WANG, X. & PENG, Z. 2020a. Clinical Characteristics of 138 Hospitalized Patients With 2019 Novel Coronavirus-Infected Pneumonia in Wuhan, China. *JAMA*.

WANG, D., HU, B., HU, C., ZHU, F., LIU, X., ZHANG, J., WANG, B., XIANG, H., CHENG, Z., XIONG, Y., ZHAO, Y., LI, Y., WANG, X. & PENG, Z. 2020b. Clinical Characteristics of 138 Hospitalized Patients With 2019 Novel Coronavirus–Infected Pneumonia in Wuhan, China. *JAMA*.

WEBB, S. A. 2015. Putting Critical Care Medicine on Trial. *Crit Care Med,* 43**,** 1767-8.

WU, Z. & MCGOOGAN, J. M. 2020. Characteristics of and Important Lessons From the Coronavirus Disease 2019 (COVID-19) Outbreak in China: Summary of a Report of 72314 Cases From the Chinese Center for Disease Control and Prevention. *JAMA*.

ZHOU, F., YU, T., DU, R., FAN, G., LIU, Y., LIU, Z., XIANG, J., WANG, Y., SONG, B., GU, X., GUAN, L., WEI, Y., LI, H., WU, X., XU, J., TU, S., ZHANG, Y., CHEN, H. & CAO, B. 2020a. Clinical course and risk factors for mortality of adult inpatients with COVID-19 in Wuhan, China: a retrospective cohort study. *Lancet,* 395**,** 1054-1062.

ZHOU, Y., FU, B., ZHENG, X., WANG, D., ZHAO, C., QI, Y., SUN, R., TIAN, Z., XU, X. & WEI, H. 2020b. Pathogenic T cells and inflammatory monocytes incite inflammatory storm in severe COVID-19 patients. *National Science Review***,** nwaa041.

ZHU, N., ZHANG, D., WANG, W., LI, X., YANG, B., SONG, J., ZHAO, X., HUANG, B., SHI, W., LU, R., NIU, P., ZHAN, F., MA, X., WANG, D., XU, W., WU, G., GAO, G. F., TAN, W., CHINA NOVEL CORONAVIRUS, I. & RESEARCH, T. 2020. A Novel Coronavirus from Patients with Pneumonia in China, 2019. *N Engl J Med*.

ZIELECKI, F., WEBER, M., EICKMANN, M., SPIEGELBERG, L., ZAKI, A. M., MATROSOVICH, M., BECKER, S. & WEBER, F. 2013. Human cell tropism and innate immune system interactions of human respiratory coronavirus EMC compared to those of severe acute respiratory syndrome coronavirus. *J Virol,* 87**,** 5300-4.

SUPERSEDED

13.APPENDIX 1. OVERVIEW OF DESIGN AND INITIAL RESULTS FOR IMMUNE MODULATION THERAPY COVID-19 DOMAIN AND CORTICOSTEROID DOMAIN

13.1. *Introduction*

This document describes the statistical design and analysis of the testing of interferon and corticosteroids alone and in combination in the COVID-19 appendix as part of the REMAP-CAP trial. Our goal is to investigate whether these treatments independently and/or jointly are beneficial in increasing the number of ICU- free days for patients with COVID-19. Some evidence has shown that the interaction of corticosteroids and interferon may be harmful in this setting. Our design addresses this concern by using adaptive randomization in combination with an informed Bayesian prior to reduce the number of patients placed on both arms if the interaction of the two interventions is negative.

SUPERSEDED

This document describes the reduced problem in which these two agents are assigned and we explore if the prior distribution on a negative interaction performs well for the REMAP-CAP pandemic.

13.1.1. Treatment Arms

We assume patients will be randomized to one of four regimens making up the 2x2 factorial: neither corticosteroids nor interferon, corticosteroids without interferon, interferon without corticosteroids, and both corticosteroids and interferon. At baseline, patients may be randomized to one of the four regimens. As the trial progresses, interim analyses will assess whether the active interventions should be dropped from randomization. Particular focus will be to the interaction arm due to early evidence that points to a harmful effect when taking both treatments in combination.

13.1.2. Primary Endpoint

The primary efficacy endpoint is an ordinal endpoint from –1 to 21 for the number of ICU free days (IFD), with the lowest level (–1) representing mortality. The endpoint measures the number of days out of a 21-day period the patient is not in the ICU, where 21 IFD is the best outcome and –1, death, is the worst outcome.

13.2. *Primary Analysis Model*

The primary analysis is based on a Bayesian cumulative logistic regression assuming proportional odds for intervention effects. We include an interaction effect in the analysis to model the response when patients are randomized to both steroids and interferon.

13.2.1. Model Specification

Let $Y_i = \{-1, 0,1,2,3, \dots ,21\}$ denote mortality (–1) or the number of ICU free days (0-21) for subject i. Let γ_{ik} denote the probability of subject i observing k number of ICU free days or less, $\gamma_{ik} = \Pr(Y_i \leq k)$. Then, for $k = -1, 0, 1, 2, \dots, 21$ the primary analysis model is formulated as:

$$logit(\gamma_{ik}) = \alpha_k + \theta_1 x_{i1} + \theta_2 x_{i2} + \theta_3(x_{i1} * x_{i2}) \quad (1)$$

Where $logit(\gamma_{ik}) = \log(\frac{\gamma_{ik}}{1-\gamma_{ik}})$ are the log-odds of γ_{ik}. The treatment arms are parameterized as $x_{i1}$and x_{i2}, where $x_{i1} = 1$ if subject *i* is randomized to the steroid treatment arm, $x_{i1} = 0$ if not, and $x_{i2} = 1$ if subject *i* is randomized to the interferon treatment arm and $x_{i2} = 0$ if not. For subjects assigned to the combination arm, both $x_{i1} = 1$ and $x_{i2} = 1$. For the no corticosteroid and no interferon regimen (the *no intervention* regimen), both $x_{i1} = 0$ and $x_{i2} = 0$. The primary analysis model includes an interaction term between x_{i1} and x_{i2}. The parameters α_k determine the cumulative probabilities for the no intervention regimen. θ_1 is the treatment effect for the corticosteroid intervention, θ_2 is the treatment effect for the interferon intervention, and θ_3 is the interaction effect. To estimate the effect for the combination arm, we sum $\theta_1 + \theta_2 + \theta_3$. Each θ_j are proportional odds estimates, meaning they are proportional for all *k* levels of the outcome.

We use Bayesian methods to estimate the parameters of the model. We specify independent, normally distributed priors on the log-odds treatment effects for the steroid and interferon only interventions:

$$\theta_j \sim N(0, 1), j = 1, 2 \quad (2)$$

This prior on the log odds ratio induces a lognormal prior on the odds ratio with a prior median of 1 (no effect) and a 95% prior credible interval on odds ratios 0.14 to 6.82.

Due to the concern of the possible negative effect of the interaction of steroids and interferon, we create a shifted prior for the log odds parameter of the interaction, θ_3.

$$\theta_3 \sim N(\log(0.5), 1) \quad (3)$$

SUPERSEDED

We center this prior at log(0.5), a negative effect of the interaction, with prior median of a 50% reduction in the odds ratio. The median of this prior on the odds ratio scale is equal to 0.5, the mean is equal to 0.824, and 75% of the mass of the prior falls below 1. The 95% prior credible interval is between 0.07 and 3.44. In Figure 1 below, we display the chosen prior with the median, mean, and 75th quantile displayed on the odds ratio scale ($\exp(\theta_1 + \theta_2 + \theta_3)$).

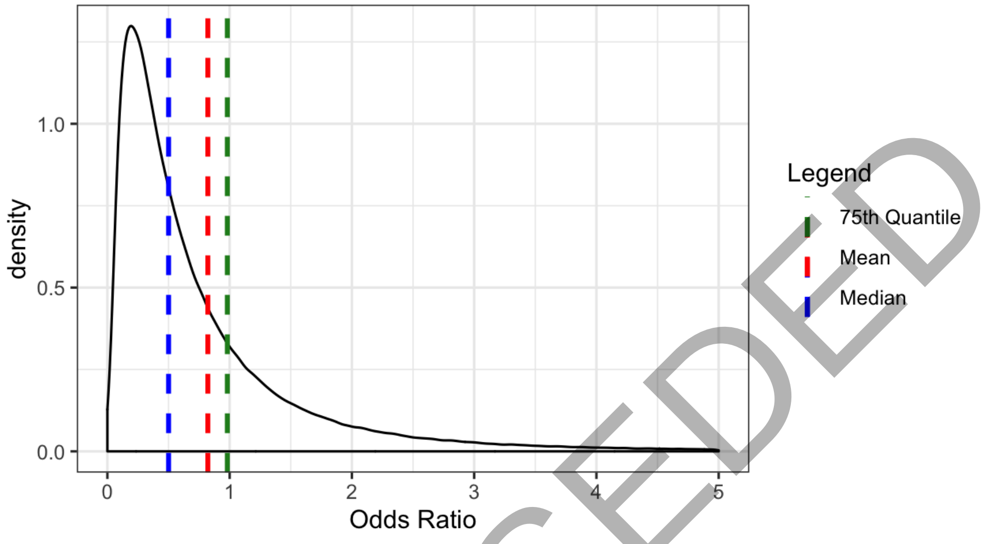

Figure 1. Density of chosen prior for interaction term in model (1)

SUPERSEDED

The prior distributions for α_k is:

$$\alpha_k \sim N(0,1) \tag{4}$$

This choice of mean and variance on the log-odds scale results in an uninformative prior, allowing the data to drive the estimated probability of observing an outcome k or lower in the control group.

13.3. *Study Design*

13.3.1. Timing of Interim Analyses

We assume the interims are timed according to the number of completed patients with 21-day endpoints recorded. The first interim analysis will occur when 25 patients have recorded their 21-day IFD endpoint. Subsequent interims occur every additional 25 patients with complete endpoint data.

At each interim analysis, response adaptive randomization (RAR) allocation probabilities will be updated using the probability each regimen is the best. In addition, each interim analysis will evaluate whether there is evidence to drop the combination regimen. If the probability of being the best regimen falls below 0.05, we drop the combination arm and continue randomizing patients to the remaining arms for the rest of the study.

13.3.2. Allocation

We begin response adaptive randomization at the start of the trial. The initial randomization probabilities are estimated using the priors set above. The resulting probabilities initialize at 25% for the no intervention regiment, 30% for the steroid only regimen, 30% for the interferon regimen, and 15% for the interaction regimen. The subsequent RAR allocation probabilities will be updated at each interim analysis. The randomization probabilities for all regimens are weighted based on Pr(Regimen j is best). We continue enrolling patients until we hit the maximum sample size of 500 patients.

13.3.3. Arm Dropping Rules for Futility

At any interim analysis, the combination regimen may be dropped from the trial for futility. The combination regimen drops when there is a low probability that this regimen is the best active regimen in this domain. The decision to drop the combination regimen for futility compared to the other active intervention is based on the posterior probability that the combination regimen is the best active arm in this domain. We calculate this probability, $\Pr(\text{Combination Regimen best})$, by comparing the joint posterior distribution of the combination regimen odds ratio to the posterior distributions of all other regimen's odds ratios. If, at any interim analysis, $\Pr(\text{Combination Regimen best})$ falls below 5%, then the combination regimen will be dropped from the trial.

13.3.4. Final Success Rule

When the 500th patient has their 21-day outcome, we estimate the probability each arm j is superior to the control arm, $\Pr(\text{OR}_j > 1)$. If $\Pr(\text{OR}_j > 1)$ > 0.99, we declare success on arm j and win the trial.

13.4. *Simulation Details*

In this section, we outline the different scenarios used to simulate virtual patient data. This allows us to evaluate the performance of the trial design across a range of possible scenarios.

SUPERSEDED

13.4.1. Control rate assumptions

We created possible control rates across the 23 levels of the outcome. We worked within a few clinically guided expected parameters: 20% mortality rate, 10% of patients are in the ICU 21 days, and median number of days in the ICU is 7 amongst those that did not die. With that, we proposed the following control rates, displayed in Table 1:

Table 1. Control outcome probabilities

Death (-1)	0.2
0	0.1
1	0.015
2	0.015
3	0.015
4	0.015
5	0.015
6	0.015
7	0.015
8	0.015
9	0.015
10	0.015
11	0.015
12	0.015
13	0.015

SUPERSEDED

14	0.015
15	0.07
16	0.07
17	0.07
18	0.07
19	0.07
20	0.07
21	0.07

13.4.2. Treatment Effects

We consider 4 different scenarios for the treatment effects on the three active interventions. Table 2 presents the scenarios in terms of the odds ratio on the active interventions.

Table 2. Treatment effect scenarios - moderate effectiveness

		Steroids	Interferon	Steroids + Interferon
**1**	Null	1.0	1.0	1.0
**2**	Interaction harmful	1.4	1.4	0.8
**3**	Interaction ineffective	1.4	1.4	1.0
**4**	All arms beneficial	1.4	1.4	1.4
**5**	One arm beneficial	1.4	1	1
**6**	Interaction helpful	1.2	1.3	1.4

SUPERSEDED

13.5. *Operating Characteristics*

For each scenario outlined in Section 13.4, we simulate 1,000 individual trials and characterize the behavior across trials. We assume a maximum sample size of 500. Table 3 describes the following information per intervention:

- Mean N: mean sample size enrolled per regimen
- Success: probability of declaring success on each intervention or overall
- Futility: probability of dropping combination regimen

We denote the no intervention regimen as arm 1, steroid only regimen as arm 2, interferon only regimen as arm 3, and the interaction regimen as arm 4. Table 3 displays the operating characteristics per intervention.

Under the null scenario, the first arm has type I error of around 4.9%, the second arm around 5.2%, and the interaction arm around 3.6%. While this is slightly inflated, additional simulations runs and simulated success boundaries will help us to tighten this closer to 2.5%. The average number of patients per arm is around 130 for arm 1 (no intervention), 151 for arm 2 (steroid only), 148 for arm 3 (interferon only), and 72 for arm 4 (combination). The lower average number enrolled on the combination arm is due to the informed prior and the arm dropping rule. In the Null scenario, we drop the interaction arm 53.4% of the time.

When the interaction arm is harmful, we essentially never declare that arm successful (0.2%) and drop it 82.1% of the time, with an average number of subjects enrolled of 20. When the interaction arm is ineffective, we declare the interaction arm successful only 0.26% of the time, drop the arm 70.4% of the time with an average number of subjects enrolled of 34. The overall power for this scenario is 67.0%. When all arms are beneficial in the moderate case, the overall power is 69.8%, with arm 2 and 3 having similar probability of success (38%), but with arm 4 only having 24.6% chance of success. The average number of subjects per arm is similar for the steroids only and interferon only arms (2 and 3) at around 178 patients per arm, and the interaction arm has 101 patients on average enrolled. When only one arm is beneficial, the trial on average enrolls most patients to the effective arm, 311, and fewer patients to the ineffective arms, 80 and 41, for arm 3 and 4 respectively. We drop the combination regimen 67.2%. The overall power for this one-arm beneficial scenario is 60.9%.

Finally, the final scenario under escalating effects, where the interaction is helpful to patients, there is an overall power of 70.9%, with the steroid arm declaring success 16.2%, the interferon arm declaring success 29.4%, and the interaction arm declaring success 30.5%. The average number

SUPERSEDED

enrolled per arm is 54 for the no intervention arm, 131 for the steroids only arm, 182 for the interferon arm only, and 133 for the interaction regimen.

Table 3. Operating characteristics per intervention

Scenario		Mean N				Pr(Success)				Pr(Drop)
		1	2	3	4	2	3	4	Overall	4
**1**	Null	130	151	148	72	0.049	0.052	0.036	0.173	0.534
**2**	Interaction Harmful	56	209	216	20	0.349	0.387	0.002	0.656	0.821
**3**	Interaction ineffective	49	211	206	34	0.390	0.376	0.026	0.670	0.704
**4**	All arms beneficial	42	178	179	101	0.375	0.376	0.246	0.698	0.440
**5**	One arm beneficial	67	311	80	41	0.521	0.028	0.029	0.609	0.672
**6**	Interaction helpful	54	131	182	133	0.162	0.294	0.305	0.709	0.379

SUPERSEDED

13.6. *Summary*

The trial design for REMAP-CAP will provide high-quality evidence for the study of the superiority of corticosteroids, interferon, and their interaction. By using prior clinical information, we created an informed Bayesian analysis that will help to protect patients from a potentially harmful interaction effect while still providing the possibility to learn more about the effectiveness of the interaction. In addition, the use or response adaptive randomization randomizes patients to the most effective arms to improve outcomes and learn about the most promising treatment. The power under the current treatment effects scenarios with 500 patients is between 61-71%. The type I error is near controlled, with arms ranging between 3.6%-5.2%. With further simulation, we will be able to find an improved success threshold to tighten the type I error closer to 2.5% controlled.
